# Saving the local tradition: ethnobotanical survey on the use of plants in Bologna district (Italy)

**DOI:** 10.1186/s13002-024-00664-1

**Published:** 2024-03-12

**Authors:** Ilaria Chiocchio, Lorenzo Marincich, Manuela Mandrone, Simona Trincia, Clarissa Tarozzi, Ferruccio Poli

**Affiliations:** 1 Department of Pharmacy and Biotechnology (FaBit), Alma Mater Studiorum, University of Bologna, Via Irnerio 42, 40126 Bologna, Italy; 2 Department for Life Quality Studies, Alma Mater Studiorum, Rimini Campus, University of Bologna, Corso d’Augusto 237, 47921 Rimini, Italy

**Keywords:** Ethnobotany, Bologna district, Traditional medicine, Nutraceutical, Natural cosmetics, Rituals

## Abstract

**Background:**

Traditional knowledge about plants is unfortunately subjected to a progressive loss, mainly due to globalization and depopulation of the rural areas. This work enhances the ethnobotanical knowledge from Northern Italy, specifically Bologna district, and contributes to preserving Italy’s plant-based traditional knowledge and to valorize local resources also in view of an ecological transition.

**Methods:**

The study was conducted between 2010 and 2016 in Bologna district encompassing 22 municipalities, which were grouped into three areas: hill, mountain, and plain. In total, 1172 key informants were interviewed, ranging in age from 50 to 85 years, and having strong links with traditional activities in the area.

**Results:**

The final inventory included 374 taxa belonging to 91 families. Among these, 251 were wild native, 40 wild alien, 74 cultivated and 6 were products bought from the market. Hill, mountain, and plain provided information on 278, 213, and 110 taxa, respectively. The most cited families were Asteraceae, Lamiaceae, and Rosaceae. The information was systematized in 12 use categories (UC): medicinal (MED), food, cosmetic, domestic, superstitious–magical–religious (SMR), agropastoral, craft, repellent-insecticide, veterinary, toxic, games, other uses and information. The most relevant UC were in turn divided into subcategories. A descriptive table with all the results was also created. MED was the most relevant UC (310 taxa), and among the 17 MED subcategories, the most significant ones were: gastroenteric (160 taxa), respiratory (133 taxa), and dermatologic (122 taxa). Food was also relevant (197 taxa, and 16 subcategories), and the widest food subcategory was nutraceutical (98 taxa). In cosmetic, the most relevant subcategory was skin treatment (37 taxa). Within SMR, the majority of the plants were cited to heal a disease in a ritual or superstitious way (15 taxa), while for agropastoral, the majority of the taxa (29) were cited as feed.

**Conclusions:**

The data collected has highlighted a significant traditional use of plants in Bologna district. Some plants or uses emerged for the first time from an ethnobotanical study carried out in Italy. The inclusion of a large number of municipalities and informants enabled the collection of a wide spectrum of data, encompassing various uses, anecdotes, and historical curiosities, which are crucial to preserve from being forgotten.

**Supplementary Information:**

The online version contains supplementary material available at 10.1186/s13002-024-00664-1.

## Introduction

The striving to push toward a more sustainable development has generated a growing interest in plants, ecosystems, circular economy, and green practices. All these topics are closely linked to the knowledge about the traditional uses of plants. This is one of the reasons why, nowadays, ethnobotany studies should be considered more and more important, making possible the redevelopment and conservation of cultural heritage, promoting the valorization of local resources, and consequently raising awareness on the importance of protecting plant biodiversity.

Until the nineteenth century, the Italian economy was mainly based on agriculture, and in this context, the knowledge of plants played a central role. In fact, plants were not only used as food and feed but also as medication for humans and animals, and for several other uses such domestic, agropastoral, and crafts, encompassing almost all the aspects of life. Additionally, plant ritualistic and superstitious uses were also an important aspect of this body of knowledge, which should not be overlooked.

The depopulation of rural areas in Italy began in the second half of the nineteenth century due to the agricultural crisis. This phenomenon became even more significant after the Second World War, as the economy underwent industrialization and agriculture became mechanized. As a result, the traditional knowledge of rural areas began to lose importance, and unfortunately, today, this information is at risk of being completely lost.

The mechanization of agriculture has had a negative impact also on plant biodiversity, unlike the traditional practices, which were contributing significantly to the stimulation of biodiversity, generating high environmental heterogeneity [1]. For instance, the abandonment of grazing, leading to reforestation, and the adoption of intensive agriculture have reduced the availability of habitats for many species [2].

The Bologna district, located in Emilia-Romagna region of Northern Italy, remains still underexplored from an ethnobotanical perspective. In fact, in this area, only one study has been published, and it focuses solely on some food plants [3].

The Bologna district is a heterogeneous area characterized by three geographical zones (plain, hill, and mountain) which differ in social, economic, and environmental aspects. In order to assess the ethnobotanical knowledge of the district, we considered various municipalities covering all the three geographical areas. These traditions are mostly kept by a small portion of the population, primarily the elderly. Therefore, the objective of this work was to systematize, preserve, and help to disseminate the traditional knowledge on plants in Bologna district, safeguarding it from the risk of being lost.

## Methods

### Area

**Fig. 1:**
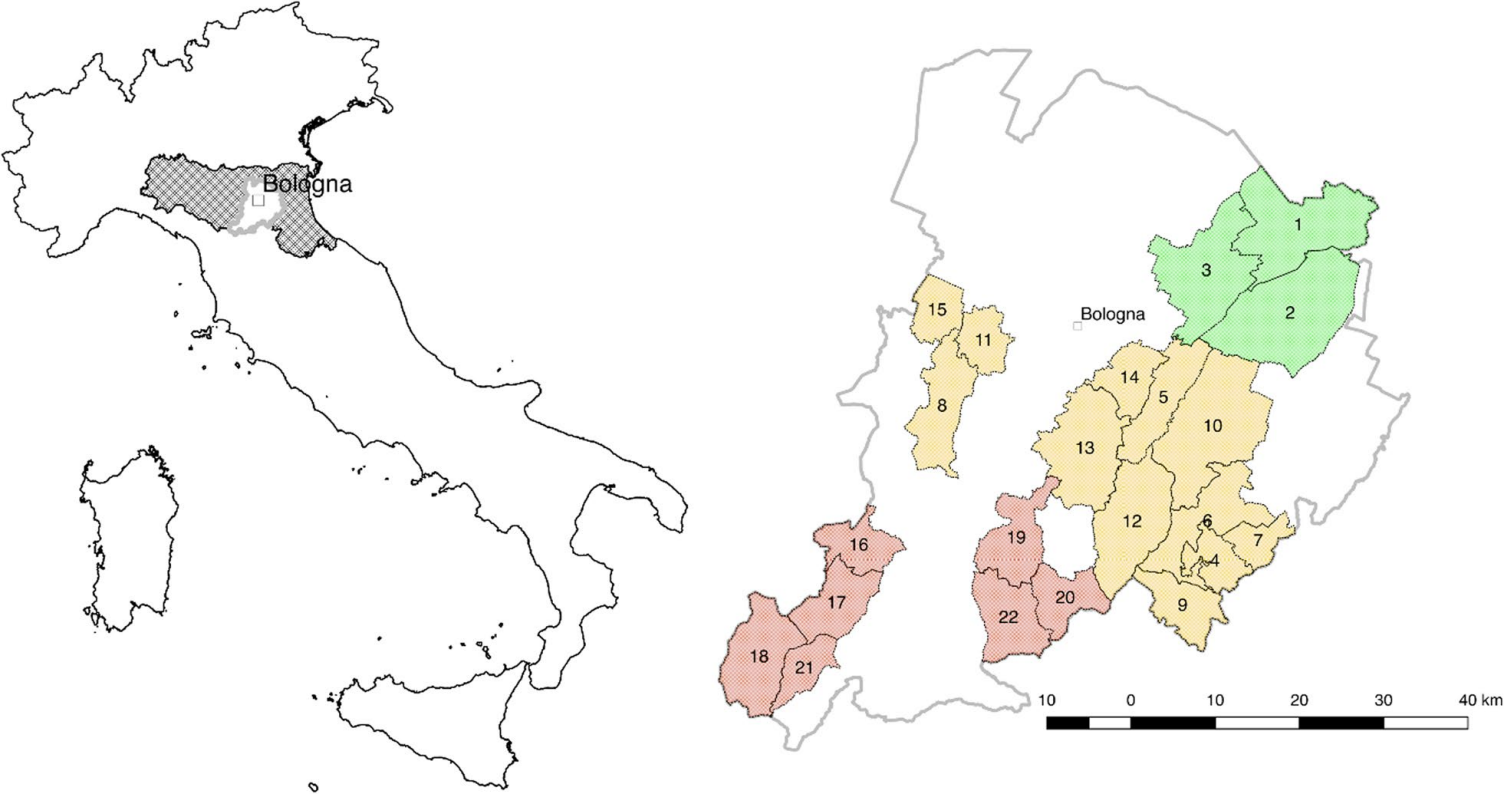
Map of Bologna district and the investigated area. Green = plain area, yellow = hill area, red = mountain area. The investigated municipalities are: (1) Molinella, (2) Medicina, (3) Budrio, (4) Fontanelice, (5) Ozzano dell’Emilia, (6) Casalfiumanese, (7) Borgo Tossignano, (8) Monte San Pietro, (9) Castel del Rio, (10) Castel San Pietro Terme, (11) Zola Predosa, (12) Monterenzio, (13) Pianoro, (14) San Lazzaro di Savena, (15) Crespellano (currently included in the municipality of Valsamoggia), (16) Caste d’Aiano, (17) Gaggio Montano, (18) Lizzano in Belvedere, (19) Monzuno, (20) Monghidoro, (21) Porretta Terme (currently included in the municipality of Alto Reno Terme), (22) San Benedetto Val di Sambro

This study was carried out from 2010 to 2016 in 22 municipalities representative of the Bologna district (Fig. 1). These municipalities, with a total area of 1811.4101 km^2^, represent the 48.9% of the entire area of Bologna district. Areas crossed by the highway, highly industrialized, or scantly inhabited were excluded. The municipalities were classified into three categories: plain, hill, and mountain areas, based on data from the Italian National Institute of Statistics (https://www.istat.it/it/archivio/156224) [4]. A number of inhabitants, GPS coordinates, altitude of each municipality are reported in Table 1.

**Table 1:** Details of the municipalities and the informants involved

N	Municipality	Area	Altitude (m a.s.l.)	GPS coordinates	Total inhabitants	Range of Age	M	F	Total interviewees
1	Molinella	P	8	44°37′03″ N; 11°40′01″ E	15,821	55–87	24	25	49
2	Medicina	P	25	44°28′43″ N; 11°38′17″ E	16,508	64–95	24	31	55
3	Budrio	P	25	44°32′14″ N; 11°32′03″ E	17,769	55–87	32	17	49
4	Fontanelice	H	165	44°15′38.16″ N; 11°33′36.72″ E	1911	63–94	14	14	28
5	Ozzano	H	67	44°26′39.372″ N; 28′31.872″ E	12,600	57–90	27	50	77
6	Casal Fiumanese	H	125	44°17′52.08" N; 11°37′30″ E	3440	50–85	20	20	40
7	Borgo Tossignano	H	102	44°16′56″ N; 11°35′47″ E	3313	60–85	14	28	42
8	Monte San Pietro	H	112	44°26′16.72″ N; 11°7′53.42″	11,020	50–90	51	46	97
9	Castel del Rio	H	215	44°12′55.08″ N; 11°30′17.64″ E	1268	51–97	17	18	35
10	Castel San Pietro Terme	H	75	44°23′58″ N; 11°23′54″ E	20,633	64–95	28	17	45
11	Zola Predosa	H	74	44°29′26.27″ N; 11°13′9.80″ E	18,097	53–90	17	41	58
12	Monterenzio	H	207	44°19′38″ N; 11°24′23.76″ E	5970	52–89	25	51	76
13	Pianoro	H	200	44°23′20″ N; 11°20′33″ E	17,231	50–88	32	28	60
14	San Lazzaro di Savena	H	62	44°28′32″ N; 11°24′54″ E	31,184	55–85	57	99	156
15	Crespellano (currently reunited under the municipality of Valsamoggia)	H	182	44°30′06″ N; 11°05′12″ E	9833	60–100	7	30	37
16	Castel d'Aiano	M	805	44°16′39.036″ N; 11°0′3.492 E	1990	51–96	16	15	31
17	Gaggio Montano	M	682	44°11′52.44″ N; 10°56′2.04″ E	5154	59–85	23	20	43
18	Lizzano in Belvedere	M	640	44°9′40.86″ N; 10°53′38.69″ E	2410	58–91	19	31	50
19	Monzuno	M	621	44°16′41″ N; 11°16′00″ E	6477	50–96	14	24	38
20	Monghidoro	M	841	44°13′38.64″ N; 11°19′42.60″ E	3901	56–87	8	11	19
21	Porretta Terme (currently reunited under the municipality of Alto Reno Terme)	M	349	44°9′36.36″ n; 10°58′23.88″ E	7051	59–85	34	28	62
22	San Benedetto Val di Sambro	M	602	44°12′58.32″ N; 11°14′2.04″ E	4522	52–90	10	15	25

Altitude, classification in P = plain, H = hill, and M = mountain; GPS coordinates; total number of inhabitants (at the time the interviews were carried on); and gender distribution, range of age of the interviewees, and total number of interviewees. The first column indicates the numeration consistent with the ones reported in the map given in Fig. 1

#### Plain

In this study, the plain area included 3 municipalities: Molinella, Medicina, and Budrio, with a total area of 407.1371 km^2^ (26.3% of total plain area of the district). The total number of informants was 153 (52.63% male and 47.37% female). All the municipalities have an urbanization level of 3 (rural zone, low population density) [4].

#### Hill

For the hill area, 12 municipalities were considered: Borgo Tossignano, Casalfiumanese, Castel del Rio, Castel San Pietro Terme, Fontanelice, Monterenzio, Monte San Pietro, Ozzano dell’Emilia, Pianoro, San Lazzaro di Savena, Zola Predosa, and Crespellano (currently included in the municipality of Valsamoggia) with a total area of 961.491 km^2^ (70.5% of total hill area of the district). The total number of informants was 751 (41.15% male and 58.85% female). All the municipalities have an urbanization level of 3 [4], except for San Lazzaro di Savena and Zola Predosa, where the urbanization level is equal to 2 (intermediate population density) [4]. Within this zone, three natural protected areas are located: Parco dei Gessi Bolognesi e Calanchi dell’Abadessa (48.1587 km^2^), Parco dell’Abbazia Monteveglio (8.7831 km^2^), and partially Riserva Naturale Contrafforte Pliocenico (7.5740 km^2^).

#### Mountain

For the mountain area, 7 municipalities were considered: Castel d’Aiano, Gaggio Montano, Lizzano in Belvedere, Monghidoro, Monzuno, San Benedetto Val di Sambro, and Porretta Terme (currently included in the municipality of Alto Reno Terme), with a total area of 442.782 km^2^ (56.1% of total mountain area of the district). The total number of informants was 268 (46.27% male and 53.73% female). All the municipalities have an urbanization level of 3 [4]. Two natural protected areas are located within this zone: Parco del Corno alle Scale (49.7449 km^2^) and partially Riserva Naturale Contrafforte Pliocenico (7.5740 km^2^). 

### Interviews

The informants were born and raised in the area. They were all baptized catholic and of Caucasian ethnicity. All of them were over 50 years of age, with limited education, and many of them were retired. Table 1 reports age and gender distribution of the interviewees. People under 50 of age were not involved in this study, as they possessed scant or no traditional knowledge of plants, in fact, when asked to be interviewed they were just recommending us to interrogate the older people of the village. In fact, initially, people were randomly chosen, and then, additional informants were selected with the help of those who had already participated.

The interviews took place at various locations, including social centers, retirement homes, farms, mountain huts, parks, bars, and during village festivals. They lasted around 30 min, during which it was took note (in a written form) of all the information given divided by plant. The anonymity of the interviewees was respected during the data collection process. The work done agrees with what is stated in the Code of Ethics of the International Society of Ethnobiology (ISE) [5].

The plant species mentioned by the informants were identified according to Pignatti [6], and the scientific names were updated using The World Flora Online (WFO) Plant List [7]. In fact, the plant names were given most of the time in the dialect of Bologna (which can have minoritarian inflection variations). The transcription of dialectal names in Table 2 was based on Ungarelli [8], Lepri and Vitali [9], and Boni and Patri [10]. Voucher specimens of the identified plants were deposited in the Herbarium of Alma Mater Studiorum-University of Bologna (Index Herbariorum: BOLO) [11] and are reported in Table 2.

**Table 2:** Summary of the detailed information about the taxa, emerging from the ethnobotanical investigation

Scientific name and Voucher Specimens	Common names	Status	Uses
*Abies alba* Mill. BOLO0002913	Abete bianco Abæd	Wild-native	*MED:* **Bud infusion** is a remedy for cold (1H) and bronchitis (1H), **cone decoction** cures chilblains (1 M), and **resin ointment** softens cracked (1 M)
*Acer campestre* L. BOLO0000996	Acero campestre	Wild-native	*SMR: ***A piece of wood** kept in the pocket keeps “evil eye” away and brings good luck (2P). A pagan wedding might be celebrated with the spouses dancing around the tree, for three times, saying magic spells (1P)
*Achillea millefolium* L.BOLO0052435	Achilea Mellfoj Melfòi Erba vturena Méllfoí Êrba di tâj	Wild-native	*FOOD:* **Flowers** (2H) **and leaves** (2P) are eaten in salad since they have digestive properties (2H). **Flowers** are eaten fried (1P). **Dried leaves** are used to prepare a relaxing bitter tea (1P). A digestive liquor is prepared by macerating **flowers** (1H, 1 M) **and leaves** in alcohol (7H). **Seeds** are added in wine demijohns as preservatives (1 M) *MED:* **Leaf decoction** calms stomachache (1P). The **infusion of leaves and flowers** is useful against dysentery (6H), calms stomachache (2H), and used in **compress** is wound-healing (6H). **Cataplasm** of young **leaves** (1 M) or **decoction of flowering tops** in **compress** (2H) are used to treat skin sores and cracks. **Boiled flowers and leaves in compress** help circulation and treat hemorrhoids (1 M). **Flower infusion in compress** is used against hemorrhoids (7H), and to clean small wounds (1H), the **infusion *per os*** is digestive (1H, 1 M), useful in case of cold, flu, and inflamed throat, since it is anti-inflammatory (2 M), together with mint, honey, thyme, and the lime tree it is used in **cataplasm** to treat pimples (7H). **A flower-based cream** is used to heal wounds (2H). **Flower ointment** is used to treat hemorrhoids (1H). **Infusion of leaves and dry roots** is used **in compress** for acne treatment (1 M) *COSM*: **Flower infusion** is added to a warm bath to contrast cellulitis (1 M) *SMR:* It is a magical plant, that protects from hex, purifies people and places from malefic influence, and is used to predict the weather (1H) *AGROPA:* The **whole plant** is used to feed animals (1P) such as rabbits (3H)
*Acorus calamus* L. BOLO0006696	Calamo aromatico Cudrés	Wild-alien	*MED:* **Rhizome decoction** is used in **compress** to relieve bone pain (2H)
*Adiantum capillus-veneris* L. BOLO0049554	Capelvenere Capælvæner	Wild-native	*MED:* The **decoction of dried fronds** is a remedy for asthma (1 M), and cough (2 M), is a nasal decongestant in case of cold (1 M), is anti-inflammatory for sore throat (1 M), and has emmenagogue activity (1 M) *COSM:* The water obtained from the **decoction** of **dried fronds** is cooled and used as a remedy for hair loss (2 M) and dandruff (1 M)
*Aesculus hippocastanum* L. BOLO0001211	Ippocastano Marån d’Endia Castagna mata Maråṅ d'Endia	Wild-alien	*MED:* **Seed decoction** is drunk to reduce hemorrhoids (4H). **Boiled seeds** if eaten are laxative (4H). **Dried fruit decoction** is astringent and anti-diarrheal (1 M). **Bark decoction** is used in case of nosebleeds (2H) and **bark infusion** is drunk in case of hemorrhoids (2 M). **Powdered seed cream** is used to improve blood circulation and reduce varicose veins (3H) *COSM:* **Powdered seed** together with soap gives a **cream** useful against skin spots (8H) *SMR:* “Nuts” **(seeds)** kept in the pocket bring good luck (2 M), and keep flu and cold away (3P, 18H, 1 M). Placing three nuts on the bedside table keeps flu and cold away (2 M) *REP:* **Seed macerated** for 24 h is an insecticide (4H)
*Aethusa cynapium* L. BOLO0030997	Cicuta minore Prezzemolo selvatico	Wild-native	*TOXIC:* It is a toxic plant that looks like parsley (except for having bigger leaves). For this reason, it is important to pay attention when harvesting parsley, since birds could have spread the seeds of this toxic plant in the field (1 M)
*Agrimonia eupatoria* L. BOLO0052599	Agrimonia Agrimônia Erba dla vòs	Wild-native	*MED:* **Dry leaf infusion** is used for hoarseness and in case of voice loss (1 M). **Flower macerated** few days in a mixture of **water and alcohol**, is filtered and drunk to purify the blood (1H, 1 M). **Flower infusion** with honey and rose leaves is used in case of fatigue during swallowing (2H) *SMR:* The plant is considered a protection from evil; **flowers** are used to fill small bags as protection from negative energies, and the **whole plant** is burned in rituals (1H)
*Ailanthus altissima* (Mill.) Swingle BOLO0052807	Ailanto	Wild-alien	*MED:* **Bark infusion** is antiseptic and it is used against diarrhea and intestinal parasites (3H)
*Ajuga chamaepitys* (L.) Schreb.BOLO0048969	Erba biga Èrba Biga Êrba bîga	Wild-native	*FOOD: ***Leaves** are eaten in salad or soup for their purifying properties (3H) *MED:* **Aerial part infusion** is used to calm dysentery (3H), as emmenagogue (3H), and to heal wounds (3H) *SMR:* This plant is believed to heal all kinds of illness (6H)
*Ajuga reptans* L. BOLO0004919	Erba biga Bugula Erba d'sant Albert	Wild-native	*MED:* **Aerial part infusion** (also in **compress**) is used to relieve skin irritations (1H). Aerial part **oleolite** (in olive oil) is used for inflamed joints (1H)
*Alcea rosea* L. BOLO0039772	Malvarosa Malvon Malvån Beladona Malvån	Wild-alien	*MED:* **Flower infusion** together with milk (1H) or **flower decoction** (1H) is used for sore throat (1H). The **infusion** of** leaves and flowers** is emollient (1H) and useful to soothe the oral cavity (1H). **Root decoction** calms cough (2H), is expectorant (3H) and its **fumes** are useful to treat bronchitis (3H). **Root and flower infusion** is used to treat dental abscesses (1H) and for eye **rinse** in case of conjunctivitis (1H)
*Alkekengi officinarum* Moench BOLO0029221	Alchechengio Balunzin ròs Vessicæria	Wild-native	*FOOD: ***Fruits** are eaten as a dessert or used to make a jam (5H) *MED:* **Fruit infusion** or **raw fruits** are diuretic (3H). **Fruit juice** is laxative (1H). **Dried berries** (without the flower calyx) are powdered and placed on the legs in case of edema, due to sitting in the proximity of the braziers (1 M) *TOXIC:* **Green parts** of the plant are poisonous (5H)
*Alliaria petiolata* (M. Bieb.) Cavara & Grande BOLO0003342	Alliaria Erba di âj	Wild-native	*MED:* **Crushed fresh leaves** are applied on the skin as an anti-inflammatory, to treat sores and acne (1 M)
*Allium ampeloprasum* L. BOLO0006610	Porro Pòrr	Wild-alien	*FOOD:* **Whole plant** is used to cook several dishes (1P, 2H). **Soup** of leek is useful against arthritis (2H) and gout (2H) *MED:* The dens mixture obtained by **boiling** the leek is helpful in the case of wrynecks (1H)
*Allium cepa* L. -	Cipolla Zivålla Zìvålla	Cultivated	*FOOD:* Onion is widely used in cookery to prepare several dishes (14H), and it is one of the most used flavors (8H). **Bulb** is eaten in salad since it has disinfecting properties for the throat and oral cavity (2P, 2 M), and for its beneficial effect on the intestine (1 M). It ameliorates blood circulation (2P, 1H), has diuretic proprieties (1H, 2 M), it is used for rheumatism (3 M), it is purifying for the liver (1 M), and is a sleep-promoting agent (2 M). Generally, eating the raw bulb is considered fortifying (2 M) *MED:* **Bulb** is eaten against tapeworm (3H). **Bulb (row or marinated vinegar)** disinfects the throat (1H). A **clove of bulb** calms the itch if rubbed on a mosquito bite (1P, 9H, 1 M). **Bulb slices** are applied on insect (1 M) (bee 1H) stings to relieve the pain. The **film between the bulb layers** is put on wounds for its high disinfectant power (1 M). **A boiled bulb** together with potatoes is used in compress in case of toothache (1H). A boiled bulb has laxative properties (3H, 1 M), lowers blood pressure (1H, 1 M), and is used in **cataplasm** to calm stomachache (8H). **Cataplasm** obtained by mixing **bulb** and butter is helpful against hemorrhoids (2H). **Bulb juice** is disinfectant (1 M) and is used on burns (3H), on insect bites (1 M), and to reduce the itch due to insect and spider bits (1H), it is used in case of cough (1H) or fever (1H). **Bulb infusion** helps to lose weight for its laxative properties (2H), it is used in case of colitis as a refreshing agent (1H), and as a diuretic (2H). **Bulb decoction** has diuretic properties (3H), and it is drunk to purify and fortify the body (3 M). The **bulb** is **boiled in milk,** and it is drunk against cough (1P). **Syrup** made with onion, sugar, and honey cures colds (3H). **Leaf in compress** is used in case of arthrosis (1H). **Leaves** are used to treat wounds (2P) *SMR:* Twelve **bulbs** were traditionally used to predict rainy and dry months (1P, 1 M)
*Allium sativum* L. -	Aglio Aj Ai Âj	Cultivated	*FOOD:* Garlic is widely used in cookery (18H, 5 M), and in dishes such as “agliata” (a soup with bins) (1 M). **Garlic** are used to flavor some dishes (9H) *MED:* **Garlic** is given to eat (8H, 2 M) or smell (1P, 3H, 2 M) to kids to prevent intestinal parasite infection. **Raw garlic** is eaten to combat intestinal parasites (1H) and as an antidote for viper bite (1H). **The bulb** lowers blood pressure (2P, 30H, 6 M), it improves blood circulation (1H), is beneficial for the heart (1P), and it reduces cholesterol (2P, 2H). To chew the bulb calm toothache (2P) and sore throat (1 M). A clove of the bulb is kept in the mouth or in the nose against cold (1H). A clove of garlic is rubbed on ears to calm otitis (2H), on cold limbs to reduce chilblains (3H), and on the nose against cold (2H). **The bulb** is crushed in a mortar to obtain a **juice** useful to reduce calluses (2H, 2 M), warts (1 M), insect bites (1H, 3 M), and in case of viper or dog bites (1H). Ten **bulbils are crushed** and chopped in half a cup of **oil**, this semisolid preparation is rubbed on sore bones (1 M). The minced bulb **in cataplasm** is used to calm pain (1P), in particular neuralgia (1P). A **paste** obtained with a clove minced with breadcrumbs and a bit of milk is used to treat whitlow (“giradito”) (1 M). **Cloves** of garlic are crushed and mixed with olive oil to obtain an **ointment** used topically on the belly for digestive issues (4H), to eliminate intestinal parasites (2H), applied under the feet soles to lower the blood pressure (3H), and used to treat ear infection (2H). **Bulb macerate in water** is anti-lice (1H,) and calms insect bite itch (1H). **Compress of bulb macerated in water** is used to heal wounds (1 M). **Bulb boiling water** together with vinegar is used for gargles in case of toothache and gumache (3H). **Bulb boiled in milk** with honey is an expectorant (1P) and fights intestinal parasites (2H). A teaspoon *per* day of garlic **macerate in alcohol** is useful to prevent arteriosclerosis (1H). The **external part of the bulb** disinfects wounds (2P) *COSM:* Garlic macerated in alcohol is used to cure alopecia (2H) *SMR:* A clove of garlic in a glass protects from the diseases (1H), if kept under the pillow it is a remedy against intestinal parasitosis (3H). **Braids** made of garlic are hanged in the house to keep witches away (1P), and garlic wreath with red ribbon is used against “evil eye” (1 M). A **garlic necklace** keeps worms, snakes (2H) and intestinal parasites (15H) away *REP:* **Grounded garlic** in water sprayed on roses keeps aphids away (1 M)
*Allium schoenoprasum* L. BOLO0008427	Erba cipollina	Wild-native	*FOOD:* **Aerial parts** are used to prepare several dishes (5P), and as spice (2 M) *MED:* **Aerial part** has diuretic properties (1 M). It is eaten to heal from intestinal worms (1H). Aerial part **infusion** regularizes heartbeat (1H), and it is used in callus-reducer footbaths (1H). **Leaves wraps** are wound-healer (2H) *REP:* Surrounding the windows with the **leaves** keeps insects away (1H)
*Alnus glutinosa* (L.) Gaertn. BOLO0003359	Ontano Ontan	Wild-native	*MED:* **Bark decoction** is used against sore throat (1P). **Leaves** are hemostatic in case of small wounds (1P)
*Aloe vera* (L.) Burm.f. BOLO0007896	Aloe vera	Cultivated	*MED:* **Leaf juice** heals wounds (1H), protects from tumors if it is swallowed (1H), and makes the body more tonic and active (1H). It is used to massage the back in case of joint inflammation (4H). **Leaf infusion** reduces pain due to ulcers (1H)
*Aloysia citrodora* Paláu BOLO0011975	Erba citronella Erba Luigia Erba cedrina Cedrina Èrba zìdreina Êrba zedrenna Êrba zedrẹṅna	Cultivated	*FOOD:* **Aerial parts** are widely used in cookery as flavor and to prepare liquors (11H). **Leaves** are used as flavor (4H) and are eaten for the digestive effect (4H). **Leaves** are used to prepare digestive liquors (1 M, 1H), that stimulate appetite and reduce intestinal gasses (1H) *MED:* **Leaves** are rubbed on teeth and gums to fight halitosis (2H). **Leaf infusion** disinfects the stomach (3H), lowers the fever (3H), and is relaxing (3H). **Liquor in fumes** calms cold (1H) and sore throat (1H) *DOM:* **Flowers** are kept in the armchair to perfume linens (3H)
*Althaea officinalis* L. BOLO0049311	Altea Alteja	Wild-native	*MED:* **Leaf decoction** together with pomegranate leaves is astringent (4H). Kids chew the **roots** to calm teething pain (1P, 2H). **Root macerate** is used to treat cough (1H). **Root decoction** is an expectorant (3H). **Fumes** of the decoction are useful in case of bronchitis (3H)
*Anacamptis morio* (L.) R.M.Bateman, Pridgeon & M.W.Chase BOLO0003126	Concordia Fior ch’as surcen Cuncordia	Wild-native	*FOOD:* Children suck the **petals** for their sweet taste (1 M)
*Anethum graveolens* L. -	Aneto puzzolente Fnoc puzzleint	Cultivated	*FOOD:* It is used to flavor several dishes (3H). This **plant** is eaten to improve digestion (1H)
*Angelica archangelica* L. BOLO00030618	Angelica	Wild-alien	*FOOD:* **Root liquor** is digestive (1H) *MED:* **Root decoction** reduces stress (3H) and stimulates digestion, preventing aerophagia and headache (3H)
*Angelica sylvestris* L. BOLO0049237	Angelica Erba di cavéi Angiælica Angiaælica	Wild-native	*FOOD:* **Leaves and stems** are used in cookery (1H) *MED:* **Flower infusion** has digestive (1H) and purifying properties (2H), and it is useful against stomachache (12H). **Seed powder** is used against lice (3H) *COSM:* **Compress of boiled flowers and steam** is used to prevent baldness and to strengthen the scalp (1 M)
*Anthyllis vulneraria* L. BOLO0046805	Vulneraria Trifoiel seivadg Trafujôla	Wild-native	*MED:* A **compression wrap** containing **shredded flowers** is used as a wound healer (1 M). **Flower and leaf decoction,** filtered for about 20 min, is used **in compress** for wound healing, burning, and abrasions (1 M) *AGROPA:* **The plant** is used as feed for cattle (1 M)
*Antirrhinum majus* L. BOLO0003333	Bocca di leone Båcca ed låuv	Wild-native	*MED:* **Flower decoction** is used in **compress** on burns (1 M) and on skin redness (1 M) *SMR:* It was believed that anointing with **seed oil** makes a person more handsome (1 M) *REP:* To place a **spring** of this tree in the room corners to keeps scorpions away (2 M)
*Apium graveolens* L. BOLO0049014	Sedano Saerrel Særrel	Wild-native	*FOOD:* **Leaves** (1H) and **stems** (7H) are used in cookery. **Raw celery** is eaten to reduce stomach swelling (3H) *MED:* **Leaf infusion** in footbath cures chilblains (2H). **Infusion of roots** of celery, fennel, parsley and butcher’s broom reduces the intestinal gases (6H). **Fruit infusion** is used to relieve stomach and intestinal inflammation (1H) *VET:* A mixture of **leaves infusion** together with lard is used in **cataplasm** to treat cow’s mastitis (1H)
*Aquilegia* spp. -	Aquilegia	Wild-native	*MED:* **Flower infusion** is used *per os* as astringent (3 M), sedative (1 M), and topically as anti-inflammatory (1 M)
*Arbutus unedo* L. BOLO0055377	Corbezzolo Curbæzzel	Wild-native	*FOOD:* **Fruits** purify the liver (1P). **Unripe fruits** are eaten raw as diuretic (1 M), while **ripe fruits** with orange are used to prepare a jam (1 M)
*Arctium lappa* L. BOLO0022265	Bardana maggiore Lâpa Rezz	Wild-native	*FOOD:* **Leaves** are used in cookery (1H). **Roots** are used to prepare a liquor (1H). **Roots** are boiled and eaten for their detoxifying properties (2H) *MED:* **Leaves** are smoked to purify the lungs (2H). **Minced leaves** have anti-inflammatory action on skin (1P), and they are used on wasp bites (1P). **Fresh leaves** are applied on inflamed skin (1 M). **Cataplasm** of leaves in milk has cicatrizing properties (1H). **Leaf and flower macerated in oil (oleolite)** is used for skin affection (2H). The **decoction of roots** (3H) or the **infusion of leaves** (2P) are used as purifying and diuretic agents. Root decoction (a spun per day) is drunk against acne (2H) and rubella (1H). **Root decoction in compress** treats pimples (3H) and acne (2 M). **Root oleolite** is useful against acne (1 M). **Root pulp** is used as detergent in case of skin diseases (3H). **Root syrup** *per os* (two–three spoons per day) treats eczema (1H). **Seed infusion** is laxative (1H), and **seed and leaf decoction** is used to rinse the mouth in case of infections (2 M) *COSM*: **Compress of boiled leaves** is used for skin rejuvenating (2 M). The **leaves** together with nettle leaves and oil are rubbed on the scalp against dandruff (1 M). **Root pulp** is used to prevent hair loss (3H) *AGROPA*: **Leaves** are used in vegetable gardens to protect shoots and to prevent weed growth (3H). Leaves, due to their wide dimension, are used to cover shoots in vegetable gardens protecting them from high intensity sun light *GAME:* kids used to play at war by throwing **prickly flowers**, which stick on the clothes (8H)
*Arctium minus* (Hill.) Bernh. BOLO0052425	Bardana minore	Wild-native	*DOM*: **Leaves** were used as toilet paper (1H)
*Arctostaphylos uva-ursi (L.) Spreng*. BOLO0030285	Uva ursina	Wild-native	*MED:* **Leaf infusion** is diuretic (3H), laxative (3H), cures cystitis (1H), purifies the urinary tract, and prevents prostate conditions (2H)
*Armoracia rusticana* G. Gaertn., B.Mey. & Scherb. BOLO0035660	Rafano rusticano Cren Rafan	Wild-alien	*FOOD:* The **root** is used to prepare several dishes (1P)
*Artemisia absinthium* L. BOLO0022765	Assenzio	Wild-native	*MED:* **Leaves** are used to prepare a digestive **infusion** (1P)
*Artemisia dracunculus* L. BOLO0012772	Dragoncello	Cultivated	*FOOD:* **Leaves** are an ingredient of several dishes, used because they are digestive (1H). **Leaves** are crushed to flavor rennet (3H). **Fruits** are used to prevent the formation of mold in foods (1H) *MED:* **Root decoction** is used against sore throat (3H) *OUI:* In times of poverty, a high amount of leaves was chewed to remove the sensibility of taste buds, enabling to eat also the bad taste food (2H)
*Artemisia vulgaris* L. BOLO0052428	Artemisia Erba dal mosch Artemîsia Artemîʃia	Wild-native	*FOOD:* **A liquor** made with flowering tops (macerated for 20 days in alcohol (1 M)) stimulates the appetite (1 M) *REP*: Bunches hung in the stables keep flies and gadflies away (1 M)
*Arundo donax* L. BOLO0041939	Cannuccia Canòccia	Wild-alien	*MED:* **Rhizome** is used to prepare a diuretic **decoction** (1P)
*Asparagus acutifolius* L. BOLO0053689	Asparago selvatico Sparazèna Sparzeina Sparazèna Sprez Sparzeṅna Sparzenna Spèrż	Wild-native	*FOOD:* **Shoots** are eaten cooked (5P, 26H) *MED*: The **shoots** have diuretic properties (8P, 7H), and purifying activity (1P). They are urinary tract disinfectant (1P), kidney protective (1P), digestive (6H), kidney cleaner (1H), and bladder cleaner (1H). **Asparagus cooking water** relieves rheumatic (2H) and gout pain (2H). **Young shoots** (4H) **and decoction of roots** (4H) are used for kidney and bladder conditions
*Asplenium ceterach* L. BOLO0052408	Cedracca comune Asplenio cedracca Cedracc Erba ruggine Spaccapietre	Wild-native	*MED*: **Root decoction** is a remedy against cough (1H). **Fronds boiled in wine** prevent kidney stones (1 M)
*Asplenium viride* Huds. BOLO0021179	Asplenio Felcetta Êrba dal dôn	Wild-native	*MED*: **Leaf infusion** is febrifuge (1 M). **Frond decoction** is useful against bronchitis (1 M), as expectorant and antitussive (1 M)
*Atropa belladonna* L. BOLO0029263	Atropa Belladonna Bæladôna	Wild-native	*MED*: **Root and leaf decoction** calms stomachache (3H). **Leaf and flower infusion** is drunk in low dosages as a calming agent and sleep inducer (1 M) *OUI*: The name “belladonna”, which means “beautiful woman”, derives from the dilating effect on pupils given by this plant; since this feature was considered glamorous in women, the **fruits** were eaten in small dosages to obtain this effect (1 M) *TOXIC:* **Fruits** are poisonous (2H)
*Avena sativa* L. BOLO0041969	Avena Væna Væina (A)vä\na	Cultivated	*FOOD:* **Fruits** are used to prepare bread (2H, 1 M) and to obtain liquor (2H) *MED*: **Fruit decoction** has restorative (1H), laxative (1H), and calming properties (2H). **Leaf infusion** is used to wash the oral cavity in case of sore throat (1H). **Leaf decoction** in combination with basil leaves is used for tonsillitis (1 M) *GAME:* Kids throw the spikes of oats at each other for they stick to their clothes (4H). **Flowers** are used to make whistles (1H)
*Ballota nigra* L. BOLO0049078	Ballota Balota Marobbi bastérd	Wild-native	*MED*: **Decoction** of the **whole plant** is a remedy for ringing in the ears (1 M)
*Barbarea vulgaris* W.T.AitonBOLO0052362	Barbarea Rocla seivadga	Wild-native	FOOD: **Leaves** eaten in salads cure the gouty foot (1 M) *MED*: Shredded **fresh leaves** in **cataplasms** are used to treat knee scarring (1 M). Crushed **seeds** mixed with wine are used as diuretic (1 M)
*Bellis perennis* L. BOLO0053702	Margherita comune Margaritenna Margherètta	Wild-native	*FOOD:* **Leaves** are eaten in salad (2P), **buds and petals** are used as spices (1 M). **Flower infusion** is a restorative summer drink (1 M) *MED*: **Flower infusion** purifies the blood (2 M), prevents hepatic conditions (2 M), is diuretic (3 M), and is used externally to treat dermatological conditions (1 M), rheumatisms (1 M), and to heal wounds (1P)
*Beta vulgaris* L. BOLO0055355	Bietola Barbabiáttla	Cultivated	*MED:* **Boiled leaves** are eaten (3H) for their purifying action on the liver (3P). **Boiled leaves** and **stems** are laxative and emollient for the intestine (3H) *AGROPA:* **Leaves** are used to enhance milk production in cows (1P)
*Betula pendula* Roth BOLO0017008	Betulla Bdòll	Wild-native	*MED:* **Dried leaves** are boiled in water for few minutes and filtered; the **infusion** obtained is diuretic (3 M), depurative (3 M), useful for kidney stones (1H, 1 M), arthritis (1 M), cystitis (1 M), and to heal inflammatory conditions (1 M). **Fresh leaf** (harvested in spring) **infusion** is diuretic (1 M) *COSM:* The cooled **water of leaf infusion** is used in **compress** against cellulitis (2 M). During spring, holes are made in the trunk (in the part of the tree facing at south) to extract the **sap**, which is sprinkled on the legs to drain and to prevent cellulitis (2 M)
*Betula pubescens* Ehrh. BOLO0000640	Betulla Betuja	Wild-native	*MED:* **Leaf infusion** treats infections of the urinary tract (2H) and edemas (2H) *COSM*: **Leaf cream** reduces cellulitis (2H)
*Borago officinalis* L. BOLO0053721	Borragine Burâzen Burâzen	Wild-native	*FOOD:* **Whole plant** is eaten and used to prepare several dishes (3P), **tender leaves** are eaten in omelets (2 M, 1H), soups, rolls (3H), and in salad (1H), **flowering top** is eaten in “tortellini” (a traditional dish) (1H) *MED:* **Leaf infusion** lowers temperature (in case of fever) by increasing sweating (5H), it is also diuretic (2H). **Leaf and flower oleolite** is used for skin redness and skin itching (1H). **Flower infusion** is diuretic (3H) and purifies the liver (3H), it is used as antitussive (1H) and anti-catarrhal (1H) *COSM*: **Leaves and flowers oleolite** (in olive oil) is used for dry skin (1H)
*Brassica napus* L. BOLO0008752	Rapa	Wild-alien	*FOOD:* **Roots** eaten in salad have digestive properties (2H) *MED:* **Root syrup** is expectorant (2H)
*Brassica nigra* W.D.J.Koch BOLO0049296	Senape Senape nera Radisin seivâdg Sænva	Wild-native	*FOOD:* Together with red apples it is used to prepare mustard (1 M) *MED:* **Seed cataplasm** (together with linseed (1H)) is applied on the chest to cure bronchitis (2H). An **enolite of seeds** is prepared to treat kidney stones and as a diuretic (1 M)
*Brassica oleracea* L. BOLO0002291	Cavolfiore Côl con e fior Chevàl Côl Chevolfiåur	Cultivated	*FOOD:* **Whole plant** is eaten for its restorative activity (6H) *MED:* **Raw cauliflower** is eaten to calm heartburn (1 M), and the **decoction** is a remedy for gastric and duodenal ulcers (1 M). **Leaves** are used in soothing **wraps** for the skin (3H). **Fresh leaves** are a remedy against rheumatisms (2H), they are applied on inflamed joints (5H), in the proximity of the liver area (1H), and on painful body parts in case of gout (2H). **Fresh leaf wrap** is a wound healer (2H). **Leaves** are **boiled** and applied on joints to calm pain (5P), and are wound healers (2H). **Leaf infusion** is used in footbaths to treat chilblains (1H). **Cabbage syrup** removes the excess of alcohol from the body (1H). **Decoction of leaves and flowers** is anti-anemia (1 M)
	Verza Vêrza	Cultivated	*MED:* **The external leaves** are placed between two worm patches and applied on the body part affected by rheumatism (1 M). **Cooking water** is optimal for heartburn (1 M). **Leaf pulp** heals wounds, promoting the expulsion of foreign matters (1H). **Boiled leaf wraps** are wound healers (2H). Boiled leaves bandages are applied on sore bones (3H). **Fresh leaves** are applied on reddish and inflamed skin as lenitive (4H) *COSM:* **Leaf boiling water** is used to make refreshing footbaths (1H)
	Cavolo cappuccio	Cultivated	*MED:* In case of cough and cold, **leaves** are **boiled** and applied between two towels over the sternum for their expectorant activity (1 M). **Fresh leaves** are applied on reddish and inflamed skin as lenitive (1 M)
*Buxus sempervirens* L. BOLO0001672	Bosso	Wild-native	*MED:* **Leaf infusion** is laxative (2H), it lowers the body temperature in case of fever, by increasing sweating (3H) *SMR:* It is propitiatory and apotropaic, and for this reason, during Easter a **sprig** is kept in the pocket (1 M)
*Calendula officinalis* L. BOLO0053973	Calendula Ganzant Calàndla Ganʒåṅt Ganzȧṅt Ganżànt	Cultivated	*FOOD:* Omelets **of fresh flowers** are prepared (1 M, 1H) *MED:* **Fresh leaves** are rubbed on warts (1 M). **Leaf and flower infusion** calms stomach pain (4H). **Leaves and flowers** are used on skin **in cataplasm** for their healing and soothing properties (6H), while **flower cataplasm** relieves insect bites (1 M). **Flower infusion** is used to heal intestinal conditions (3H), as anti-inflammatory (1 M), anti-spasmodic (2 M) and anti-emetic (1H), it is a pain reliever and it is used to calm menstrual pain (3H). **Cooled flower infusion** is used in **compress** on irritated eyes (2 M, 1H). **Flower decoction** is used as an anti-emetic (1H), **flower infusion in compress** cures sores, cracks (1H, 1 M), and skin irritations (2H, 1 M), **flower infusion or decoction in compress** is soothing and emollient for reddened of skin and mucous membranes and it is useful for wound healing (1H, 1 M). **Flowers oleolite** is used on burns (8H), on inflamed skin (3H), and for belly massages to calm menstrual pain (1 M). Mixing vaseline and calendula **flowers** is prepared a **skin crème**, which is soothing on insect bites, useful for skin redness and burns for its wound healing properties (4H). **Hydroalcoholic extract of flowers** is used in drops as antibiotic (1 M) *SMR:* Looking at calendula **flowers** is recommended to improve the sight, to calm the mind and to stay in a good mood (1H). **Flowers** kept in a flowerpot induce cheerfulness and a good mood (1 M) *VET:* **Leaf juice** is applied to the domestic animals’ ear as anti-worm agent (1H)
*Calluna vulgaris* (L.) Hill BOLO0047089	Erica Scôvva	Wild-native	*MED:* **Flower infusion** stops diarrhea in children (1 M) *DOM*: A twig is inserted into the ham to check the degree of progress during the curing (1 M)
*Campanula rapunculus* L. BOLO0004405	Raponzolo	Wild-native	*FOOD:* **Leaves** are eaten in salad (1P, 1H)
*Cannabis sativa* L. BOLO0009161	Canapa Chènapa	Cultivated	*DOM:* During winter, the woody part of the **stems** is burned in the fireplace (1P). An oil for lamps is obtained from the **fruits** (1P) *CRAFT*: The **fibers** obtained from the **stems** are used to make ropes (1P), and fabrics (1P). The **stem** is used to make an insulating chipboard (1P)
*Capsella bursa-pastoris* Medik. BOLO0053703	Borsa pastore Borsa de pastor Bursa da paståur Bûrsa dal paståur	Wild-native	*FOOD:* **Leaves** are used in the preparation of dishes (3H). **Seeds** are added to the bread dough for their digestive properties (1H) *MED:* **Whole plant decoction** cures fever (1P), is diuretic (1 M), and regularizes the menstrual cycle (1H, 1 M). A cotton ball is soaked in the **fresh juice** of the plant and inserted into the nostrils to stop nosebleeds (1H). A **decoction of fresh aerial pats** is used against menstrual pain (1H, 1 M), and, to regularize the menstrual cycle a spun of this decoction is drunk 8–10 days before the date in which menstruation is expected (1 M, 1H). **Fresh aerial parts,** pestled and **mixed with clay**, are applied on wounds to stop hemorrhage and promote the healing (1H). **Fresh leaves** are healers if directly applied on wounds (1 M). **Leaf** (5H) **or whole plant** (1 M) **decoction** used topically cleans and heals wounds. **Leaf infusion** is useful against dysentery (1H). **Fruit decoction** is used **in compress** to heal small wounds (1 M), to stop nasal bleeding (1 M), and it is applied on pimples (1 M) *VET:* **Whole plant decoction** stops the bleeding in cattle (1 M), and **patches** soaked in the decoction are placed on sheep nipples as anti-inflammatory to facilitate mumming (1 M)
*Capsicum annuum* L. -	Peperoncino Puvrunzeìn Pevrunzén	Cultivated	*FOOD:* **Fruits** are widely used as a spice (7H), and in general in cookery (2H). Chili is eaten to improve blood circulation in case of cardiac conditions (2H) *MED:* **Aerial part infusion** is used to calm rheumatic pains (3H), and gingival weakness (3H). **Fruits** reduce hemorrhoids pain (1H) and prevent flu (3H). A teaspoon of **fruit decoction** stops nausea and dizziness (2H). **Chili** helps digestion and improves blood circulation (9H). **Dried fruit is pulverized** and used to lower blood pressure (1H). **Chili footbath** restores body temperature (1H)
*Carlina acaulis* L. BOLO0022262	Carlina Labrasnin Cardôn	Wild-native	*FOOD:* **Raw root** is edible. It is used to treat sore throat (1 M), it is purifying for the liver (1 M), and it promotes digestion (1 M)
*Carum carvi* L. BOLO0030803	Cumino	Purchased product	*FOOD:* **Fruits** are used in cookery (1H) *MED:* **Fruit infusion** is diuretic (3H), relieves heartburns and inflammations (3H)
*Castanea sativa* Mill. BOLO0003371	Castagno Castâgn Castàgn Castagn	Wild-native	*FOOD:* **Chestnuts** are eaten cooked, or dried to make a flour used to prepare several dishes endowed with high nutritive value such as “polenta”, “frittelle” and “manfét” (2 M) and “castagnaccio” (1P, 10H, 1 M), which is very energetic (1 M). Grounded chestnut is used to make a typical dessert called “necci”, which is placed on chestnut leaves and cooked in terracotta pads on fireplace (53 M). Cooked fruits are eaten as astringent (1H), mild laxative (2H), and energizer (5H), but in large quantity they induce gum inflammation (1 M). Fruits are eaten dry with the addition of water or wine (2H) *MED:* **Raw fruits** are eaten to cure stomachache (1H) and for their laxative properties (1H). **A dry chestnut**, kept in the mouth, reduces halitosis (1H). **Flour** mixed with water is used as remedy for stomachache (1 M), ant it protects the intestine from infections (1 M). **Fruit decoction** is a remedy for cold (2H, 12 M), sore throat (1H) and early feverish symptoms (2H). **Fruit boiling water** stops diarrhea (2 M) and cough (4H). The **cooking water of fruits** (1 M) or **dried leaves** (1 M) is used to make warm footbaths against chilblains **Leaf infusion** is cough sedative (4H, 3 M), expectorant (1H) and disinfects the upper respiratory tract (4H). **Barks and leaves** dried for about three months are used in **infusion** useful against diarrhea (10 M), cold (12 M), and cough (16 M). **Bark decoction** stops diarrhea (4H) *COSM:* **Fruit boiling water** gives a brownish color to the hair (1 M). **Seed pulp** is used in cleansing face masks (1H) *CRAFT:* **Chestnut wood** is used to make poles, boxes, baskets, and fences (3 M). When the tree shows its first buds it has more lymph, so the bark is easily removed. In this period chestnut branches were cut to make “musette” or “musole”, a kind of flute (3 M). Kids make necklaces with the leaves, used as an ornament (1 M) *DOM:* Leaves are harvested in August after the first rain, dried under the sun and stored during the winter to be used in the preparation of “tigelle” (a typical bread). Specifically, leaves are put between the dough and the pan to prevent the dough from sticking during cooking (3 M) *AGROPA:* **Leaves** were sometimes used, instead of the more expensive straw, to make animal bedding (1 M) *OUI:* **Dried leaves** were used as a tobacco substitute (2 M)
*Celtis australis* L. BOLO0049262	Bagolaro Spaccasassi Parpignån	Wild-native	*FOOD:* **Fruits** are prepared in jam, and eaten once a day, to counteract both stress and depression (1H). Ripe fruits are eaten raw (1 M) *MED:* L**eaf decoction** is astringent and used in case of diarrhea and intestinal infections (1 M) *CRAFT:* The **woods** are used to CRAFT tennis rackets (1 M)
*Centaurea calcitrapa* L. BOLO0048261	Calcitrapa Cheicatreppel	Wild-native	*MED:* **Flower decoction** is a diuretic and urinary tract disinfectant (1 M) *AGROPA:* It is a pest plant, to be removed from the vegetable gardens (1 M)
*Centaurea cyanus* L. BOLO0053788	Fiordaliso Garufanin blô de grén	Wild-alien	*MED:* **Flower decoction in compress** relieves eye fatigue and conjunctivitis (1 M)
*Centaurea* spp. -	Fiordaliso	-	*MED:* After filtration, **flower boiling water** in **compress** is used on the eyes in case of stye (1 M), redness (1 M), and inflammations (1 M). **Flower infusion** is antipyretic (1 M), anti-diarrheal (1 M), cleanser of mucous membranes (1 M), and reliever of menstrual (1 M) and hepatic pain (1 M) *COSM:* Hair is washed with the **water of boiled flowers** as anti-dandruff (1 M). **Infusion of flowers** is used externally as face cleanser (1 M)
*Centaurium erythraea* Rafn BOLO0050703	Centaurea minore Erba dla fevra Êrba da la fîvra	Wild-native	*FOOD:* The **plant** is used to prepare a digestive liquor (1H) *MED:* **Flower infusion** is antipyretic (7H)
*Ceratonia siliqua* L. BOLO0014495	Carruba Carrubo Faeva marena	Cultivated	*FOOD:* Sweets and biscuits prepared with the **leaves** (2H) counteract stomach acidity (2H) *MED:* **Leaf infusion** is used against tonsillitis (2H) and stomachache (2H) *AGROPA:* **Leaves** are used to feed animals (2H)
*Chamaemelum nobile* L. BOLO0036665	Camomilla romana Camumella	Wild-alien	*MED:* An herbal tea is made with **dried aerial parts**; it is calming and sleep inducer (2 M). The **decoction** is used for vaginal douching (1 M) *COSM:* Washing the skin with **flower boiling water**, confers a bright complexion and removes spots and acne scars (1 M)
*Chelidonium majus* L. BOLO0052175	Celidonia Chelidonia Êrba di pôr	Wild-native	*MED:* The yellowish **latex** obtained from the **stem** is used to treat warts (1P, 24H, 6 M) and calluses (2 M). **Leaf infusion** is beneficial for the heart (1 M). **Leaves and flowers** together with 2 horse chestnuts (*Aesculus hippocastanum* L.) are added to baths for hands and feet, for the beneficial effect on circulation (1 M)
*Chenopodium bonus-henricus* L. BOLO0001104	Spinacio di montagna	Wild-native	*FOOD:* **Boiled leaves** are rich in mineral salts (1H) *DOM:* Cooking water is used to wash the wool (1H)
*Cichorium endivia* L. BOLO0015307	Indivia Scarola *Indivîa*	Cultivated	*FOOD:* **Leaves** are eaten as remineralizing (8H) *MED:* **Seed and leaf decoction** is laxative (2H)
*Cichorium intybus* L. BOLO0053713	Cicoria Radicchio selvatico Radećć Cicoria sambadga Radech Radećć da camp Radàcc' Radecc amér	Wild-native	*FOOD:* **Leaves** are eaten in soup (2H) or (harvested before flowering time 1 M) in salads (6P, 9H, 1 M), fresh or boiled for their digestive (4H) and laxative (1 M). properties, to promote purification of blood (3 M), intestine (1H), liver (3H, 4 M) and organism in general (2H), and to stimulate the physiological renal function (2 M). The **root** is roasted and used to prepare an alternative beverage to coffee (2P, 3H) *MED:* **Leaves** are laxative (3H), they have hepatic purifying proprieties (3P), and reduce gastric secretion (3H). **Fresh leaves** are used on ulcers and redness (1 M). **Leaf infusion** is beneficial for the liver (1 M). **The water of boiled leaves** is depurative (1H). **Leaf and root** decoction is purifying (8H). **Leaves and flowers** are used in wraps on painful body parts (1 M)
*Cirsium vulgare* (Savi) Ten. BOLO0049398	Cardo asinino Stupion	Wild-native	*FOOD:* **Tender stems** are eaten boiled (1H) *MED:* **Fresh juice** from **leaves or stems** is used to disinfect wounds (1H)
*Cistus salviifolius* L. BOLO0046881	Cisto femmina	Wild-native	*MED:* **Aerial part decoction** is digestive (1H) *REP:* **Flowers** are kept under the pillow during the night to keep spiders away (1H)
*Citrus aurantium* L. BOLO14295	Arancio amaro	Cultivated	*MED:* **Cataplasm of fruits** is used on abscesses and ulcers (3H)
*Citrus limon* (L.) Osbeck BOLO0014363	Limone Limòòn Limoun Limån	Cultivated	*FOOD:* Lemon is used in cookery (1H). **A liquor** is prepared using the peel and laurel leaves (1H). A refreshing drink is prepared with lemon **juice** (3H) or with lemon slices (1H), and it is rich in C vitamin (1H) *MED:* **A slice of lemon** covered with salt is used against herpes (2H). **Fruit juice** stops diarrhea (3H), cures inflamed tonsils, lowers fever (1H), and reduces leg swelling (4H). Hot lemon juice induces vomit, while cold lemon juice is digestive (4H), it is applied on wounds (1H), abrasions, and contusions (1H), to reduce pain and swelling. Half lemon juice mixed with boiling water and honey prevents cold (1H), and relieves stomachache (1H). **Decoction** of lemon, rosemary, and sage leaves is used against gastritis (3H), this decoction can be added with couch grass (1H). **Lemon peel** with warm water and sugar is useful against nausea (2H). **Peel decoction** together with juice and sugar is useful in case of stomachache and intestinal pain (4H). Lemon peel **infusion** cures stomachache (2H) and indigestion (1H). Infusion of couch grass and lavender together with a lemon slice is useful in case of arthritis (3H). A massage with **lemon slice** on temple cures headache (3H) *COSM:* **Lemon juice** is used as aftershave (1H), or rubbed on the scalp against dandruff (3H)
*Clematis vitalba* L. BOLO0053711	Vitalba Clematide Vidêrba Vizzadri Asparago dei poveri Videipar Videlba Videbal Vizeibra	Wild-native	*FOOD*: **Shoots** are eaten in several dishes raw or boiled (3P, 14H, 12 M), they promote digestion (2 M). The young **shoots** are eaten in salads but only the young ones, otherwise they are irritating (3 M, 1H). **Leaves** are eaten also because they are remineralizing (1 M) *MED:* **Leaves** are used to make a diuretic **infusion** (1H, 1 M), or to make **bandages** in case of arthritis (1 M). Leaves **macerated in oil** are used to treat scabies (1H). Leaves are used in **wrap** in case of arthritis, contusion and neuralgia (1H) *SMR*: Eating **shoots** on the first of May is believed effective in keeping mosquitos away (1H) *GAME:* **Plant shoots** are used as lianas by children (1 M) *CRAFT*: **Branches** are used to make cribs (1H) *OUI:* **Dry drums and bark** (2 M, 1H), **stem** (about 10 cm long) (1 M) and **dry rolled leaves** (3 M) were smoked as cigarettes substituted. This **plant** is also called “beggars’ plant” since the beggars used it to irritate their skin and arouse more compassion in the passers-by (2H)
*Clinopodium nepeta* (L.) Kuntze BOLO0053718	Mentuccia Nepetella	Wild-native	*FOOD*: omelets are prepared with **chopped leaves**, eggs and milk (1 M). Leaves are used to flavor meat (3H) and tomatoes (1H) *MED:* **Aerial part decoction** relieves stomachache, heartburn and stomach acidity (1 M). **Leaf infusion** is digestive (2H)
*Coffea arabica* L. BOLO0014844	Caffè	Purchased product	*MED:* **Seeds** are chewed against halitosis (2H) *COSM:* **Seeds** are chewed to whiten and strengthen teeth (1H)
*Colutea arborescens* L. BOLO0046870	Colutea Stærlin	Wild-native	*MED:* **Leaf infusion** is used to treat constipation (1 M) *DOM:* **Flexible branches** are used as ties (1 M)
*Convallaria majalis* L. BOLO0006635	Mughetto Læli—Mugàtt	Wild-native	*MED:* **Leaf and flower infusion** is relaxing (6H) *SMR*: **Flower** perfume strengths memory (1H) *COSM:* **Flower macerate** is used to prepare a perfume (1H) *TOXIC:* **Fruits** are poisonous (2H)
*Convolvulus arvensis* L. BOLO0003321	Vilucchio Vlòch	Wild-native	*FOOD*: **Shoots** are used in cookery to prepare several dishes (1P)
*Coriandrum sativum* L. BOLO0006798	Coriandolo Curiandol	Wild-alien	*FOOD:* **Leaves** are used in cookery (2H) *MED:* **Leaf infusion** relieves toothache (2H)
*Cornus mas* L. BOLO0053717	Corniolo Pcôren Curniòl Cornio	Wild-native	*FOOD:* **Fruits** are eaten (6H, 2 M) to reduce sore throat (1H). Fruits are used to prepare a jam, which is astringent (1H), and alcoholic beverages such as “grappa” (4H). The **roasted nut** is used to flavor coffee (1 M) *MED:* **Bark decoction** is used against colds (5H), as antipyretic (1 M), and anti-diarrheal (1 M). **Fruits** are astringent (1H) *COSM:* **Water** from **wood decoction** is used in **compress** for oily skin (1 M) *CRAFT:* **Young shoots** are used to make baskets (1 M), **wood** is used to make pipes (1H), and, due to its strength, it is widely used in constructions (1 M) *DOM:* **Macerated wood water** is used to dye fabrics (1H)
*Cornus sanguinea* L. BOLO0003076	Sanguinella Sanguènela Sangunèla	Wild-native	*MED:* **Small branches** are put on gums in case of pain, bleeding (1H), and toothache (2H)
*Corylus avellana* L. BOLO0053684	Nocciolo Clôr Clur Clûr	Wild-native	*FOOD:* **Fruits** are used in cookery (8H) and, eaten in large quantities after the meals. They promote the intestinal physiological functions (2 M), and are used to prepare jams (1H). Nuts are eaten as energizer (2H), especially useful for outdoor workers to endure the cold (7 M) *MED:* **Leaves** are lenitive for skin (3H), they are useful against hemorrhoids and varicose veins, by improving the blood circulation (2H). **Leaf infusion** purifies the body (5H, 2 M), promotes wound healing (1H, 1 M), and is anti-inflammatory (3 M). **Leaf and bark decoction** improves the blood circulation of the eye (1 M). **Bud** decoction is used as anti-obesity (1 M) *DOM:* Collecting juniper berries in a sack, a **twig** of *C. avellana* is placed at the mouth of the sack to keep it open (1 M)
*Crataegus laevigata* (Poir.) DC. BOLO0049263	Biancospino Spinbianco	Wild-native	*FOOD:* **Flowers** are eaten by children (1 M) *MED***: Flowers** harvested in April and kept in paper bags, are prepared in **infusion** to promote sleep (3 M). Flower infusions have beneficial properties on heart and blood pressure (1 M). **Flowers**, before blossoming, are used to prepare a **hydroalcoholic extract**, which is administered in drops as a sedative, to promote sleep, and to regulate heartbeats (4 M). **Berries** infusion is beneficial for the heart (1 M)
*Crataegus monogyna* Jacq. BOLO0053714	Biancospino Spin bienc Spéin bianc Mirandal Spẹṅ bianc Bianc(-e-) spén Spén bianc Maruga bianca	Wild-native	*FOOD:* **Fruits** have refreshing properties (3H) *GAME:* **Fruits** are used by children as ammunition for the blowgun (2H)
*Crataegus* spp. -	Biancospino Spenbianc	Wild-native	*FOOD:* **Fruits** are eaten by kids for their sweetness, and are useful to stop diarrhea (1 M) *MED:* **Fruit decoction** is astringent (2H). **Fruit and flower infusion** induce sleep (6H), it is used to combat leg swelling, calm heartbeat and reduce blood pressure (2H). The **juice** made from **boiled fruits** is useful for sore throat (2H). **Flower infusion** or **raw fruits** stop diarrhea (4H). **Flower infusion** is a remedy against ringing in the ears (1H), purifies the body because it is diuretic (2H), and lowers blood pressure (3H), it is antitussive (1H), sedative (4H), and regularizes heartbeat (1H). **Flower infusion** (3H, 2 M), and **flower and leaf infusion** (1H) are useful to cure insomnia. **Flower decoction** cures anxiety (1H) and tonifies the heart (1H, 1 M). **Flower macerated in wine** is drunk (two shots a day) to prevent hypertension (1H), and has sedative properties (1H) *DOM:* **Branches** were used to heat the oven before cooking the bread (1 M) *SMR:* A hawthorn **branch** on the cradle protects the baby from evil (1H)
*Crepis sancta* (L.) Babc. BOLO0053722	Radicchiella di Terrasanta Ciocapiat	Wild-alien	*FOOD:* **Basal leaves** are eaten in salad (1P)
*Crepis vesicaria* L. BOLO0053947	Radicchiella vescicosa Striccapugni	Wild-native	*FOOD*: **Leaves** are harvested after the snow season, and eaten in a salad (4P,4 M). They have a purifying effect (2P, 1 M) *MED:* **Leaf boiling water** is drunk in the morning because it thins blood (1 M)
*Crocus sativus* L. BOLO0053263	Zafferano	Purchased product	*FOOD:* **Pistils** are used in cookery (1H)
*Cucumis melo* L. -	Melone Mlon	Cultivated	*MED:* **Pulp and seeds** are eaten to calm stomachache (1H). **Pulp infusion** calms stomachache (1H)
*Cucumis sativus* L. -	Cetriolo Zidrån Zedrån Zedran	Cultivated	*FOOD:* The **fruit** is eaten in salad since it is rich in minerals and vitamins (4H), it is refreshing (5H, 2 M) and thirst-quencher (2 M) *MED*: **Pulp** is applied on insect bites (1H). A slice of the fruit cures skin affections (2H) *COSM:* **Juice and pulp** are used to soften the skin especially after sunbathing (5H). **Slices** of the fruit placed on the eyes are useful to deflate eye bags and swelling (4H, 3 M)
*Cucurbita maxima* Duchesne -	Zucca Zocca marenna	Cultivated	*FOOD:* It is used in cookery (3H) *MED:* **Seeds** are peeled, crunched, and mixed with sugar to treat intestinal parasitosis (1H). **Seed infusion** is vermifuge (2H) *COSM:* The **pulp** applied on the skin makes it smoother and firmer (2H)
*Cucurbita pepo* L. -	Zucca Zòca Zócca	Cultivated	*FOOD:* **Fruits** are used in cookery (4H) *MED:* **Roasted seeds** promote digestion (4H), they are diuretic (2H), and useful against intestinal worms (1H)
*Cupressus sempervirens* L. BOLO0049344	Cipresso Arzipræs	Wild-alien	*FOOD:* **Fruits** are used in cookery (2H) *MED:* **Fruit macerated in water** is useful against spasmodic cough (1H) and venous stasis (2H). The **oleolite of fruits** relaxes back muscles (2H), and is useful in case of sciatica (1H) *SMR*: The dead are buried next to the cypress to ensure “peaceful rest” (1H)
*Cyclamen hederifolium* AitonBOLO0052183	Ciclamino Neapolitanum	Wild-native	*MED:* **Bulbs** boiled **in wine** are useful against ringing in the ears (tinnitus) (2H)
*Cydonia oblonga* Mill. BOLO0001857	Melo cotogno Mail(g)dågnn	Cultivated	*FOOD:* **Fruits** are used in cookery (10H) *MED:* **The fruit** boiled in sugar is squashed and used to remove catarrh (4H)
*Cynara cardunculus* L. BOLO0055365	Cardo Chèrd Cærd Carciofo Scarciôfel Carciòfel Chèrd Carciòf (S)carciòfel Carciofen	Wild-native	*FOOD:* Artichoke is used in cookery (9H), **fresh flowers** are stored in oil (2H). **Cooked flowers** are depurative (1H), protective for the liver (1H), laxative (1 M), prevent liver diseases (1 M), and lower fever (1H). **Leaves** are used to prepare a digestive liquor (2H). **Young leaves** cooked and seasoned with oil are depurative (1 M) and detoxifying for liver (2H). **Leaves** are eaten to promote liver and pancreas secretions (4H). The **fresh pith of the stem** (called “costa”) with lemon juice is eaten in salad (1 M), it is protective for liver (1H) and promotes liver functions (1H); **boiled in water** it has liver depurative activity (3H), purifies the body (3H) and protects the liver (3H). **Seed infusion** keeps the liver healthy (1P) *MED:* **Boiled leaves** promote digestion (1H) and are useful against headache (2H). The **decoction of fresh leaves** lowers cholesterol (1H, 1 M). The **decoction of roots and leaves** has diuretic properties (6H). The inner part of the **stem** has diuretic proprieties (4P). **Leaf infusion** purifies the liver(2H) *DOM:* The **boiling water** is useful to dye fabrics (1H)
*Cynodon dactylon* (L.) Pers. BOLO0049209	Gramigna Gramaggna	Wild-native	*FOOD:* In time of war, it was made a flour out of the **rhizome** (1H) *MED:* **Whole plant infusion or decoction** has diuretic properties (11H). Whole plant infusion with lavender and a lemon slice is useful against arthritis (3H). **Rhizome decoction** cures stomachache (3H), and it is used as douching for genitals to refresh from burning sensation (6H). **Leaf decoction** together with sage, rosemary, and lemon is useful against gastritis (1H). **Leaf syrup** is antitussive (1H) *AGROPA:* The **rhizome** is used as feed for pigs (1P, 3H)
*Cytisus scoparius* subsp. *scoparius* BOLO0047328	Ginestra dei carbonai Scornabec	Wild-native	*MED:* limbs are immersed in **whole plant infusion** to treat rheumatisms (1 M). **Compress of flower buds** is used to treat abscesses (1 M) *DOM:* **Branches** are used as ties (1H, 1 M)
*Cytisus* spp. -	Ginestra	Wild-native	*MED:* **Infusion of flowers,** harvested in spring–summer, drunk two or three times a day, is diuretic (2 M), sedative (1 M), laxative (1 M), and prevents heart conditions (1 M). **Dry flower decoction** is used in case of cough (1 M) and asthma (1 M)
*Dactylis glomerata* L. BOLO0003292	Erba mazzolina Erba mazzuleina	Wild-native	*FOOD:* **Aerial parts** are eaten in salad (2H) *AGROPA:* **Aerial parts** are used to feed livestock (3H)
*Daucus carota* L. BOLO0052790	Carota selvatica Carota Arcot Arcôt Pistinèga	Wild-native	*FOOD:* **Roots** are edible (5H) *MED:* **Roots fresh or boiled** stop diarrhea (9H), and improve and preserve the sight (5H), The **green organs** of the carrot, kept in the mouth, reduce mouth ulcers in kids (2H). **Flower compress** heals burns (1 M). **Decoction of flowering tops** in **compress** calms itch and pain of insect bites (1 M). **Cataplasm of flower and leaves** is applied on pimples (1 M) *COSM:* **Roots (fresh or boiled)** promote suntan and skin regeneration (1H) *VET:* Livestock are fed with carrots in case of cough (2H)
*Delphinium consolida* (L.) BOLO0602028	Speronella Erba de grên	Wild-native	*MED:* The **decoction of the plant** has its anti-lice activity (1 M)
*Delphinium staphisagria* L. BOLO0045268	Stafisagria Êrba pr i bdûc’	Wild-native	*MED*: **Seed infusion** is used against lice and scabies (4H) *TOXIC:* **Seed infusion** is poisonous if drunk (2H)
*Diospyros kaki* L.f. BOLO0011460	Caco Kako Cachi	Cultivated	*MED:* **Leaf infusion** relieves sore throat (1H). Unripe **fruits** are astringent (1H) *COSM:* **Fruit pulp** is applied on the skin to make it smoother and softer (2H)
*Diplotaxis tenuifolia* (L.) DC. BOLO0053700	Rughetta selvatica Rocla	Wild-native	*FOOD:* **Fresh leaves** are eaten in salad (6H)
*Dipsacus fullonum* L. BOLO0003288	Cardo dei lanaioli Erba di brec Sgærza	Wild-native	*AGROPA:* It is eaten by mules (1 M)
*Dipsacus laciniatus* L. BOLO0015328	Cardo dei lanaioli Sgærz	Wild-alien	*FOOD:* **Leaf decoction** is drunk as a purifying agent (1H) *DOM:* **Bunches of flowers** are used for wool carding (1H)
*Dryopteris filix-mas* (L.) Schott BOLO0052407	Felce maschio Félvsa	Wild-native	*MED:* **Root decoction** is a remedy against intestinal parasites (1 M) *SMR*: The pillow filled with **leaves** relieves legs and feet pain (1 M)
*Echinacea angustifolia* DC. BOLO0602032	Echinacea	Cultivated	*MED:* **Root decoction** cures herpes simplex (3H), and prevents flu (3H)
*Echium vulgare* L.BOLO0004948	Erba viperina Echio Êrba plåuʃa	Wild-native	*MED:* **Root decoction** is an antidot against snake bites (1 M). **Leaf juice** is placed directly on the viper bites (3 M) *SMR*: Drinking **the decoction of the root** not only is an antidot against snake bites, but it also able to prevent this from happening (1 M)
*Elymus repens* subsp. *repens* BOLO0042714	Gramigna Gramègna Gramàgna Mulaccia Gramæggna	Wild-native	*FOOD:* **Root decoction** is purifying (2P, 2H), and refreshing for the intestine (2H). **Whole plant** is purifying (1H) *MED:* The **whole plant,** rubbed on the skin, quenches the itching due to nettle sting (1P). Whole plant **infusion** is diuretic (2H), and for this reason, is a remedy for urinary tract infections (1H). **Root boiling water** is anti-anemic (1 M). Root **decoction** is diuretic (1P, 4H), and laxative (1P), it is a remedy for intestinal and bladder inflammation (1H) *AGROPA:* It is a fodder plant (1H)
*Equisetum arvense* L.BOLO0052396	Equiseto Erba cavallina Coda cavallina Stupion Covva ed caval Cuzédra Coda d’caval Cô d cavâl	Wild-native	*FOOD:* **Shoots** are used in cookery (2H) since they are rich in mineral salts (4H). Fertile **branch** is cooked like asparagus fruits (1H). **Dry leaf infusion** is diuretic, detoxifying (2 M), and excellent remineralizing (1 M). **Whole plant infusion** is purifying (1H), it is diuretic and remineralizing (2H). **Whole plant decoction** is remineralizing (2H) *MED:* **Stem decoction** is a remedy against flu (1P). The stems are used to dab hemorrhage due to wounds (2P). **Shoot infusion** is diuretic (7H), it is used for burning sensation of intimate areas (2H), and it cures canker sores in children (1 M). **Whole plant decoction** is diuretic (1H) and digestive (1H). **Branch decoction** is diuretic and is used against cystitis (4 M). **Leaf decoction** cures kidney stones (1 M). **Leaf infusion**, drunk before meals, relieves arthritis pain (1 M), and is a remedy for osteoporosis (1 M). An anti-rheumatic **ointment** is made with **grounded and boiled leaf and stem** (3 M) *DOM:* Dried **aerial parts** are used to sand the wood and to polish the pots (1P) *AGROPA*: It is a pest plant of vegetable gardens (1 M). **Whole plant** is cultivated next to tomato because it protects it from diseases (1H) *REP*: **Boiled leaves** together with nettle leaves are an excellent pesticide (1 M)
*Equisetum telmateia* Ehrh. BOLO0052392	Equiseto Coda cavallina Coda cavalèna Cô d’cavâl	Wild-native	*FOOD:* **Whole plant** is rich in mineral salts (1 M). **Branch infusion** is purifying (1H) *MED:* **Aerial part infusion** strengthens kid bones and reduces the incidence of bone fractures (2H). **Leaf juice** is drunk in wine to stop diarrhea (1H). Cotton soaked in **leaf juice** is inserted in the nose to stop bleeding (1H). **Fronds** are chopped and the **poultice** is placed on bleeding wounds to stop hemorrhage and to promote healing (1H). **Branch infusion** is diuretic (2H, 2 M), and purifying (1H). **Decoction of sterile stems** is a good diuretic (1H) and antitussive (2H) *COSM:* **Aerial part infusion** strengthens the hair and helps to manage alopecia (2H)
*Erigeron canadensis* L. BOLO0052437	Erigero Sæpla	Wild-native	*FOOD*: **Leaves and stems** are placed, together with other plants, in a container done with the bladder of ruminants (called "pitarola"), for the preparation of a cream used as rennet (1 M)
*Eruca vesicaria* (L.) Cav. BOLO0009292	Rucola Ruchetta Róccla Rocla Rugaͤtta	Wild-native	*FOOD:* **Leaves** are eaten in salad (7P, 13H, 2 M) or used to flavor dishes (3H). **Leaves** stimulate appetite (1 M) *MED:* **Aerial part decoction** promotes sleep (1P). **Leaves** sedate cough (1H), aid digestion (1H,2 M), and strengthen memory (1H,1 M)
*Euonymus europaeus* L. BOLO0003052	Berretta da prete Caurôs	Wild-native	*MED:* **Fruit macerate** is antiparasitic (1H) *CRAFT:* **Branches** are used to make brooms and toothpicks (1H)
*Eupatorium cannabinum* L.BOLO0003278	Eupatorio Chenva	Wild-native	*MED:* **Root decoction** is a remedy for constipation (1 M). **Fresh leaves** are wound and sore healer (1 M)
*Euphorbia cyparissias* L. BOLO0002910	Euforbia Euforbia cipressina Erba de lat Erba latarola	Wild-native	*MED:* The **latex** contained in the **stem** is a remedy for warts and leeks (2 M, 1H)
*Euphorbia helioscopia* subsp. *helioscopia* BOLO0002904	Euforbia Calenzuola Êrba dal vulâdg Êrba däl vulâdg	Wild-native	*MED:* **Leaf infusion** lowers temperature (2H). **Root decoction or seed oil** is used to expel intestinal parasites (2H). The l**atex** of the **stem** removes warts and calluses (2H, 1 M) *TOXIC*: The **latex** from the **stem** is very poisonous if eaten (1H)
*Euphorbia lathyris* L. BOLO0052732	Euforbia	Wild-alien	*REP*: It is cultivated to keep moles away since it produces toxic **latex** (2H)
*Euphorbia* spp.	Euforbia	Wild-native	*AGROPA:* Rabbits are fed with the **whole plant** (1H), *DOM*: **Leaf cooking water** is used to color eggs of green (1H)
*Euphrasia officinalis* L. BOLO0054007	Eufrasia Èrba pr'i och Eufræsia	Wild-native	*MED:* **Whole plant infusion** is used to wash redden eyes and in case of conjunctivitis (2H)
*Fagus sylvatica* L. BOLO0053682	Faggio	Wild-native	*FOOD:* The **fruits,** deprived of their outer shell, are eaten as hazelnuts (1 M) or roasted and used as an alternative to coffee (2 M). Shepherds used to eat young **leaves** as hunger quencher (1 M) *MED:*** Bark decoctions** is antipyretic (2 M). From the **charcoal of the wood** is obtained a balsamic substance, which is used for **fumigations** in case of cold (1 M) *CRAFT:* The **wood** is very resistant and it was often used to craft furniture (4 M) and musical instruments (1 M) *DOM*: **The wood** is used as fuel for the fireplace (1 M)
*Ficus carica* L. BOLO0055353	Fico Fîg Fic Fîg Fîg fiurån	Wild-native	*FOOD:* **Figs** are prepared in jam (3H), that is laxative (2H, 5 M). Figs are eaten fresh (2 M, 1H) or dried since they are laxative (19H) and rich in mineral (10H). They are used to prepare a traditional Christmas cake, made with apple mustard, pine nuts, and dried grapes (1 M) *MED:* Dried **figs** are eaten to treat sore throat (1H). Fresh or dried fruit cures catarrh (4H). Fresh fruits are a remedy for colitis and vomiting (1H). **Fruit decoction in milk** is useful against colds and cough (1H). **Fruit juice** is applied on calluses (1 M) and warts (1 M). A **liquor** is obtained with **fruits**, yeast, sugar, and water and after maceration it is used as laxative (1 M), antitussive (1 M), for sore throat (1 M) and intestinal pain (1 M). **Fruits** are left in sugar and then boiled with water to obtain “fichi sciroppati”, the resulting **syrup** is used as cough sedative (1 M). The white **latex** from **leaves** and **fruits** is used to remove calluses and warts (5P, 28H, 10 M). It is applied on pimples (1H) and on insect bites (2 M), and is wound healer (1 M) *DOM:* **Leaves** are abrasive and are used in cleaning (1P)
*Filipendula ulmaria* (L.) Maxim. BOLO0034718	Spirea ulmaria	Wild-native	*MED:* **Aerial part infusion** cures gout (1H), and prevents flu (1H) *COSM:* **A cream** made with **aerial part** is a remedy against cellulitis (1H)
*Foeniculum vulgare* Mill. BOLO0055391	Finocchio selvatico Fnòcc Fnòch Fnocc sambadg Fnôć Fnòc'	Wild-native	FOOD: **Aerial part** is edible (1P, 5H). Fennel is used to prepare biscuits (2H). **Fruits and stems** are eaten in salad (3H). **Leaves** are eaten in salad or used to aromatize dishes (1H). **Seeds** are used to prepare aromatized “salami” (1 M) and “zucarén” (traditional biscuits prepared for marriages) (6H). Seed **macerate in wine** stops hiccup (1H). **Aerial part macerate** is used to prepare a digestive “grappa” (1H). **Fruits** are eaten to promote digestion (6H) and to reduce vomiting during pregnancy (2H). **Stems with young buds** are used to prepare a digestive and sweet brandy (1H) *MED:* **Whole plant infusion** is digestive (11H). **Seed decoction** in **wraps** is a remedy for conjunctivitis (3H). Seed **infusion** is digestive (1H), deflating (1H) and together to licorice roots is galactagogue (3H). **Fruit infusion** is used in wraps on inflamed eyes (1H), it is digestive (2 M) and deflating (1 M), and it is a remedy for gastro-intestinal issues (1 M). **Fruit decoction** is galactagogue (1H). **Infusion** of fennel, anise, and parley fights stomach acidity (3H). **Leaves** are used to **wrap** abscesses (1H). **Leaf infusion** (2P) and **decoction** (2P) are digestive. **Root infusion** reduces intestinal gases (1H). **Root syrup** made with parsley, celery, and butcher’s broom reduces intestinal gases (6H)
*Fragaria vesca* L. BOO0055389	Fragoline di bosco Frœvla Frèvla	Wild-native	*FOOD:* **Fruits** are used in cookery (4H), to prepare jams (1H) *MED:* **Whole plant macerate** stops diarrhea (2H). **Rhizome decoction** is used to wash the oral cavity in case of sore throat (1H). **Leaf juice** is used to remove red dots on the skin (1H). **Dried leaf infusion** is purifying (1 M). **Root decoction** and **raw fruits** have purifying properties (1H) *COSM:* **Fruit juice** mixed with milk and yogurt tones the skin (1H)
*Fragaria viridis* Weston BOLO0034670	Fragola di bosco Frèvla	Wild-native	*FOOD:* **Fruits** are used in cookery to prepare sweets and cakes, jams, and syrups (5H). Fresh fruits topped with red wine or lemon and sugar are eaten since they are rich in nutrients (3 M), help in case of flu (1 M), lower blood pressure (2 M), and have anti-inflammatory properties (1 M) *MED:* **Leaves** are **boiled** and drunk in case of diarrhea (2H). **Leaf infusion** is diuretic (2 M), astringent (1 M) and it lowers blood pressure (2 M). **Root decoction** cures sore throat (2H). **Slices of fruit** applied on the face cure acne (1 M) *COSM:* **Slices of fruit** are tonic for the face (1 M)
*Frangula alnus* Mill. BOLO0003053	Frangola Spen Zarven Salvâdg	Wild-native	*MED:* **Bark infusion** (with leaves from plum tree and dog rose) is useful against constipation (3H)
*Fraxinus excelsior* subsp. *excelsior* BOLO0052224	Frassino Frâsen	Wild-native	*MED:* **Bark decoction** is drunk in case of fever (1H,1 M), and diarrhea (1 M). **Leaves infusion** has laxative (2 M), and diuretic (1 M) properties *VET:* **Wood macerate** is given to drink by livestock to treat gastrointestinal disorders (1H) *CRAFT:* **Wood** was used to make handles of working tools (1 M)
*Fraxinus ornus* subsp. *ornus* BOLO0052247	Frassino	Wild-native	*MED:* **Bark decoction** relieves sore throat (6H) *REP:* Rinsing oneself with **bark decoction** keeps insects away (1H)
*Fraxinus* spp. -	Frassino	Wild-native	*VET:* The **bark** has veterinary uses (3H), the **extract in water** (having a light blue color) is used to treat sore troth in chicken (1H) REP: The **bark in water** is used to keep insects away from livestock (1H)
*Fumaria officinalis* L. BOLO0048716	Fumaria Fumæria	Wild-native	*FOOD:* **Leaf infusion** with honey purifies blood from toxins (2H)
*Galanthus nivalis* L. BOLO0046895	Bucaneve	Wild-native	*MED:* **Whole plant infusion** is an emetic that relieves gastric pain (1H), and in small quantity it helps to focus (1H)
*Galega officinalis* L. BOLO0049465	Galega Galâiga	Wild-alien	*MED:* **Fruit decoction** is a galactagogue (1H). **Leaf and flower infusion** lowers glycemia (1 M)
*Galium odoratum* Scop. BOLO0027161	Asperula Stellina odorosa	Wild-native	*FOOD:* **Fresh branches** are used to make a digestive liquor (1H) *MED:* **Whole plant infusion** purifies the liver (1H) and it has diuretic properties (1H)
*Galium sylvaticum* L. BOLO0006790	Caglio di bosco	Wild-native	*FOOD:* It was used to curdle milk (1 M) *MED:* **The whey** of the milk curdled with this plant is drunk for its anti-inflammatory activity on the urinary tract (1 M) or used externally for baths and packs (1 M)
*Galium verum* L.BOLO0004410	Gallio Impresa-gâj	Wild-native	*FOOD:* **Stems** are used to make rennet for cheese production (1H, 1 M)
*Genista tinctoria* L. BOLO0004411	Ginestra	Wild-native	*CRAFT:* The **stems,** which are very resistant, are harvested in spring and kept together in bunches stored in damp jute sacks. Because of their strength, they are used to tie up vines, and in the stuffing of chairs (2 M)
*Gentiana lutea* L.BOLO0053596	Genziana Genzîæna	Wild-native	*FOOD:* **Roots** is macerated in “grappa” with sugar for 2 months, obtaining the so called “grappa di vipera”, named after the shape of the root that looks like a viper (2 M). **Flowers and leaves** are used to make liquors (1 M), which are bitter and digestive (2 M) *MED:* **Flowers and leaves infusion** is antipyretic (1 M), immune system stimulant (2 M), digestive (3 M), astringent (1 M), and vermifuge (1 M)
*Gentiana* spp. -	Genziana Genzîæna Genzianella	Wild-native	*MED:* Harvested in meadows, **root boiling water** is a powerful laxative (1 M). **Root infusion** is useful for gastrointestinal disorders (1 M). **Root decoction** is beneficial for the liver (2 M). **Leaf boiling water** is beneficial for the intestine (1 M) *DOM:* **Flowers** is used to dye fabrics (1 M)
*Geranium robertianum* L. BOLO0052405	Erba Roberta Erba rossa Geràni seivâdg	Wild-native	*MED:* **Whole plant decoction** is used to lubricate eyes (1 M)
*Ginkgo biloba* L. BOLO0015151	Ginkgo	Cultivated	*MED:* **Leaf infusion** helps to keep the memory in a good state, preventing brain diseases if it is taken once a day in the evening (2H). It also reduces headache (2H) and prevents ictus (1H). **Leaves** are used to prepare a **cream** useful to heal creaked skin (1H) and to relieve hemorrhoid pain (1H)
*Glechoma hederacea* L. BOLO0048777	Edera terrestre Erba quattrina Laͤddra terræstra	Wild-native	*MED:* **Leaves** have cicatrizing and lenitive proprieties (1H). **Leaf juice in lard** has cicatrizing properties useful in case of burns and wounds (1H). **Whole plant boiled in milk** is used to treat bronchitis (1 M)
*Globularia bisnagarica* L. BOLO0003041	Morine	Wild-native	*MED:* **Leaf decoction** stimulates diuresis (1H) and intestinal transit (1H)
*Glycyrrhiza glabra* L. BOLO0055352	Liquerizia Nigulezzia Miclézzia Sugabacàtt	Cultivated	*FOOD:* **Roots** are eaten (6H). **Juice** is used to prepare candies (8H) *MED:* **Dried roots** are chewed to raise the blood pressure (13H) and to remove catarrh (9H), in addition, they are laxative (3H). **Root decoction** relieves throat inflammation (3H) and cough (9H), is laxative (2H) and digestive (3H). Root **infusion** is diuretic (2H) and laxative (2H). **Green root infusion** together with fennel fruits is galactagogue (3H)
*Hedera helix* L. BOLO0049243	Edera Læddra Ladra Laͤddra Laddra	Wild-native	*MED:* **Fresh leaves** in **cataplasms** heal pimples (1H). Leaves in cataplasm soften the skin, heal cradle cap (6H), and cure varicose veins (1 M). A cataplasm made of leaves and lard is applied on burns (1H). **Cataplasms** of **boiled** leaves heal pimples (1 M). Leaves are used to prepare an **ointment** that promotes wound healing (4H). **Leaf boiling water** is used for footbaths useful in case of ingrown toenails (1 M). Leaf **infusion** is a remedy for flu (2H), cough (1H), sore throat (4H), hemorrhoidal (5H) and menstrual pain (2H), caries, and toothache (1H). **Leaf decoction** regularized menstrual period (1H), it is used externally to make **bandages** for rheumatism (2 M) and neuralgia (1 M), and used to make healing **packs** for pimples, redness, and sores (1 M). Leaf **decoction in vinegar** is used to wrap painful joints and in case neuralgia (2H). **Decoction** of **young fresh leaves in compress** relieves calluses (1 M). **Fumigation** made with boiling **stems and leaves** is a remedy for cold (1 M) *COSM:* **Leaf boiling water** is used for the last rinse on dark hair to give shine (1 M). **Fresh leaf juice** is used to dye hair (1H). **Leaf infusion** is anti-cellulitis (1H) SMR: **A leaf** is placed on the hair to prevent the blisters formation after getting burned (1H) *VET:* When goats are nervous they are fed with **fresh leaves** since it is relaxing (2H) *OUI:* At the beginning of 1900, asylum patient heads were wrapped with ivy leaves, believed able to make them peaceful (1H)
*Helianthus tuberosus* L. BOLO0052419	Topinambur	Wild-alien	*FOOD:* The **rhizome** is boiled and eaten (2P, 1H) and has diuretic proprieties (5H). It is used to prepare several dishes (4H) and is rich in mannitol (1P)
*Helichrysum italicum* (Roth) G.Don BOLO0007211	Elicriso Perpetuino	Wild-native	*MED:* **Flower infusion** has a cough sedative effect (1P). **Aerial part decoction** is useful to disinfect the first respiratory tract (4H), and for heartburns (2H), or it is used as a **rinse** to disinfect the throat (2H) *COSM:* **Leaves** are used to prepare an **ointment** that reduces cellulitis (1H)
*Helichrysum stoechas* (L.) Moench BOLO0053442	Stecade Liquérézia	Cultivated	*MED:* It is antiasthma and is used to treat cold (2 M). The **decoction** is liver depurative (1 M). **Flowering top infusion** is used for rheumatism (1 M)
*Helleborus foetidus* L. BOLO0052535	Elleboro Erba zitona Cava denti Êrba dal mæl zitån	Wild-native	*OUI:* The **rhizome**, positioned between the tooth and the gum, was used for the extraction of teeth, hence its vernacular name “cava denti” which means “teeth remover” (2 M) *VET:* It is painkiller and antipyretic for animals. **The root**, dried and decorticated, is inserted into a hole-incision in the ear of the animal, to cure the fever. However, it created also a large inflammation in the treated area (1 M)
*Helleborus* spp. -	Elleboro Erba de mèl ‘d l’azton	Wild-native	*VET:* The **root** is inserted into a hole-incision in the ear of the animal to treat the erysipelas, called by the informants “mèl ‘d l’azton”(2H)
*Helleborus viridis* L. BOLO0003022	Elleboro Erba nocca Erba zitona Èrba dal mèl zitòn o fitòn Êrba dal mæl zitån	Wild-native	*VET:* it is detoxifying for cattle (5 M). **Fresh leaves, roots and stems** were inserted in the ears of pigs to cure the erysipelas, removing the infection (14H, 8 M). The **root** is inserted in the ears or in the anus of cows and pigs when they do not produce milk, or they are inappetent (10H)
*Hepatica nobilis* Schreb. BOLO0057770	Epatica Fegatella Erba di bogn	Cultivated	*MED:* **Leaf decoction** is purifying for the liver (1 M). **Leaves juice** is used on pimples (1 M)
*Hibiscus* spp. -	Ibisco	Cultivated	*MED:* **Leaf infusion** reduces kidney issues, since it promotes diuresis (3H), and it is also sedative (3H). **Root decoction** reduces cough (1H), and it is used to relieve hepatic inflammation (1H) *COSM:* **Flowers** are used to prepare a shampoo to strengthen the hair (1H)
*Hippophae rhamnoides* L. BOLO0039434	Olivello spinoso Marugo	Wild-native	*FOOD:* The yellow **fruits** have a sour taste and are harvested especially in calcareous and limestone landslides. They are used to make a **jam**, and due to their vitamin and mineral content, they are very useful to strengthen the immune system (4 M) *AGROPA:* The **berry juice** was used to smooth horses’ hair (1 M)
*Hordeum murinum* L.BOLO0052315	Orzo selvatico Spigarôla	Wild-native	*FOOD:* It is often eaten by old people because it is easy to digest (1 M), and very energetic (1 M). Eating barley strengthens the bones (1 M) and helps to prevent heart (1 M) and lung conditions (1 M), it is beneficial in case of gastritis (1 M) and helps to focus (1 M). During the war, it was greatly cultivated, roasted in the fireplace and ground to make a coffee substitute beverage (1 M) *MED:* A punch of barley is **boiled in water** which, once cooled, is used to make gargles for sore throat (3 M) and gingivitis (1 M). Barley **boiling water** is useful against enteritis (1H). **Fruit decoction** is used in compress on reddened eyes (1 M), and drunk as an anti-inflammatory (1 M)
*Hordeum vulgare* L. BOLO0042641	Orzo Urzón Ôrz	Cultivated	*FOOD:* Toasted and grounded barley is used to prepare a coffee-like drink, which is purifying for urinary tract (1 M), intestine (1 M), stomach (1 M), and digestive (1 M) *MED:* **Fruits** are used to make warm **wrap** on the chest in case of flu (2H) *VET:* **Aerial parts** promote cattle digestion (1P) *AGROPA:* **Aerial parts** are used to make the cow bed (1P) *DOM:* A yellow paper, used to wrap food, it is obtained from the **straw** (1P) *CRAFT:* The **stems** are braided to make handbags (1P)
*Humulus lupulus* L. BOLO0055369	Luppolo Loppel Loppel-Lopla	Wild-native	*FOOD:* **Female flowers** are used to make beer (6H). The **tips of shoots** are eaten (1P), and have a digestive effect (1P). **Female flower infusion** stimulates appetite (1 M) *MED*: **Female flower infusion** promotes digestion (2H) and stimulates appetite (1 M). **Flower infusion** is relaxing (5H) and increases sexual desire (1H), it helps to manage sexual desire because it contains estrogens (1H). The **beer** was used on insect bites (1 M) *SMR:* **Female flowers** are used to fill the pillow of insomnia sufferers (1P, 1 M)
*Hydrangea macrophylla* (Thunb.) Ser. BOLO0054770	Ortensia	Cultivated	*MED*: **Infusion** of cleaned **roots** is drunk at the morning as diuretic (5 M), and it is useful against meteorism (1 M). **Leaf infusion** is relaxing (1 M), and depurative (1 M) *DOM:* This plant is widely used as a decoration for house and garden (5 M)
*Hylotelephium maximum* (L.) Holub BOLO0017292	Borracina maggiore Erba della Madonna Erba di san Giovanni Fèva grâsa Erba dla Madòna Êrba grâsa Fæva Grâsa	Wild-native	*MED*: **Leaves** are used to soothe burns, remove calluses, cicatrize small wounds, and to cure “giradito” (whitlow) (17H). **Leaf pulp** is used on burns (3H), wounds and pimples (1H), and on other skin diseases (1H), for its healing properties, it is used in warps on wounds and plagues to extract pus and promote healing (3H). The **plant** combined with beeswax, olive oil, and a few sprigs of elderberry is used to make a regenerating and healing **cream** for chapped skin, especially for winter rhagades (1 M). The **latex** from leaves has a cicatrizing effect, it is applied on insect bites to relieve pain and irritation (1 M), and it is used to treat burns (1 M)
*Hyoscyamus niger* L. BOLO0029275	Giuquiamo Dente cavallino Êrba d’Santa Pulògna	Wild-native	*MED*: **Flower and leaf infusion** has relaxing properties (6H). **Whole plant macerated in oil** is used to relieve pain (3H). **Leaves** are used to make anti-asthma cigarettes (1H). **Fumes** of boiling water from **seeds** are inhaled with the mouth in case of toothache (1H)
*Hypericum perforatum* L. BOLO0053787	Erba di San Giovanni Iperico Èrba ed San Zvàn Erba ‘d San Zvàn Erba d’San Zvan Êrba d' San Zvân Êrba d’San Z’Van	Wild-native	*MED*: **Flowers** are macerated in oil, obtaining a reddish **oleolite**, which has cicatrizing properties (1 M), soothing and anti-inflammatory activity (4H, 1 M), it is useful to treat burns and skin irritations (1P, 13H, 3 M), bone pain (4H), insect bites (1 M). This oleolite is prepared with flowers in 100 mL of olive oil (or vaseline oil (1 M)), then it is left under the sun for 2 weeks, shaking it from time to time (6H). The use of this oil is not recommended before sun exposure since it might induce black spots on the skin, on the contrary, it is useful as after sun (1 M). **Flower infusion** promotes digestion (1H, 1 M), reduces menstrual pain (1H), and lowers blood pressure (2 M). **Flower decoction** has cicatrizing properties and it is used in **compress** on wounds, sores, burns, erythema (1H, 1 M). **Leaf infusion** is useful against diarrhea (2H), as expectorant and cough sedative (1H), it is anti-depressant (1 M), in particular in case of depression related to menopause (1H), is also used to relieve stress, insomnia and anxiety (5H). **Leaf macerate in hot oil** is used to treat rheumatisms (3 M), pimples, burns, and as wound healer (2 M) *SMR:* The flowering tops are harvested traditionally on the night of June 24th (the day of St. Joan), and it keeps demons and evil spirits away, for this reason, a **branch** is kept above the main entrance of the house or on the stables for protection (4H, 1 M). A flower bunch is burnt to keep demons away (1 M). A bath in a bathtub full of flower increase woman fertility if it is done on 24th of June (1H)
*Hyssopus officinalis* L.BOLO0015026	Issopo Isôp	Wild-native	*MED*: **Flower infusion** is digestive (1 M), it is used against sore throat (3H), and to expel catarrh in case of cough and bronchitis (1H, 1 M). Flowering tops infusion is used in **compress** to enhance wound-healing (1H). **Leaves** are used to **wrap** wound to promote healing (2H)
*Ilex aquifolium* L. BOLO001688	Agrifoglio Ponztôp	Wild-native	*MED*: **Dry leaf infusion** is febrifuge (1 M), diuretic (3 M), astringent (2 M), and it heals hand rheumatisms (1 M). **Leaf and fruit macerate** is useful in case of rheumatic pain (2H). The **bark** is washed and used in **infusion** to drink in case of fever (1 M), and hand rheumatism (1 M) *SMR:* A **twig** was hung at the entrance of the house to keep evil spirits away (1 M) *AGROPA:* Thanks to its thorny leaves, it is cultivated in the garden and vegetable garden to delimit and protect it from wild animals (2 M)
*Iris* spp. -	Giaggiolo Iris Îrios	Wild-alien	*MED*: **Fried leaves** are applied on body parts affected by rheumatic pain (1H)
*Juglans regia* L. BOLO0053169	Noce Nus Nûʃ Nûs	Wild-native	*FOOD:* **Fruits** are eaten (7H) for their energetic value (3 M), and to promote digestion (1 M). They relieve migraines (1 M), decrease stress (1 M), and promote small wound healing (1 M). To eat walnuts helps to stay young (1 M). With the **unripe nuts** is prepared the digestive liquor called “nocino” (7 M). Twelve walnut husks are macerated in alcohol for 48 h to obtain the “nocino” a typical digestive (5P, 26H). Walnuts are collected during the night of St. Joan (24th of June) and macerate in alcohol, sugar, and liqueur wine for 40 days, obtaining the “nocino”, which has digestive properties (2 M, 8H). **Squeezed nut oil** is used to cook (1 M). A **leaf** is put under the pan to give a nut flavor to the “tigelle” bread (1 M) *MED*: **Leaf decoction** is used in **lavender** or **compress** against *Herpes simplex* (1 M). **Leaf decoction**, **in foot bath**, improves blood circulation (1H). **Leaf infusion** is astringent (3H). **Leaf macerated in water** is used to wash genitals and to heal from genital infections (1H). **Walnut husk infusion** is used in case of renal colic (2H), and intestinal parasites (3H). Squeezed nuts oil is able to lower cholesterol although it goes rancid earlier than olive oil (1 M) *COSM:* **Walnut husk decoction** is used to give brownish color to the hair (1 M, 1H). **Squeezed nuts oil** is used to soften skin (1 M). **Leaves are boiled** to do refreshing footbath that decreases sweaty feet (2H, 1 M) *VET:* Cats and dogs are rubbed with **leaf decoction** to keep fleas and ticks away, and to treat skin inflammations (1H, 1 M) *CRAFT:* The **wood** is widely used for furniture (1 M) *DOM:* **Squeezed nuts oil** is used to light oil lamps (1 M) *SMR:* A **walnut tree** planted in proximity of the house is considered a good auspice (2H). To keep one or two **walnuts** in the pocket heals from fever (1 M). A legend says that keep a **couple of walnuts** in the pocket keeps evil spirits away (1 M). **Fresh leaves** are applied on ears to heal mumps (1H)
*Juniperus communis* L. BOLO0053710	Ginepro Żanævver Zaneivar Żanàvver Ȥanaͤvver	Wild-native	*FOOD:* **Cones** are used in cookery, to flavor the wild game and meat (2P, 20H, 5 M), and are an ingredient for ham and “salami” (1 M). They are used to make toning (1 M) liquors (11H, 14 M) *MED*: Juniper **cones** are antihemorrhagic (1 M), astringent and are used to treat pimples (1 M). **Cone decoction** has disinfectant properties for the respiratory tracts (3H), and it is used to treat urinary tract infections (1H). **Infusion of cones** is diuretic (3 M), and purifies the body (1H). **Cones macerated in withe wine** together with mustard is a remedy for cystitis and kidney stones (1 M). Cones are macerated to obtain a **syrup** useful in case of cough (1H). Cones are **boiled** to obtain a thick mixture useful against urinary incontinence (2H). **Cones ash** is inhaled in case of cold (1H). **Flowers**, harvested in spring, are used to make a purifying and slightly laxative **infusion** (1 M) *COSM*: **Leaf macerate** with lavender, rosemary, thyme and sage is used on fatty skin (2H) *DOM:* **Whole plant** is burned to perfume the house (1H). This plant is used to clean chimneys from soot (1 M) *SMR:* **Branches of this plant** were placed in the stables to keep “evil eye” away (1 M). *REP:* **Branches** keep insects away from stables (1 M) *CRAFT:* Traditionally the Christmas tree was a juniper (1 M)
*Laburnum anagyroidis* Medik BOLO0003099	Maggiociondolo Maz	Wild-native	*AGROPA:* Sheep and goats if fed with this plant produce tastier milk for cheeses (1 M) *OUI:* “Maggiociondolo” was used for the traditional flowery processions in May for its beauty and perfume (1 M)
*Lactuca sativa* L. BOLO0602029	Lattuga Insalae Latuga Latûga	Cultivated	*FOOD:* It is commonly eaten in salad (8H), because it is purifying (3H), it is used to prepare a soup that calms stomachache (1P) *MED:* **Fresh leaves** are applied on dermal inflammations for soothing (1H). Raw leaves are galactagogue (4H) as well as **leaf decoction** (2H), which is also applied on the sore tooth to calm the pain (1P), and it is used to prepare an anti-cough **syrup** (2H). The **juice** extracted from the **lettuce core** is used to calm cough in children (1P)
*Lamium amplexicaule* L. BOLO0049113	Erba ruota Sementaria	Wild-native	*FOOD:* **Leaves** were chewed because they taste like mint (1 M)
*Larix decidua* (L.) Mill. BOLO0003050	Larice	Wild-native	*MED*: **Hydroalcoholic extract** of newly **shoots**, harvested in spring, cures sore throats (1 M)
*Lathyrus oleraceus* Lam. BOLO0014393	Taccole Mangiatutto	Wild-native	*MED*: **Whole plant** is eaten since it is laxative and reduce intestinal gasses (1H)
*Laurus nobilis* L. BOLO0053683	Alloro Mlor Mlòri Mlôr	Wild-native	*FOOD:* **Dried leaves** are used to flavor dishes (4P, 19H, 4 M), and help digestion (1H). Leaves were used together with other herbs to curdle the cheese (1 M). Leaves together with lemon peel are used to prepare a liquor called “canarino” (1H). **Fruits** are used to prepare a digestive liquor called “laurino” (1 M) *MED:* **Leaves** are used to make wraps on the chest in case of cold (1H), they are smelled to relieve nausea and stomachache (1H). **Leaf decoction** is a remedy for stomachache (4P), cough (3P), cold (1P), and intestinal gases (2H), and it is diuretic (1P). Leaves decoction was given to children to make them fall asleep (1 M), the decoction with chamomile promotes relax (1P) and sleep (1P). **Leaf infusion** disinfects oral cavity (5H), it is a remedy for colds (2H), to lower blood pressure (1H, 1 M), and it has a sedative effect (1 M). Dried leaves infusion is drunk after meals to treat aerophagia (1H). Leaves infusion together with lemon peel (called “canarino") is useful against stomachache and nausea (9H). **Leaves baths** and **footbaths** stimulate blood circulation (6H). A **berry** is eaten to lower blood pressure (1 M). **Fruits** are **macerated in oil** to make **wraps** against hemorrhoids (1H) *COSM:* **Leaf infusion** is a remedy for sweaty feet (2H) *DOM:* **Dried leaves** are burnt to overtake bad smell (4H), and to perfume the house (1H) *REP:* Begs filled with **leaves** keep moths away from drawers (1H). Dried leaves are used to keep moths and small insects away from armchair (1H) and from flour of wheat and barley (1H)
*Lavandula angustifolia* subsp. *angustifolia* BOLO0029441	Lavanda Lavand(l)a Lavȧṅdla	Wild-native	*FOOD:* **Leaves** (1H) and **flowers** (1H) are used to flavor several dishes *MED:* The **essential oil** has relaxing and calming action (1P), 4–5 drops are used in fumigation to relieve flu (1 M). **Flowers** are used for **fumigation** (placed in boiling water) balsamic for the first respiratory tract (2H). **Flower alcoholate** is used for messages to relieve headache (1H). **Flower alcohol macerate** has disinfectant properties for the skin (1H). **Flower infusion and essential oil** calm pain (7H), and is anti-lice (5H). **Flower infusion** relaxes muscles (2H), is used to treat migraine (1 M) is sedative (2H, 2 M) and diuretic (1 M), it is used to make decongestant **fumigation** (3 M). It can be used topically or drunk to purify the skin, especially in case of acne (1H). **Topically**, flower infusion is used to promote scar healing (2 M), to make massages that relax muscles (1 M), and bandages for burns (1 M). Lavender infusion together with lemon peel and couch grass is useful in case of arthritis (3H). Lavender infusion together with violet, sage and chamomile is a remedy for arthritis (1H). **Top flowering decoction** treats cold (1 M). Some drops of **essential oil** on the pillow conciliate sleep (1 M) *COSM:* **Leaf macerated** with juniper, rosemary, thyme and sage is used on fatty skin (2H). **Flowers** are used to make perfumed and relaxing baths (1H). Flower **infusion** is used to make footbaths (1 M). Flower **essential oil** hydrates the skin (4P) and the scalp (3P). Flower **macerate** is used as perfume (1H) called “Acqua di lavanda” *VET:* In case a dog is bitten by a viper, **flowers** are rubbed on the bite (1H) *REP:* Bags filled with **flowers** are placed in the wardrobe to keep away insects such as moths (2P, 20H, 1 M). A sprig is kept in the closet as a pest repellent for clothes (2 M). **Aerial parts** are rubbed on the skin to keep insects away (1P, 5H). Some drops of essential oil in the house keep scorpions away (1H). **Flower alcohol macerate** applied on animal skin repels insects (1H) *DOM:* Bags filled with **flowers** are used to perfume linen (10P, 28H, 4 M) and are used to make scented soaps (1 M). **Essential oil** is used also to perfume the house (2P) and in scented diffusers (1 M). The plant is ornamental (3 M). Often the **flowers** are placed in jars to perfume and refresh the room (1 M) *SMR*: The **flowers** are harvested on the night of S. Johan (23th of June) and placed in small bags under the pillow to induce premonitory dreams (1 M). Bunches of lavender are kept in the house to bring calm, thanks to the color and scent (2 M) *AGROPA:* The **whole plant** attracts bees which make honey (4H) *OUI*: The **essential oil** is used for relaxing massages (1 M). **Lavender bath wash** is made to cure kid weakness (1H)
*Lavandula* spp. -	Lavanda Lavand(l)a	Wild-native	*MED:* **Dried flower infusion** relieves abdominal muscle spasms (1 M), fatigue, and exhaustion (1 M). **Flower cream** relieves abdominal pain (1 M) *SMR:* A pillow filled with lavender **flowers** helps to calm headache (1P) *DOM:* A **bunch of flowers** is used to perfume the linen (3P) *REP:* A **bunch of flowers** keep insects away (1P, 2 M)
*Leopoldia comosa* (L.) Parl. BOLO0046847	Lampascioni Cipollaccio col fiocco Purraͤtt	Wild-native	*FOOD:* **Bulb** is used in cookery (1H), it is eaten in salad for its diuretic activity (1H, 1 M) *MED:* **Bulb juice** is rubbed on insect bites to soothe the itch (1 M)
*Leucanthemum vulgare* subsp. *vulgare* BOLO0046847	Margherita	Wild-native	*FOOD:* **Flowers** are eaten in salad (1P)
*Levisticum officinale* W.D.J. Koch BOLO0015233	Levistico	Cultivated	*MED:* **Aerial parts infusion** purifies the liver (2H)
*Lilium candidum* L. -	Giglio Giglio di Sant’Antonio	Cultivated	*MED:* **Pistil macerate** (in alcohol for 15 days) is massaged to relieve rheumatic pain (1H)
*Linaria vulgaris* subsp. *vulgaris* BOLO0003308	Linaria *Êrba däl stréjj*	Wild-native	*FOOD:* **Flowers** are eaten in salad (1H) *MED:* **Leaf and seed infusion** is purifying (3H). **Aerial parts** in **cataplasms** cure skin conditions (3H) and hemorrhoids (3H) *SMR:* The plant is used by wizards to perform evil deeds (1H)
*Linum usitatissimum* L. -	Lino Lén Lein Len Lẹṅ	Purchased product	*MED:* **Seed flour in cataplasm**, is used to cure furuncles (2P), wound infections (4H), and abscesses of insect bites (2P); applied on the thorax, cures cough (5P, 1H), sore throat (1P) and bronchitis (1P), by enhancing catarrh expulsion (5P, 3H). Seed flour is used in **warm wraps** as a remedy for cold (24H), stomachache (8H), muscular inflammation (1H), and, applied on the belly, cures constipation (2H). **Boiled seeds wrapped** in a piece of fabric and placed on the chest is cough sedative (14H). A **paste** obtained by mixing **seeds with water**, is put on a **gauze** applied on the chest as a remedy against cough (3 M), pneumonia, and bronchitis (11H). Two teaspoons of **seeds** are left in a glass of water overnight, then the water is drunk for its laxative effect (15H, 1 M). **Seed infusion** is anti-inflammatory (1 M), laxative (1 M), stimulating of immune system (1 M) and cures cystitis (1 M). **Leaves decoction** is used **in wrap** on the belly in case of flu (1H). **Leaves in cataplasm** are applied on the belly in case of cough (2H), or on ankles in case of sprain (2H) *COSM:*** Seed infusion** is applied on damaged hair (2 M), and is emollient for the skin (1 M) *DOM:* **Seed oil** is used to shine wood furniture (1H)
*Lonicera caprifolium* L. BOLO0053681	Caprifoglio Ligabòsc	Wild-native	*MED:* **Bark decoction** stimulates sweating (2H). **Leaf infusion** is anti-inflammatory for the throat (2H). **Leaves** are applied on the skin to cure wounds and vesicles (2H). **Fruit juice** is laxative (7H) *TOXIC:* **Raw seeds** are poisonous (7H) *COSM:* **Flowers** are used to prepare beauty creams and perfumes (7H)
*Lonicera periclymenum* L. BOLO0602030	Caprifoglio	Wild-native	*FOOD:* **Flowers** are sucked for their sweet taste (1 M) *MED:* **Flower infusion** is diuretic (1 M), and relieves belly pain (1 M)
*Lotus corniculatus* L. BOLO0046868	Ginestrino comune Trifoi zal Orioleina Gatell	Wild-native	*MED:* **Flower infusion** is used in **compress** on eczema (1H) *AGROPA:* The **whole plant** does not cause flatulence; thus, it is good as feed for livestock (1H)
*Lupinus albus* subsp. *albus* BOLO0009685	Lupino Lupino	Wild-alien	*MED:* **Seed cooking water** is anti-lice (2H). **Seed macerate** is used on eczema and to remove cradle cap (1H). **Leaf infusion** is a remedy for stomach acidity (1H)
*Malus domestica* (Suckow) Borkh. -	Melo Mæil Mail	Cultivated	*FOOD*: A digestive liquor is prepared with the **seeds** (1H). **Cooked apples** are laxative (2P) *MED:* **Root bark decoction** is a remedy for the fever (1P). **Bark decoction** stops diarrhea (1H). **Juice** obtained from cooked apple cures cough and bronchitis (9H). **Flower infusion** is drunk in case of cough (3H), and sore throat (3H). **Fruits decoction** is vitaminizing (1H)
*Malva sylvestris* L. BOLO0046878	Malva Mèlva Mæiva Méiva Mælva	Wild-native	*FOOD*: **Leaves** are used to prepare a liquor (3H), which heals from stomach conditions (1H). **Leaves**, eaten in salad, are slightly laxative (2H). **Flower and leaf infusion** is a refreshing (7H) and emollient (3H) beverage *MED:* **Mauve infusion** together with mint and chamomilla is a remedy for gastritis (1H). Mauve **baths** deflate the feet (1P). Mauve **compress** gives relief in case of injuries (4P). **Leaves** are chewed to calm toothache (1P), or used in **wraps** on inflamed gums (1H). Grounded leaves mixed with milk in **cataplasm** cure pimples and promote expulsion of foreign matter (1 M). **Leaves** are used to relieve burnings of the intimate areas (9H) and the **decoction** is used for intimate and refreshing lavenders (1P, 3 M), for gastrointestinal affections (2P), to promote digestion (1H), for inflammation of oral cavity (3P, 3H, 5 M) and throat (1H). **Leaf infusion** is refreshing (1P, 3 M), soothes intestine (1H,2 M), it cures intestinal disorders (1H) and intestinal (2H) and mouth (2H) inflammation; it purifies urine (1H), relieves belly pain (2H), and detoxifies in case of various infections (1 M), it is diuretic (1H), digestive (1H), emollient in case of heartburns and stomachache (6H), it is also used to reduce anxiety and to induce sleep (2H). Leaf infusion together with blueberry and oak bark is in **wrap** against hemorrhoids (2H). **Leaf boiling water** (after filtration) is used against toothache (1 M), inflamed gums (1 M), drunk as digestive (1 M) or in case of cough (1 M). The leaves after decoction are placed between **gauze** pads and laid on painful body parts (1 M). **Leaf juice** has healing activity (2H). **Flower and leaf juice** relieves painful stings (1H), mixed with olive oil is used to treat burns and shingles (1H), while warmed in olive oil is useful in case of earache (1H). **Flower infusion** is drunk in case of cough (2 M), and catarrh (2 M), sore throat (3 M) and gingivitis rinses (2 M); it is used for external rinses in cases of vaginitis (1 M), and in **compress** it treats skin diseases (2 M). **Leaf and flower decoction** regulates intestine functions (5H), is digestive (1H) and anti-inflammatory for the intestine (1H). It is used for rinsing the mouth in case of irritation (2 M, 5H), for toothache (2P), gums ache (1P), stomachache (13P, 5H), abdominal spasm (2P, 4H, 1 M), cough and sore throat (12P, 7H 1H, 2 M), since it is expectorant is used in case of bronchitis (1 M, 2H). In **compress** it treats skin diseases (4H, 6 M), itching and skin redness, and refreshes the skin of newborns after inflammation due to diaper (1H). It is used against hemorrhoids both **in cataplasm** and ***per os*** (1 M) with honey (1P). The same decoction, together with apple, lemon, chestnut and honey is cough sedative (3P). **Leaf and flower infusion** is digestive (1H), it cures kidney issues (1H), colitis (7H), constipation (2 M), and cough, by cleaning the respiratory tract and removing catarrh (6H). It is used for rinsing in case of mouth irritation (1 M) and inflamed gums (2H). **Aerial part decoction** protects genitals (1H), relieves hemorrhoidal pains (3H 1H), and it is used in lenitive intimate lavenders (4H), **Aerial part infusion** relieves cough (6H), reduces gastritis pain (2H), and it is used in disinfectant mouthwashes (1H), and for mouth affections (6H); in **compress** it is used against toothache and abscesses (5H), as anti-inflammatory, analgesic and as a remedy for rheumatic pain (4H), applied on chest is as anti-tussive (1H). **Whole plant decoction** is diuretic (2H), enhances digestion (1H), is used for constipation (1H), toothache, oral cavity inflammation (22H), irritated skin (7H), to disinfect the throat (2H) and to clean kidneys (1H, 1 M). **Decoction of leaves, decorticated roots and flowers** yields an oily liquid which is laxative (4 M), promotes digestion (1 M), cures stomach and intestinal conditions (1 M), and gingivitis (4 M). The **decoction of roots** (boiled for 3 h) is used for gastrointestinal affections (2P), is beneficial for liver and kidney (1 M), it is applied topically as plagues healer (1H). **Leaf and root syrup** together with laurel leaves is a remedy for hemorrhoids (3H) and cough (2H) *COSM:* **Flower infusion in compress** softens the skin (2 M). **Leaf infusion** is applied on the face to promote suntan (1H) *VET:* **Leaf infusion** promotes digestion and rumination in grazing livestock (2H)
*Marrubium vulgare* L. BOLO0029938	Marrubio comune Marubbio Marrobbi	Wild-native	*MED:* **Whole plant macerate** is cough sedative (2H). **Leaf infusion** is laxative (1 M)
*Matricaria chamomilla* L. BOLO0049060	Camomilla Camamella Camumèla Camumélla	Wild-native	*FOOD:* The **flowers** are macerated for 20 days in alcohol, then filtered and added to a solution of water and sugar to obtain a liquor (3P). **Flower infusion** is refreshing (6H) *MED:* **Flower infusion** has relaxing proprieties (4P, 49H, 10 M), it calms the nervous system (1H), promotes sleep (11P, 24H, 10 M), is calming for kids (1H). It relieves abdominal (3P, 9H, 2 M), and menstrual pain (5H), and colic in infants (1H), and has beneficial properties for gastrointestinal system (2 M). It is diuretic (1P, 3H), digestive (1P, 1 M, 2H), purifier (1P), calms cough (1P), phlegm, cold and sore throat (3H), and it disinfects the first respiratory tract (12H), it is used for sore gum (1P), to wash oral cavity (2H). With the addition of salt it cures “giradito” (whitlow) (2H), and, assumed with a drop of olive oil, it has a laxative effect (7P, 4H). It is used to wash reddened eyelids (1P), and eyes (7H, 2 M), and in case of conjunctivitis (7P, 11H), and to wash genitals (2H), and as derivative enema (1H). Cooled flower infusion is applied in the ears to treat otitis (1H). **In compress** it is used on inflamed mucous and skin (1H), as an anti-inflammatory (4P), against candidiasis (1P), abscesses and toothache (3H). In case of injuries the **wrap** of the infusion calms pain (1P). Flower infusion together with lavender, violet and sage is anti-arthritis (1H). Chamomilla infusion together with mint and mauve is useful in case of gastritis (1H). The **decoction** of mauve together with mint is useful against cold and catarrhal (2H). **Flowers** are used for fumigation (added to boiling water) for stuffy nose (1H) *COSM:* **Flower decoction** (1H) and **flower infusion** (5P, 16H, 1 M) are used to lighten the hair. **Flower infusion** is used as body and face cleaner (1H). Flower infusion is used to do relaxing baths (1 M) and footbaths (1H)
*Medicago sativa* L. BOLO0049492	Erba medica Erba medga Spagnèra Spagna Spâgna	Wild-alien	*FOOD:* **Fresh flowering tops** are eaten in salad (1H) *MED:* **Leaves** in **cataplasm** have anti-hemorrhagic action (1P). **Flower infusion** is useful for people suffering of rickets (1P), it improves blood circulation (1H) and digestion (1H). **Whole plant infusion** has restorative properties (5H), purifies the body (3H), and is galactagogue (2H) *AGROPA:* The **whole plant** is eaten by cattle (2P) and used as fodder (8H). The plant is cultivated to enrich the soil (1H). The **flowers** attract bees which make honey (1H) *DOM:* **The root** is used as stove fuel (1P)
*Melilotus officinalis* Pall.BOLO0048756	Erba sulfanenna	Wild-native	*MED*: **Fresh flower juice** is rubbed on eyelids to relieve inflammations (1 M), **flower infusion** is used **in compress** for eyes and eyelids inflammations (1 M)
*Melissa officinalis* L. BOLO0052727	Melissa Êrba limauna Erba limona Erba cedrina Êrba Limåuna Êrba Limåuna	Wild-native	*FOOD:* A digestive (6H) liquor is prepared with **leaves** (1P). **Leaves** are used in cookery to flavor several dishes (2H), and drinks (1 M). **Leaf infusion** is refreshing (2H), and bracing (2H) *MED: ***Young leaves** are eaten to fight halitosis (3H). **Leaves** are rubbed on mosquito bite (7P). **Leaves** and lemon **macerate in alcohol** is digestive (1P) and calms headache (1P). **Leaf infusion** has choleretics (1 M), digestive (10H, 4 M) and depurative (3 M) properties, it is relaxing (9H) for the gastrointestinal system (3 M), sedative (1H, 3 M) and useful against abdominal cramps (2 M), swelling (1 M) and migraines (2H, 1 M). It was believed that cloistered nuns used to drink leaf infusion to decrease sexual energy (1 M). **Aerial part infusion** is calming (1H) and digestive (7H), it reduces insomnia (6H), headache (3H), gastrointestinal pain (1H), stomachache (2H), and sore throat (1H). **Dried aerial part decoction** improves moodiness and memory (1 M), induces sleep (4 M) and relieves stomachache (1 M) *REP:* **Bunches** of lemon balm are hung to keep insects away from clothes (1P). It is planted since it repels mosquitoes (2H) *DOM:* **Leaves** are used to perfume the clothes in the wardrobe (5H) *COSM:* **Fresh leaves** are used to polish the tooth (1P)
*Mentha* spp. -	Menta Mæinta Mìntoccia Mintâster Mintastar Mänta Mintâster Maͤinta	Wild-native	*FOOD:* **Leaves** are used in cookery (1H), to flavor tea, infusions (4P), beverages, meat (6 M), and several dishes (5H 2H,2 M). Leaves are used to prepare a liquor (14H), obtained by maceration in alcohol for 20 days, then water and sugar are added; it is filtered, and it is drunk since it is digestive and refreshing (2 M). **Leaf infusion** is refreshing (2P, 7H, 2 M), and has bracing (6H), digestive (1H) and thirst-quenching (2 M, 8H) properties. **Leaves** are chewed to refresh the mouth (7P, 2H). Fresh leaves are used to prevent the formation of rennet in milk and to prevent fruits from rotting (2H) *MED:* **Leaves** are chewed as anti-halitosis (8H). **Dried leaves** are used on skin to relieve itching (1H). **Cataplasm** of mint leaves and butter are used to soothe itchy skin (2H). **Leaf juice** relieves cephalalgia (1H), **fresh leaves** can be rubbed on the forehead and on the temples to relieve headache (1M). **Leaf infusion** purifies the liver (1H), is diuretic (3H), digestive (12H, 5M), useful in case of stomach acidity (2H) and stomach conditions (1H), it calms hiccup and stops vomit (1H), carsickness and seasickness (1M), and intestinal swelling (1M); it removes halitosis and disinfect mouth (2H), relieves persistent cough (2M) and sore throat (3H).It is used to relieve insect bite itching (7H), is an analgesic used in case of neuralgia and migraine (1M), it is used to massage the temples to relieve headache (1P). Once cooled, water of infusion is used for rinses in case of oral cavity infections (2M) and diseases (1H). Leaves infusion together with thyme, linden, yarrow and honey is used as treatment against pimples (7H). Infusion of mint, rosemary and sage leaves is useful in case of arthritis (2H). Mint infusion together with chamomilla is useful in case of gastritis (1H). **Leaf decoction** is used against stomachache (2P) and to promote digestion (1P). **Flower infusion** together with chamomile is used in fumes against cold (3H). Flower infusion together with violet, elder and linden is used in case of cold (1H). **Aerial part infusion** is digestive, cures stomachache, and abdominal pain (3H), and in mouthwashes treats toothache (1H) *COSM:* **Whole plant** is a refreshing ingredient for warm baths (1M). **Fresh leaves** together with bicarbonate is used as toothpaste (1H). **Leaf infusion** is used for breath scenting (1H) *REP:* **Leaves** are rubbed on skin to keep mosquitoes away (2P). The plant is used to keep mice away (1M) *SMR:* **Infusion of mint leaves** was believed to cause male impotence (1 M) *VET:* Racehorses are fed with mint to purify their blood and make their coat shinier (1H). **Leaf infusion** is used to purify livestock liver (1H)
*Mercurialis annua* L. BOLO0052395	Mercorella comune Marcurèla	Wild-native	*MED:* **Aerial part infusion** is used as laxative (1H), or in case of indigestion (1H)
*Mespilus germanica* L. BOLO0052613	Nespolo Nèspel Næspel	Cultivated	*FOOD:* **Fruits** are eaten (2H) since they are rich in sugars (2 M) and nutrients (4H), they are used to prepare jam and sweets (12H, 1 M). Fruits have anti-inflammatory (1 M), diuretic (1 M) and laxative (2H, 1 M) properties. Eating fruits helps in case of diarrhea (1H) *MED:* **Unripe fruits** are astringent (1H, 2 M). **Raw fruit decoction** is useful in case of diarrhea (1H). **Leaf and fruit decoction** in mouthwash cures oral inflammation (1H). **Dried bark decoction** is used to treat diarrhea (2 M), inflamed throat (1 M) and fever (1 M) *TOXIC:* **Nut** should not be eaten because is toxic (1 M) *OUI:* Eat too many fruits might cause hemorrhoids (2H)
*Meum athamanticum* Jacq. BOLO0602033	Finocchio selvatico	Wild-native	*FOOD:* **Leaves and stems**, harvested in spring, are eaten for their deflating and digestive properties (2 M)
*Morus alba* L. BOLO0055382	Gelso Gels Måur bianc	Wild-alien	*FOOD:* A jam is made with the **fruit** (2P, 2H) *MED:* **Fruit juice** is a remedy for sore throat (1H) *AGROPA:* **Leaves** were used to feed silkworms (2P, 3H)
*Morus nigra* L. BOLO0053168	Gelso Fòia dla mora Måur naigher	Wild-alien	*FOOD:* **Fruits** are edible (2H) *MED:* A **jam** made of **fruits** is a remedy for sore throat (2H)
*Morus* spp. -	Gelso	Wild-alien	*FOOD:* **Fruits** are used to prepare jam and sweets (6H). **Fruits** are sweet, they promote digestion (1 M) and have beneficial properties for the intestine (1 M) *MED:* **Bark decoction** is laxative (2H) *CRAFT:* The **wood** is used to make pipes (1H) *AGROPA:* Leaves were used to feed silkworms (1 M), whose eggs were kept worm, laced in patches near women's breasts
*Musa paradisiaca* L. BOLO0048458	Banano	Cultivated	*MED:* **Fruits** are astringent (1H), they are eaten to prevent muscle cramps (1H), since they are rich in magnesium (1H)
*Myosotis arvensis* Hill BOLO0003328	Non ti scordar di mé	Wild-native	*MED:* **Aerial part decoction** in **compress** is used for eye inflammations (1 M). **Flower decoction** has a relaxing effect (1 M)
*Narcissus jonquilla* L. BOLO0048662	Giunchiglia Zunchellja Carsan	Wild-alien	*MED:* **Flower infusion** is anti-diarrhea (1 M), antispasmodic (1 M), sedative (2 M) and sleep-promoting (1 M), and it is a remedy for cough (2 M) and asthma (1 M). In **compress** it is applied on burns (1 M)
*Nasturtium officinale* R.Br. BOLO0052373	Crescione Cærson Carsån Carsån	Wild-native	*FOOD:* **Leaves** are eaten in salad or soup (2H, 1 M), they stimulate the appetite (1 M), and are tonic due to the content of vitamins and minerals (2 M) *MED:* The **cooking water** is anti-inflammatory for the intestine (2H), the **decoction** is refreshing and used to treat urinary tract inflammation (1 M). **Leaves** are used in **cataplasm** to treat pimples (1 M)
*Nerium oleander* L. BOLO0602034	Oleandro	Cultivated	*MED:* **Leaf decoction in lavender** is lenitive for hemorrhoids (1H) *REP:* **Flower infusion** has insecticidal properties (1P)
*Nicotiana tabacum* L. BOLO0013908	Tabacco Tabac Tabâc	Cultivated	*REP:* **Leaf macerate** is used as insecticide (2H)
*Ocimum basilicum* L. BOLO0014555	Basilico Basalecc Basaléc Ba§alécc	Cultivated	*FOOD:* **Leaves** are used in cookery to flavor several dishes (16H, 1 M), and to prepare a digestive **liquor** (1 M) *MED:* **Leaf infusion** is digestive (8H), diuretic (2H) and refreshing (2H), and it is useful against vomit (1H). **Leaf decoction** calms anxiety (1 M), and it is applied to the inflamed part of the oral cavity (1H). **Leaf macerate** cures sore throat (1H) and cold (1H) *REP:* **Leaves** rubbed on skin keep insects away (3H)
*Oenothera biennis* L. BOLO0049289	Enagra comune Pedga ‘d esan	Wild-alien	*MED:* **Root decoction** relieves stomachache (1H)
*Olea europaea* L. -	Olivo Uliv Ulîv	Cultivated	*FOOD:* **Olives** are purifying, so they are eaten before drinking alcohol. (1H) *MED:* **Olive oil** is applied on burns to prevent blister formation (2H), and it cures ear infections (2P), and soothes earache caused by otitis (16H). Two spoons of olive oil are helpful against gallstones (1H), and stomach swelling (1H). Olive oil is put on stye to heal it quickly (3H). One teaspoon of olive oil a day on an empty stomach promotes intestinal transit (9H). Elder bark is boiled in olive oil to obtain an ointment useful against burns (2H). **Leaves** together with elder are used to make an **oleolite** useful against burns and dry skin (9H). Olive oil together with a garlic clove is used to make an ointment useful against intestinal worms (2H) *COSM:* **Olive oil** makes hair shinier (2P) *SMR:* The farmers plant an **olive branch** at the beginning of orchard rows in sign of good omen (1P). An olive tree branch represents peace (6H), it is used to bless the wheat field (1H), it was burnt to ward off the hail, which ruins crops (3H),
*Onobrychis viciifolia* Scop. BOLO0003054	Lupinella Erba crocetta Lupinæla	Wild-native	*MED:* **Aerial parts** are used to disinfect small wounds (1H). **Flowers** attract bees which make honey, useful for sore throat (1H) and tiredness (1H), and as liver purifier (1H) *AGROPA:* **The plant** is used to feed livestock (3H), sheep have a higher quality wool if they are fed with this plant (1H)
*Ononis spinosa* L. BOLO0053004	Ononide Ligabó Êrba spinósa Bunæga	Wild-native	*MED:* **Root extract**, prepared in vinegar and cold water for 10 min, is used for gargling to treat sore throat (2 M), as a mouthwash to protect inflamed larynx and bleeding gums, and to calm pain associated with caries (1 M)
*Origanum majorana* L. BOLO0014611	Maggiorana Mazurèna Mażurèna Mazurèna salvâdga Mażurèna salvâdga	Wild-alien	*FOOD:* **Leaves** are used to flavor foods (3 M) and sauces (1 M). **Aerial parts** are used to prepare several dishes (3H), they stimulate the appetite and are digestive (6H) *MED:* Fresh or dried **leaves infusion** cures cold (2 M), cough (2 M), it is digestive (1 M) and useful in case of migraine (1 M)
*Origanum vulgare* L. BOLO0053946	Origano Urêghen	Wild-native	*FOOD:* **Aerial parts** are used to prepare several dishes (3H), they stimulate the appetite and are digestive (6H,1 M) *MED:* **Flower infusion** is digestive (1H) and useful against intestinal conditions (1H). The **essential oil** is useful as an antibiotic (2H) against candida (1H) and as a remedy for burns and wounds (2H). Fresh or dried **leaves infusion** is digestive and is a remedy for heartburns (1 M)
*Oryza sativa* L. BOLO0020594	Riso Ris Rîs	Cultivated	*MED:* **Fruits** are boiled and eaten in case of diarrhea (9H) and intestinal conditions (1H)
*Osmunda regalis* L. BOLO0049945	Felce florida	Wild-native	*MED:* **Aerial part infusion** is abortive if it is drunk every day (1H). **Whole plant** (including root) **infusion** is abortive (1H). **Leaves** are used to wrap and heal wounds (3H)
*Ostrya carpinifolia* Scop. BOLO0003030	Carpino nero Carpinæla	Wild-native	*FOOD:* **Bud infusion** is regenerating (1 M) *MED:* **Bud infusion** is draining (1 M), and useful for sinusitis (1 M) and cold (2 M) *DOM:* The **wood** is used as fuel for the fireplace (1 M)
*Oxalis acetosella* L. BOLO0047854	Acetosella Êrba bròssca	Wild-native	*FOOD:* **Leaves** are eaten in salad and are purifying (4H) *MED:* **Leaf decoction** is diuretic (1H) *DOM:* **Leaf juice** is used as a stain remover, especially to remove ink or rust from clothes and for this property it was used also to polish copper pots (1 M) *SMR:* **Flower and leaf** are used to cast spells, and burnt to bring good luck. The **plant** growing in the garden brings good luck (1H)
*Paliurus spina-christi* Mill. BOLO0052515	Spèn marug	Wild-native	*FOOD:* **Fruits** are eaten and taste like apples. (1H) *MED:* **Fruit decoction** has diuretic properties (1H)
*Papaver rhoeas* L. BOLO0055375	Papavero comune Papâver Rusætt Ruṡatt Ruʃaͤtt	Wild-native	*FOOD:* **Leaves** are used to prepare several dishes (2P, 1H, 1 M), **leaves and flowers** are eaten in salad (1H). **Flowers** are a spice used in cookery (1 M) *MED:* **Flower infusion** is sedative (12H), it promotes sleep also in kids (1H), it calms cough (1H), removes catarrh (1H) and purifies liver (3H). **Seed infusion** is drunk to induce and promote sleep (2 M), especially used to asleep children (3H). **Latex** is a cough sedative (3H) and induces sleep in kids (3H) *COSM:* **Whole plant decoction** has anti-wrinkles properties (1 M) *AGROPA:* It was used as rabbit feed (1 M). **Whole plant** is considered a weed species for the grain (1 M) *CRAFT:* Once the girls used the **buds** to make dolls (1 M)
*Parietaria officinalis* L. BOLO0052779	Erba vetriola Parietaria Erba venta Vidariôl	Wild-native	*MED:* **Fresh leaves** are chewed to calm toothache (1H), and rubbed on the skin to soothe the itching caused by nettle leaves (1H). **Leaf decoction** is used in case of edema (1H), it is diuretic (1H) and it is used to make **wrap** on burns and skin irritations (1H). **Dried leaf tea** has diuretic properties, and it is beneficial for the urinary tract (2 M). **Aerial part decoction** is a remedy for fever (2H), colic, stomachache (2H), and catarrh (2H). **Whole plant decoction** is diuretic and it is used against cystitis (2 M), bladder pain (1P), and pains due to the menstrual cycle (1P), it was also used to treat scarlet fever (1 M) *DOM:* **Leaves are** used to clean the glass (2 M), also **aerial parts** are used to clean bottles and flasks due to their abrasive action (2H), for this reason this plant is called “vetriola” which means glassy (1 M)
*Passiflora caerulea* L. BOLO0011097	Passiflora Fiåur dla Pasiån	Cultivated	*MED:* **Whole plant infusion** is relaxing (6H), and used to soothe burns and skin inflammation (7H) *SMR*: The opening of this plant's flower at dawn symbolizes the Madonna giving us the good morning (1H)
*Pelargonium* spp. -	Geranio Gerâni Girani Gîrâni	Cultivated	*MED:* **Fresh leaves** are applied on burns (1H). The outer face of the leaf, harvested in spring–summer, is placed on wounds to "get the infection out" (1 M). The **leaves** are used to make a relaxing **decoction** (2 M). The water of this decoction, once cooled, is used for rinses against gingivitis (1 M), gargles in case of sore throats (1 M), to wash acne (1 M), to massage temples in case of migraine (1 M), it is applied on the body to reactivate blood circulation (2 M), and in **compress** on burns (1 M) and erythema (1 M) *COSM:* Cooled water of **leaf decoction** in **bandages** is anti-cellulitis (1 M) *REP:* It is an ornamental plant used to keep away insects (9H). Water of **leaf decoction**, placed in vases on the balconies of the windows, keeps mosquitos away (1 M)
*Persicaria hydropiper* (L.) Delarbre BOLO0037076	Pepe d’acqua Erba de pever	Wild-native	*FOOD:* **Flowers** are used to flavor dishes and hunter (2 M)
*Petasites hybridus* (L.) G.Gaertn., B.Mey. & Scherb.BOLO0602031	Farfaraccio Farfalloni	Wild-native	*MED:* **Leaves**, collected in shady places, are a healing agent (1 M), and are used in poultices on rheumatism of the knees (1 M), their **boiling water** is drunk as antispasmodic for the intestine (1 M) *AGROPA:* **Leaves** are used to shelter the seedlings of tomato plants of the garden from wind and cold (1 M)
*Petrosedum rupestre* (L.) P.V.Heath BOLO0007413	Sedum	Wild-native	*MED*: Flowering plant is harvested in June, **leaves** are crushed and rubbed on leeks and warts to heal (3 M)
*Petroselinum crispum* (Mill.) Fuss -	Prezzemolo Pidarsùl Prasôl Parsemul	Cultivated	*FOOD:* **Leaves** are used in cookery to flavor several dishes (18H, 1 M) *MED:* **Leaves** were eaten to abort (3P, 10H), and to reduce hernia swelling (1H). Fresh leaves are applied on insect bites (3H), and on sore udders after breastfeeding (3H). **Fresh leaf juice** is used in compress on tired eyes (1 M), and **in wraps** for conjunctivitis (1H). **Leaf pulp** together to vinegar is used to blindfold contused parts (1H). A ball is made with leaf pulp together with salt and olive oil and used against toothache (1H). **Leaf decoction** has an expectorant action useful against cough (1P), it is digestive (1 M), diuretic (1 M), and anti-lice (1 M). **Decoction** of parsley and sage leaves is useful in case of lack or delay of menstrual cycle (3H), the same effect is obtained by eating fresh leaves (1 M). **Leaf infusion** is purifier (5H). **Syrup** made with fennel roots, parsley, celery and butcher’s broom reduces intestinal gases (6H). **Infusion** of parsley, fennel and aniseed is a remedy for stomach acidity (3H). A **beverage** obtained by mixing ten **stems** of parsley in one liter of wine is drunk once a day to reduce heart pain (1H) *VET:* **Leaves** are used to get cats to abort (2P, 2H)
*Phaseolus vulgaris* L. BOLO0040218	Fagiolo Fasôl	Cultivated	*FOOD:* **Seeds** are consumed in salads for their protein content (3 M). To eat high quantity of beans prevents intestinal conditions (1 M), constipation (2 M) and hemorrhoids (1 M) *MED:* **Infusion of bean peel** is useful against gout if it is taken daily for a week (2H). **Seeds** are used as slimming ingredients in high fat meals because they reduce the fat absorption (1H) *GAME:* **Seeds** dried in the sun are used as number markers in the game of bingo (1 M)
*Phedimus spurius* (M.Bieb.) 't Hart BOLO0014087	Sedum	Wild-alien	*MED:* Peeled **leaves** applied on pimples and skin inflammation are healer (2 M)
*Phytolacca americana* L. BOLO0046782	Fitolacca	Wild-alien	*OUI:* The red **berries** are used to color white wine, making it red and thus more valuable to sell (2P)
*Picea abies* (L.) H.Karst. BOLO0015195	Abete rosso Abæid Abaͤid	Wild-native	*MED:*** A drop of resin** a day cures respiratory conditions (3H), and heals wounds (1 M). **Branches** are placed in the warm **bath** to help in case of rheumatisms, and for the same issue it is used the branch **decoction** in **compress** (1 M)
*Pilosella officinarum* Vaill. BOLO0023244	Pilosella	Wild-native	*MED:* **Whole plant infusion** promotes edema resorption and reduces leg swelling (1H) *COSM:* **Aerial part** is used to prepare an ointment that reduces cellulitis (1H)
*Pimpinella anisum* L. BOLO0031209	Pimpinella Anice verde	Cultivated	*FOOD:* **Fresh leaves** are eaten as liver purifier (1H) *MED:* **Infusion** of parsley, fennel and aniseed reduces stomach acidity (3H). **Leaf infusion** is useful as intestinal anti-inflammatory and purifying agent (1H). **Seed lotion** is applied on hair as anti-lice (1H). **Seed infusion** stops hiccup (1H) *REP:* The **whole plant** keeps mosquitos away (1H) *AGROPA:* **The plant** is used to feed farm animals (2H)
*Pinus cembra* L. BOLO0054088	Pino Pén	Cultivated	*MED:* **Cone macerate** is useful against cough (5H)
*Pinus mugo* Turra BOLO0021781	Pino mugo Pino speciale	Wild-native	FOOD: The **cones** are used to make a liquor (1 M) *MED:* The **cones** are used to make a balsamic (1H) and cough sedative (2H, 6 M) **syrup**
*Pinus pinea* L. BOLO0052235	Pino Pén	Cultivated	*FOOD:* Pine **nuts** are largely used in cookery (13H)
*Pinus* spp. -	Pino Pén		*MED:* **Resin** is used in fumigation for respiratory tract disorders (7H), such as cough (1H) and colds (1H). Resin is spread on a toast, and it is eaten to reduce pregnancy associated nausea (1H). **Leaf infusion** is expectorant (1H)
*Pinus sylvestris* L. BOLO0002747	Pèn Pén Pen salvâdg Peṅ salvâdg	Wild-native	*FOOD:* **Leaves** are rich in vitamin C (1H) *MED:* **Bud decoction** is expectorant, cough sedative (1P), useful in case of flu, catarrh and bronchitis. **Bud infusion or fumigation** have expectorant and antiseptic activity (1H, 3 M). **Resin** is applied on broken arm to reduce pain and to promote healing (2H)
*Plantago afra* L.BOLO0602035	Psillio Psél Piantâzen Laͤingua d’ôca	Cultivated	*MED:* **Seeds** are put in water to make a laxative jelly (1H)
*Plantago lanceolata* L. BOLO0053695	Piantaggine Piantagine Længua d’ôca Lingua d'oca Urec d’esen Êrba di zinq neruv Lengua d'òca Lengva ‘d can Lèngua d’oca Piantâʒen Piantâzen Laͤingua d’ôca	Wild-native	*FOOD:* **Leaves** are used in cookery to prepare salads and soups (1 M, 3H) *MED:* **Leaves** are astringent (1 M) and anti-inflammatory (1 M), used for gargling in case of inflamed throats (1 M) and gingivitis (1 M). The crushed leaves are used to heal wounds (1P, 2H) and in **cataplasm** to relieve itch and inflammation due to insect bites (1 M). **Leaf infusion** is refreshing (1 M), purifying (1 M) diuretic (1 M), and used as a cough remedy (1 M), **in compress** it is placed on burnt areas of the body (1 M), on insect bites to relieve itching (1 M), and to treat conjunctivitis (1 M). **Leaf decoction** is laxative (1 M). The **infusion of seeds** is used for gastrointestinal issues (1 M) and as nasal decongestant (1 M) *VET:* Rabbits are fed with the **whole plant** to prevent dysentery (1P)
*Plantago major* L.BOLO52180	Plantago Urach d'esen Zentnòd	Wild-native	*MED:* **Fresh leaves** are applied on sores, wounds or skin rashes to promote healing (1H). **Leaf infusion** is used against cough (1H) and sore throat (1H), it is astringent (1H), reduces hemorrhoids (1H), and purifies intestine (1H). **Seed decoction** regulates intestinal functions (1H)
*Plantago* spp. -	Piantaggine	Wild-native	*MED:* **Leaves** are placed on pimples, abscesses, redness and wounds to facilitate healing and as anti-inflammatory (10 M). Fresh leaves are placed on insect bites or skin irritation to calm pain and reduce the irritation (1H, 5 M). Fresh leaves are pestled and used as **poultice** to heal perioral dermatitis (1 M). **Leaf in cataplasm** are used to heal wounds (3H) and furuncles (3H). **Leaf infusion** calms cough (1H) and sore throat (1H), itis intestinal astringent (1 M) and purifying (1 M). **Leaf decoction** is used against skin reddening, pimples and insect bites (2 M) *COSM:* **Leaves and roots** are chopped, boiled and added with vaseline and essential oils to make an emollient **ointment** (1 M). **Leaves** are **boiled** in milk and the resulting extract is used on dry skin (1H) *AGROPA:* **Leaves** are used to feed livestock since they are energizing (2H)
*Polygala vulgaris* L.BOLO0052892	Poligala Amarella Erba da la tass	Wild-native	*MED:* **Whole plant decoction**, together with *Hypericum perforatum* and *Tussilago farfara* is used against bronchitis as expectorant (2 M). **Root decoction** is used to calm cough, including persistent cough (1 M)
*Polygonum aviculare* L. BOLO00522220	Centonodi Cruzola Coreggiola	Wild-native	*MED:* **Aerial part decoction** of is used to stop bleeding and hemoptysis (1 M), and the **juice** of this plant is used to heal wounds (1 M). **Aerial part infusion** is expectorant, and it is used to relief upper respiratory ways (1H) *OUI:* **Aerial parts infusion** was given to kids to slow down their growth and delay the recruitment to the front (1H)
*Polypodium vulgare* L. BOLO0052410	Falsa liquerizia Felce dolce Finta liquerizia Faelza dulza Faͤllza dåulza	Wild-native	*FOOD:* **Roots** are chewed to quench hunger and thirst and for its licorice like taste (2H), which makes it appealing for kids (3 M). It is eaten because it reduces thirst, since it is plenty of water (1H). Roots are also eaten for their digestive and beneficial properties on the intestines (1 M) *MED:* It is chewed in case of sore throat (1H). **Root decoction** is laxative (1 M). **Leaf and root decoction** is vermifuge (1 M)
*Populus alba* L. BOLO0052832	Pioppo bianco	Wild-native	*MED:* **Bark decoction** is a remedy for stomachache (1H) and diarrhea (1H) *SMR:* To sleep under a poplar during a rainy night makes a desire come true (1H)
*Populus nigra* L. BOLO0017028	Pioppo Fiòp Piòp Fiòpa	Wild-native	*MED:* **Buds decoction** fluidifies bronchial secretions (2H) and promotes sweating (2H) *COSM:* **Buds** together with poppy and lettuce are mixed with pork fat to obtain an ointment useful for dry hands (1P) *VET:* **Branches** are used to feed rabbits to control their growth and strengthen the teeth (1H)
*Portulaca oleracea* L. BOLO0053723	Porcellana Purʒlœna Êrba grâsa	Wild-native	*FOOD:* It is eaten in salad (3P) since it is rich in minerals and iron (2P). **Leaves** are eaten because they are purifying (2H) *MED:* **Fresh leaves** are **wrapped** on wounds, furuncles, and bee stings (9H). It is used to prepare a laxative **infusion** since it is rich in mannitol (1P). **Whole plant infusion** is useful to stop diarrhea, vomiting (3H), and *post-partum* hemorrhages (3H), it improves sexual performance (2H)
*Primula ciliata* Moretti BOLO0047214	Primula orecchia d’orso	Wild-native	*FOOD:* **Leaves** are cooked or eaten raw (1 M) *MED:* **Leaves** are cleaned and used to make a diuretic (1 M), sedative (1 M) for cough (1 M), and anti-inflammatory (1 M) **infusion**. **Leaf decoction** is applied in **bandages** on the areas affected by rheumatism (1 M). **Root decoction** is an excellent diuretic (2 M) and anti-diarrheal (1 M); it is effective in case of cough (2 M) and as an anti-nausea (1 M). Water is added to chopped roots and used in bandages to heal muscular pain (1 M)
*Primula* spp. -	Primula Prèmla Premmivair	Wild-native	*FOOD:* **Leaves** are used to prepare “green lasagna” (2 M). Children suck the **flower** for its sweet nectar (1 M) *GAME:* Children enjoy blowing the yellow primrose flowers like whistles (1 M)
*Primula veris* L. BOLO0052883	Primula Premmiveir Premmavaira	Wild-native	*FOOD:* **Flowers** are eaten in salad (6H) *MED:* **Root and flower infusion** calms cough, promotes catarrh expulsion (6H), and activates blood circulation (1H). **Flower infusion** is used against gout (1H). **Leaf infusion** calms muscular pain (1H) *COSM:* **Pounded flowers** are applied on the skin to make it stronger and younger (1H) *GAME:* Children enjoyed blowing the yellow primrose flowers like whistles (5H)
*Primula vulgaris* Huds.BOLO0003314	Primula	Wild-native	*FOOD:* It is used to prepare several dishes (8P, 1 M). **Flowers** were sucked for their sweet taste (3 M)
*Prunus armeniaca* L. BOLO0040379	Albicocco Biricóquel Mugnèg	Cultivated	*FOOD:* Dried fruits are eaten since they are remineralizing (8H) *MED:* **Fruits** are eaten against intestinal parasites (5H). **Fruit infusion** is useful in case of high fever (1H). **Pulp decoction** is used to cue ear conditions (1H)
*Prunus avium* L. BOLO0047972	Ciliegio Zrìs Zris Zrî§	Wild-native	*FOOD:* **Cherries** are used to make a refreshing drink (1P), jams, and candies (2P, 7H). Fruits are eaten (3H) and have purifying properties (9H), are rich in vitamins and nutrients (1 M). The darkest cherries are eaten as anti-inflammatory (1 M). Fruits eaten in large quantities have a laxative effect (9H) *MED:* **Fruits** are mild laxative (3 M), depurative (2 M) and heart protective (2 M). **Fruit juice** stops diarrhea (9H). **Fruit infusion or decoction** are used to treat urinary tract conditions (4H). **Petiole decoction** has diuretic proprieties (1P, 6H), it is also useful to lower fever (2H), and to relieve kidney pains (1H). **Petiole infusion** is a diuretic (1H, 1 M), purifying and antibacterial (1 M) and useful to eliminate toxins (1 M). **Seeds** are inserted in a small bag, which is warmed up and applied on the neck to fight neck pain (5H, 1 M), or on body parts affected by rheumatism (2H). **Seed powder** is used to relieve muscular and joint pain (3H)
*Prunus cerasifera* Ehrh. BOLO0052581	Rusticano Rusticàn	Cultivated	*FOOD:* **Fruits** are laxative (1H) and are used to prepare a jam (2H). Fruits are eaten immature for their acid taste (7H)
*Prunus cerasus* L. BOLO0052556	Amarasco Mârasca Zreza marasca	Wild-alien	*FOOD:* The **fresh fruits** are eaten in summer because they are very rich in vitamins and nutrients, they are useful to reduce heat (2 M), and, together with cherry fruits, are used to make jams (1 M). Fresh fruits are laxative (1 M). Fruits are used to prepare a digestive liquor (10H) named “maraschen” (2H, 1 M) *MED:* **Petiole macerate** has diuretic properties and calm intimate burns caused by the cystitis (8H). The **fruits** have diuretic properties (1 M) and help to prevent heart conditions (1 M). **Fruits macerated** for 40 days in sugar yield a diuretic juice (1 M) *DOM:* twigs, stripped of their fruits, are bitter and are used, along with the leaves, to preserve vegetables, which then turn out to be more digestible (1 M)
*Prunus domestica* L. BOLO0052554	Susino Pròggn Prògn Proggn	Wild-alien	*FOOD:* **Fruits** are eaten since they are rich in vitamins (3 M) and minerals (1 M). Fruits are used to prepare jam (3H) rich in minerals and vitamins (4H) *MED:* **Fruit decoction** and **dried fruits** promote intestinal transit (7H), calm cough (2H) and improve liver functions (2H). **Fruit decoction** is a tonic against all diseases (2H) and it is laxative (6H). **Leaf decoction** is useful against intestinal worms (1H). **Leaf infusion** of plum tree, rose hip and alder buckthorn is useful against constipation (3H)
*Prunus dulcis* D.A.Webb BOLO0047959	Mandorlo Madṅel	Cultivated	*FOOD:* **Almonds** are used in cookery (10H) *MED:* **Almond oil** is used against skin diseases (10H), intestinal parasites (2H) and to relax muscles (4H). Almonds are eaten on an empty stomach to lower the fever (2H) and to relieve nausea and vomiting in pregnant women (2H). **Almond nutshell decoction** (to take daily for ten days) is useful to cure whooping cough (2H) *COSM:* **Almond oil** is used to soft skin (1H)
*Prunus laurocerasus* L. BOLO0053691	Lauro Lauræl	Wild-alien	*FOOD:* **Ripe fruits** without seeds are macerated in alcohol for half month to prepare a digestive liquor called “laurino” (2H, 2 M)
*Prunus persica* (L.) Batsch BOLO0052582	Pesco Pésg Pèsc Pêṡg	Cultivated	*FOOD:* **Fruits** have restorative properties (5H). The fruits are preserved under syrup (2P, 5H). A wine is obtained with the **leaves** (1P) *MED:*** Fruit infusion** is used as intestinal calming and laxative (2H). **Leaves** are used in **wraps** on the belly to fight intestinal worms (1H). **Leaf infusion** is sedative (1H)
*Prunus spinosa* L. BOLO0053715	Pruno selvatico Sprugnazzi Brugnòl Prugnôl	Wild-native	*FOOD:* **Fruits** are used to prepare a digestive liquor (12H, 3 M), are eaten or used to prepare a jam (3H). **Fruits** are rich in vitamin C and they are useful for seasonal ills (8H). **Fruits** are eaten (7H), help digestion (6H), and purify the gastrointestinal tract (4H). Eaten in large quantities are laxative (4H). Fruits are astringent (1 M) *MED:* **Leaf infusion** is used in case of constipation (2H). **Flower infusion** is digestive (1H) and laxative (1H). **Flowers** are laxative (1 M). **Bark decoction** lowers fever (1H) *DOM:* **Bark** was used to dye the wool of red (1H) *COSM:* **Bark** is cut and used as toothpaste (2H)
*Pteridium aquilinum* (L.) Kuhn BOLO0052409	Felce Felce aquiline Feìlz Fållʒa	Wild-native	*FOOD:* During famine time, a flower was made of the dry **rhizome** (1 M) *MED:* **Leaves** chopped and soaked in water and alcohol are sprinkled on the area affected by rheumatic pain (3 M). **Frond infusion** is a remedy for rheumatism (2 M) *SMR:* **Roots** are used to prepare an **infusion** to drink as a love potion (1 M). This plant is harvested and kept at home as a talisman against the difficulties of life (1 M). A legend says that if this **plant** is harvested at sunrise on June 24th and put in a vase with some coins, it will bring money (1 M). A **seed** collected on the same morning and carried always with you, brings good luck in gambling (1 M). **Root decoction** is used to prepare footbaths or handbaths to soften calluses (2H) *OUI:* **Aerial parts** were used as envelopes to protect fruits during transport (2H). **Root decoction** is used to prepare baths with a relaxing effect (2H) *REP:* It keeps away bugs and parasites (2 M)
*Pulmonaria officinalis* L. BOLO0046792	Polmonaria Erba dla Madòna Pulmonæria	Wild-native	*FOOD:* **Leaves** are fried and eaten (1P) *MED:* **Leaf infusion** is useful against respiratory diseases (1P, 1 M), catarrh (2H), and fever since it improves sweating (5P)
*Punica granatum* L. BOLO0006621	Melograno Maeilgranae Mail ingranè Mailgranè Mæilgranæ	Cultivated	*FOOD:* **Fruit juice** is refreshing and rich in vitamins (7H). **Fruit peel** is used to flavor several liquors (3H). **Fruits** are used to prepare sweets and syrup (4H). **Fruits** are used to prepare syrups and sweets (2H). Eating **fruits** improves blood circulation (1H) *MED:* **Leaf decoction** together with althea leaves stops diarrhea (4H). **Fruit** is mild laxative (1H). **Fresh flowers** are refreshing and disinfect gums (1H). **Flower infusion** is used to wash inflamed gums (2H). **Fruit juice** together with blackberry juice is used to wash the oral cavity in case of pharyngitis (1H). **Fruit peel decoction** is used to lower fever (2H), stop diarrhea (1H), and regularize the intestine (1H). **Decoction of root bark and fruit peel** is used against intestinal parasites (10H). **Bark decoction** (1H) or **root decoction** (2H) stops diarrhea. **Seeds** are diuretic (2H) *SMR:* The plant spreads serenity (1H) and positivity (1H) *DOM:* It is used as an ornamental plant (1H)
*Pyrus communis* L. BOLO0014531	Pero Pere volpine Pere selvatiche Pere rossoline Pæir Per	Cultivated	*FOOD:* **Fruits** are used to make a liquor (1 M). Fruits are macerated to make “grappa” (1P). Fruits are eaten (8H), and in high amounts, they are laxative (3H). The fruits were stored in the cellar amidst straw throughout the summer and eaten in the winter, they are rich in minerals and vitamins (5 M). **Fruits** were usually dried and eaten since they are cheaper than dried fruits (1 M) *MED:* **Infusion of leaves, bark, buds, and flowers** is a diuretic, and it is drunk three times per day to cure urinary tract affections (2H) *CRAFT:* **Wood** is used to craft musical instruments (1H)
*Quercus petraea* subsp. *petrea* BOLO0005624	Rovere	Wild-native	*CRAFT:* **Wood** is used to make wooden barrels (1H)
*Quercus pubescens* subsp. *pubescens* BOLO0016721	Roverella	Wild-native	*FOOD:* The **acorns**, roasted and grounded, were used to make a coffee substitute beverage (1 M) *DOM:* This plant commonly has galls that are rich in tannins and were used to dye tissues (1 M)
*Quercus robur* L.BOLO0052400	Quercia	Wild-native	*FOOD:* The **acorns**, roasted and grounded, were used to make a coffee substitute beverage (1 M) *OUI:* Pigs are fed with the **fruits**, giving their meat a better quality (2P)
*Quercus* spp. -	Quercia Quérza Quêrza	Wild-native	*MED:* **Leaf infusion** soothes oral cavity inflammation (1H). **Shredded bark** is used against epistaxis (2H) and diarrhea (with the addition of rue leaves) (3H). **Bark decoction** is useful in the case of hemorrhoids (3H), it is astringent (2H), anti-inflammatory (2H), and analgesic of first respiratory ways (2H). Bark decoction can be added with blueberry and mauve leaves to do local wraps against hemorrhoids (2H). **Gall powder** is astringent (1H) *CRAFT:* **Wood** is used in carpentry (7H) and to craft wine barrels (2 M) *AGROPA:* **Fruits** are used to feed several animals, such as pigs (18H, 1 M), since they are very nutritious and help pigs’ digestion (1H) VET: **Gall powder** is used against dog eczemas (1H) *COSM:* **Leaf decoction** reduces sweating (1H). **Bark infusion** is used to wash feet and armpits to reduce sweating (1H). Bark decoction is used in a bath to reduce sweating (2H)
*Ranunculus arvensis* L. BOLO0003027	Piè gallo Ranuncolo	Wild-native	*MED:* A small ball made of two **leaves** chopped is placed inside a gauze on the body part affected by swelling; after a few hours or half a day, it forms a blister full of liquid, which is punctured to make the swelling disappear (3 M)
*Ranunculus ficaria* L.BOLO0003338	Calta palustre	Wild-native	*MED:* **Leaves** are used as a remedy against calluses (1 M)
*Raphanus raphanistrum* subsp. *sativus* (L.) DominBOLO0002283	Ravanello Ravanell	Cultivated	*FOOD:* **Root** is edible (6H) *MED:* Radish reduces the risk of kidney affections (1H) *SMR*: For kidney disease treatment, an **infusion** of seven “ravanelli” (radishes) is prepared by letting it boil for 7 min. Then it is important to drink it for 7 consecutive days (1H)
*Raphanus raphanistrum* L. BOLO0002280	Rabarbaro palmato	Cultivated	*MED:* **Root decoction** purifies the liver (2H) and body (2H). A **cream made of roots** is used to calm hemorrhoid pain (1H)
*Ribes nigrum* L. -	Ribes nero	Cultivated	*MED:* **Fruit macerated** in **alcohol and water** is useful in case of asthma (2H). **Fruit jam** is used to cure burns (1H). **Seed decoction** cures flu (1H), rheumatism (1H), and relieves gout pain (1H). **Seeds ointment** is useful in case of eczema (1H) *DOM:* **Fruit juice** was used as ink (2H)
*Robinia pseudoacacia* L. BOLO0053679	Acacia Acâg Acàg Acâǵ Acâg'	Wild-alien	*FOOD:* **Flowers** are eaten fried (1P, 17H, 2 M), are used to prepare sweets (3H), and a liquor (8H). The **young branches** are collected, washed, and chewed to quench thirst (1 M) *MED:* **Flower decoction** is useful against stomachache (3H). **Flower infusion** is laxative (1H), cures sore throat (5H) and respiratory affections (3H). **Leaf and flower infusion** is astringent and is a remedy for diarrhea (1 M), colds (3 M), coughs (3 M), throat inflammations (1 M), and gingivitis in oral rinses (1 M). The **honey** from the flowers is a cough sedative (3H) *AGROPA:* **Flowers** attract bees which make honey (10H, 2 M) *SMR:* The **branches**, characterized by numerous thorns, were kept in the house to ward off spirits (1 M) *CRAFT:* **Wood** is used to make scaffolds since it is very resistant (2 M) *DOM:* **Wood** is burned to heat the house (1H, 1 M)
*Rosa canina* L. BOLO0010048	Rosa sambadga Ròsa salvàdga Rôʃa mâta Pizincul	Wild-native	*FOOD:* The **flowers** are used to prepare sweet pancakes (1P), jam (1P, 2 M), and a refreshing drink (2H), prepared by macerating the petals of 10 flowers in water for a day (1P). With the **petals** a liquor is prepared (1P); it is better not to use the inner white part because it is bitter. **Leaves** are used to flavor tea (1H). **Fruits** are used to make “grappa” (1H, 1 M). **Shoots**, called “peloni” (2 M) were peeled and eaten for their sweet taste (1 M), and as a remedy for sore throat (1 M), and earache (3 M). They are beneficial for the liver (1 M), gums (1 M), and digestion (1 M). The **fresh fruits** are eaten because they are rich in vitamin C (5 M, 1P). Fruits, collected in October–November are used to make jam (10 M, 5H). The fruits, also called “pizzincule”, are sweet and their jam has anti-inflammatory (1 M), vitaminizing (1H, 1 M), remineralizing (17H), and astringent (15H) properties. It also helps to prevent flu (2H), and it cures sore throat (3H) *MED:* **Fruit jam** is astringent (1H, 1 M) and helps to heal the wounds on the sides of the mouth (1 M). **Fruits pulp** is a wound healer (1 M). **Fruits** are **macerated** in water for some days and the obtained liquid is drunk in case of cough and cold (2 M). **Fruit decoction** cures flu (3H, 1 M), because it is rich in C vitamin and other vitamins (7H). **Dry fruit infusion** is drunk in case of coughs (1 M) and colds (9 M). **Fruit and leaf infusion** is used for gargling against sore throat (1 M). A **syrup** useful to treat diarrhea in infants is made with **fruits** by pounding and **boiling** them in water and finally adding sugar (3H). **Flower infusion** is used to wash the oral cavity in case of sore throat (1H) and together with honey is used in case of inflamed mouth (2H) and tonsils (2H), flue (1H). Washed **petal infusion** is drunk in case of cold (5 M), flu (2 M), seasonal allergy symptoms (3 M), cough (3 M), asthma (2 M) and throat inflammation (1 M); the infusion once cooled is used to rinse the eyes affected by conjunctivitis (3 M). The dried petals infusion is laxative (1P). **Petals macerated in water** are used, as eyewash, for reddened eyes (1H), and to treat neonatal candidiasis (1H). Petal **juice** is used in the case of rosacea (1H) and as eye drops to wash eyes (1H). **Leaf or flower infusion** is used in case of diarrhea (3H, 1 M), and it is used to wash mouth or skin (1H) to heal burns and wounds (1H). **Leaf infusion** together with honey and agrimony flowers, is used to wash the mouth in case of difficulties in swallowing (2H). **Leaf infusion** together with plums and frangula bark is laxative (3H). **Leaf and seed decoction** are used in the case of intestinal parasites and intestinal disorders (2H) *TOXIC:* The internal hairs of the fruits are toxic; thus, it is important to remove them before the preparation of the jam (13H) *COSM:* **Petal juice** is used as perfume (1H) and to produce “Acqua di rose” (rose’s water) to lighten the skin (1H). **Fruits** are used to prepare **ointment** toning for the skin (1H). **Fruit pulp** is used to shoot hands (1 M) *DOM:* **Petals and fruits** were used to create natural dye (1 M). **Flowers** are used to perfume linden (3H) *SMR:* It is believed that **fruit decoction** is useful in case of a bite by a rabid dog (1H, 1 M), which is why the plant is called “rosa canina” (doggy rose). It is believed that eating the **fruits** once a day at sunset immunizes against all flu and diseases (1H) CRAFT: **Petals** were collected during Pentecost to adorn the churchyard (1 M)
*Rosa gallica* L. BOLO0047261	Rosa Ròsa Rô§a Rô§a di mis	Wild-native	*FOOD:* **Flowers** are used to make a liquor (8H). **Petals** are used to flavor several dishes (3H) *MED:* **Flower infusion** is used as eyewash for reddened eyes (3H), and to wash the oral cavity in case of sore throat (1H) *COSM:* **Flower infusion** is used for beauty care (12H). A lipstick was made of the petals of red **flowers** (1H)
*Rosa* spp. -	Rosa da giardino Ròsa	Wild-native	*FOOD:* **Flowers** are prepared in liquor (2H) and jam (1H) *COSM:* **Leaf infusion** has refreshing properties for facial skin (1P)
*Rosmarinus officinalis* L.BOLO0053712	Rosmarino Rusmarein Rusmarèn U§marén Uʃmarẹn	Cultivated	*FOOD:* It is used in cookery as flavor (3P, 35H, 19 M), and it enhances meat digestion (4H, 2 M) *MED:* **Leaves** are eaten to increase the sexual desire (1H). Smelling rosemary during the day promotes cold healing (2 M). **Leaf decoction** is used to wash genitals to cure thrush (5P), and in the case of hemorrhoids (7P), it is also used to cure skin inflammation (1P). Leaf decoction together with lemon and sage is used against gastritis (3H). Leaf decoction together with sage, lemon and devil’s grass (*Cynodon dactylon*) is used against gastritis (1H). **Leaf infusion**, drunk two times per day, calms colitis (2H), it is useful in case of migraine (1H), it is digestive (7H) and expectorant (1H). Leaf infusion together to sage prevent flu (2H). Leaf infusion (together with sage) is used as digestive (2H). Leaf infusion together with sage and mint leaves, drunk once a day for one month, is useful against arthrosis (2H). **Leaves and branches** are used in a **footbath** to remove fatigue and pain (1P, 1H). **Flower infusion** is useful against oral cavity inflammation (1H). **Leaf and flower infusion** is useful against intestinal conditions and abdominal swelling, it is recommended to drink 3 cups a day before the main meals (1 M). **Branch infusion** in warm water is drunk to treat hemorrhoids and varicose veins (1H), liver pain (1 M), coughs (7 M) and colds (7 M), to improve blood circulation (1H) and digestion (1H). **Branch decoction in wine** is used to wash and disinfect wounds (1H). **Branch macerate in wine** is useful for the liver (1H), it is diuretic (1H) and deflates belly (1H). **Branch infusion** made in **wine** is useful in case of asthma (3H), and as remedy to relieve fatigue (1 M). **Branch macerate** is used in footbath to improve blood circulation (8H); the fumes of this macerate open the lungs (2H) and heal cold (2 M, 1H). **Branch powder** is placed on wounds to promote healing (1H) *COSM:* **Rosemary wraps** are applied to oily hair to remove dandruff (2P). **Leaf infusion** is used to wash hair to strengthen it (1H). **Flower infusion** is used to strengthen the scalp (1H). **Branch infusion** in warm water is drunk in the morning to purify the skin (1H) and to make it more beautiful (2 M), and, in packs, it is anti-cellulitis (1H, 1 M) *REP:* **Branches** are put in the armchair to keep away moths (1H, 2 M). **Fresh leaves** rubbed on hands keep insects away (2H)
*Rubus idaeus* L. BOLO0034966	Lampone	Wild-native	*FOOD:* A high amount of **fresh fruits** are eaten by pregnant women to help fetal development (1 M). **Fruits** are washed and eaten fresh for their vitamin supply and pleasant taste (7 M) *MED:* **Leaf infusion** is drunk for two weeks to cure sore throat (1H) and gums inflammation (1H), it is refreshing (1H), diuretic (2 M), anti-diarrhea (1 M), anti-inflammatory of the respiratory tract (1 M) and digestive (1H)
*Rubus plicatus* Weihe & Nees BOLO0034949	More	Cultivated	*MED:* **Fruits** are eaten to regularize the intestine (1 M)
*Rubus ulmifolius* Schott BOLO0053719	Rovo Râza Ràggia Ràza Rov Râza	Wild-native	*FOOD:* **Fruits** are eaten (4H) and used to prepare sweets (2H), liquor (2H), and jam (6H), which is useful in case of cough (8H), sore throat (6H) and diarrhea (1H). The **brambles** are eaten cooked and considered beneficial for the throat (1 M) *MED:* **Aerial part decoction** is used to calm abdominal spasms (1P). **Leaf decoction** is useful against diarrhea (7H), inflamed gums (3H), and irregular menstruation (1H). **Crushed leaves** are used in **cataplasm** on plagues (3H). The external surface of the leaf put on wounds promotes the healing of the infection (1 M). Leaves together with a slice of bacon are applied on pimples (called “bugni”), promoting pus spillage (1H). An **ointment** made with **leaves** and butter is useful against hemorrhoids (3H). **Root decoction** is used as an anti-inflammatory for the oral cavity (3H) *SMR:* **Leaves** are used for the ritual of “segnatura” to heal from *herpes zoster* (called “fuoco di Sant’Antonio”): a leaf is passed over the person body while spells are cast. The ritual is repeated for several days (1H) *COSM:* **Fruit juice** with milk is an emollient and firming lotion for the skin (3H) *DOM:* The bramble, called “razze”, peeled and deprived of thorns, is used as a tie for wheat (1 M)
*Rumex acetosa* L. BOLO0003346	Vignarra Acetosa Erba brusca Êrba bróssca Erba brosca Erba forta	Wild-native	*FOOD:* **Leaves**, that have vinegar-like aroma, are eaten in salad or boiled to cure vitamin deficiency (1H), for their refreshing (1 M) and diuretic properties (3H, 2 M), and because they purify the liver (1H) and promote digestion (2 M). Children ate leaves because of their sour, tart taste (2 M). Leaves are chewed for their pleasant sour taste (1 M). **Whole plant** is useful for treating loss of appetite (2 M) *MED:* **Leaves wrap** is used to cure hemorrhoids (5H). **Leaf decoction** is used to wash the mouth in case of oral inflammation (1H), it is drunk in summer to depurate the organism (1H). **Root infusion** is useful against abdominal swelling (2H). **Leaves** are **crushed** and mixed with oil to remove calluses (10H)
*Rumex alpinus* L. BOLO0054200	Rabarbaro	Wild-native	*MED:* **Root decoction** is digestive and depurative for the liver, it is useful in case of diarrhea or fever (1 M)
*Rumex crispus* L. BOLO0052213	Romice Rumgia Rongia Lengua d’bò	Wild-native	*MED:* **Leaves** wrap is applied on bruises (2P). **Roots** are grounded and applied on eczemas (2 M)
*Ruscus aculeatus* L. BOLO0053692	Pungitopo Ponztôp Ponzôp Ponżtôp	Wild-native	*FOOD:* The **shoots** are used in cookery to prepare several dishes (1P). During the war, **seeds** were roasted and used as coffee substitutes (1 M). **Root infusion** invigorates the body (3H) *MED:* **Dried shoots decoction** (1P), and **leaf decoction** (1 M) are diuretics. **Root decoction** is diuretic (1H, 1 M), purifying (1 M), and anti-inflammatory (1 M), it cures kidney stones (5H), joint pain (2H), urinary infections (1H), and improves blood circulation (1H). It is also used to make bandages on the legs with varicose veins (1 M). **Decoction of roots** (1 M) or **roots and leaves** (1 M) is an anti-hemorrhoids remedy. **Root infusion** is antipyretic (2 M) and useful against kidney stones (1 M). Root infusion together with parsley, fennel, and celery reduces intestinal gasses (6H). **Root cream** reduces varicose veins and swelling feet (1H) *SMR:* Growing this plant in the garden keeps evil spirits away (1 M) *CRAFT:* This plant is used for Christmas ornaments (2 M) and decorations (1 M) *COSM:* **Root decoction** is used to make footbaths (1 M) and on bandages to treat cellulitis (1 M)
*Ruta graveolens* L. BOLO0032445	Ruta Rùda Rûda	Wild-native	*FOOD:* The **aerial parts** are used to flavor liquors and dishes (3P, 16H). Rue liquor is digestive (4H) and tonic (1H) *MED:* **Leaves crushed** are applied as **cataplasm** against stomachache (1P). **Leaf infusion** keeps intestinal parasites away (1H). **Leaf decoction** together with oak bark stops diarrhea (3H). **Leaf macerated in olive oil** is used in cases of muscular or joint pain and neuralgia (1H). **Leaf juice**, heated together with a bit of olive oil, is placed into ears in case of otitis or ear infections to hill and reduce earache (1H, 1 M) *SMR:* The table was covered with an odd number of **petals** (higher than 50) to have peaceful sleep and digestion (1H). The **aerial parts** were used as amulets (2P). **The plant** cultivated in the garden keeps “evil eye” away (1 M) *REP:* The **whole plant** keeps fleas and lice away (1P). The **plant** is cultivated in the garden to keep vipers away (9H, 1 M). The fresh **branches** keep mice away (1H)
*Salix alba* L. BOLO0003364	Salice Sâls	Wild-native	*MED:* **Decoction** of **branches bark** cures fever (1P). **Bark decoction** is laxative (1H), useful in case of flu (5 M), fever (2 M), pains (1 M), cold (1 M), sore throat (1 M), migraine (1 M), and menstrual pain (1 M). **Flower infusion** is sedative (3 M), and was claimed able to decrease sexual energy (1 M) *CRAFT:* Farmers make baskets and several tools for peasant life with the willow branches (3P, 4H, 1 M)
*Salix caprea* L. BOLO0003060	Salice Salicone	Wild-native	*AGROPA:* **Leaves** are used to feed goats (1 M) *VET*: **Leaves** help goats against swelling (1 M) *DOM*: **Branches** are used to tie vine (1 M)
*Salix purpurea* L. BOLO0052838	Salice rosso	Wild-native	*MED:* Thin **sticks,** held in the mouth, are a remedy for bronchitis (1 M)
*Salix* spp.	Salice	Wild-native	*MED:* **Bark infusion** is a healer (3H). **Leaf infusion** reduces inflammation, headache, and contusion pain (2H) *SMR:* **Wood sticks** are used to look for underground water by dowsers (1H)
*Salvia officinalis* L. BOLO0038758	Salvia Sælvia Sélvia Sèlvia ʃælvia Seiva	Cultivated	*FOOD:* **Leaves** are used to flavor meat (1 M), and to prepare several dishes (5P, 16H, 10 M), since they make food more digestible (9H). **Aerial parts** are used to make liquor (1H), which is done using sage, white wine, and a bit of alcohol, and this enolite has a tonic effect (1 M) *MED:* **Leaves** are disinfectant on mouth sores (3P, 3H), and are rubbed on teeth and gums to fight halitosis (5H). Leaves (5M) or aerial parts (1M) rubbed on gums have anti-inflammatory action. In case of toothache, leaves are chewed (1M). **Leaf macerate** is used to wash the mouth in case of toothache or inflammation (3H). **Leaf decoction** promotes digestion (2P, 4M), relieves stomach pain (1M), it is useful in case of diarrhea (1M), as anti-depressive (1M), to face menopause (1M), to treat menstrual cycle conditions (1M), and it reduces autumnal cold sweat (1P). Leaves decoction is also used to make rinses in case of inflamed gums (1M). Leaves decoction with lemon, rosemary, and devil’s grass (*Cynodon dactylon*) is used against gastritis (1H). Leaves decoction together with lemon and rosemary is useful against gastritis (3H), while together with parsley is useful in case of delay or lack of menstrual period (3H). **Sage decoction** is used against menstrual pains (2H). **Leaf infusion** is anti-inflammatory (1M), digestive (7H, 1M), diuretic (1M), expectorant (2M), it calms flu (1M), cough (4H, 2M), cold (4M), asthma (1H), hot flashes (1H), colitis (3H), gastritis (2H), abdominal cramps (1H), and to gargle against toothache (1H). The same infusion regularizes the menstrual cycle (1H) and is useful for menopause (1H), it reduces flatulence (1H) and sweating of hands and feet (1H). Dried leaf infusion, once cooled, is used for oral rinses for canker sores (1M, 1H), gingivitis (1M), and infections (1M). In case of toothache, leaf infusion is used to wash the mouth (2H). Sage leaf infusion together with lavender, violet, and chamomilla is useful against arthritis (1H). Infusion of sage leaves, rosemary, and mint is used against arthrosis (2H). Leaf infusion together with thyme, linden, and vervain cures headache (6H); together with rosemary treats flu (2H). A **syrup** made boiling leaves with apples and sugar is used to calm cough (1M). **Sage branches** are used to make fumes which promotes the healing of sore throat (3H) and cold (3H). Leaves have aphrodisiac power (1M) *COSM:* **Leaf macerate** together with lavender, thyme, and juniper is used on oily skin (2H). Dried leaves infusion, once cooled, is used for dry hair (1M). **Leaves** rubbed on teeth have a whitening effect (6P, 13H, 8M). Powdered leaves together with lime are rubbed on teeth to whiten them (2H). Leaves are placed in water for **footbaths** to limit sweating in the feet (1M) *SMR:* The plant protects from curse (1H)
*Salvia pratensis* L. BOLO0053688	Salvia di pre	Wild-native	*FOOD:* A precious honey is obtained from the **flowering tops.** This honey after a maceration of 20 days in brandy, is drunk as stimulating and exciting (1H) *MED:* **Leaves** are rubbed on gums to reduce sore gum (1H) and fight halitosis (1H) *COSM:* **Leaves** rubbed on teeth have a whitening effect (2H)
*Salvia sclarea* L. BOLO0029324	Salvia scarlea Sœivia seivadga	Wild-native	*FOOD:* **Leaves** are used to flavor dishes (2 M) *MED:* **Aerial part infusion** is used to treat whooping cough, called “tosse canina” (1 M), this infusion is a tonic and indicated in case of fatigue and convalescence (1 M). **Decoction** of the blue **flowers** is useful against stomachache (1 M)
*Sambucus ebulus* L. BOLO0053023	Ebbio Erba da l’udor pulèn	Wild-native	*REP:* **Flowering tops** are collected in bouquets, and due to their bad small, are used to keep lice and fleas away from chickens and from dogs (2H)
*Sambucus nigra* L. BOLO0053680	Sambuco Sambûc Sambùc Zambug Sanbûc Sambug	Wild-native	*FOOD:* **Fruits** are used to make sweet “frittelle” (17H, 5P). **Fresh fruits** are eaten as an energy source (1 M), and they are laxative (1 M). **Fresh fruits** are used to make a **jam** (22H, 3 M), endowed with depurative (5H) and laxative properties (4 M), a liquor (6H, 3 M), and a syrup, which is slimming (1 M), refreshing (1P). **Flowers** are used in cookery (5H), and are added to water to prepare a thirst-quenching drink, which is purifying and rich in vitamins (4 M). Dried flowers in tissue bags are used to flavor wine (1H). Flower jam is laxative (2 M). Flowers are macerated in water together with lemon to obtain a **s**yrup that is remineralizing (1H) and diuretic (1 M). **Flower liquor** is digestive (4P). **Fruits and flowers** are prepared in jam (15H) and liquor (3H) *MED:* **Fresh leaves** were pounded and crushed with **vinegar** and salt and collected with gauze, which is then used to treat mouth abscesses (1 M). **Boiled leaves** heal burns and wounds (6P). **Leaf ointment** is useful in case of hemorrhoids (1H). **Fresh fruits** are used to make a **juice** to treat rheumatism (1 M). **White fruit decoction** is beneficial for the respiratory tract (1 M). **Flower infusion** is febrifuge that stimulates sweating and the consequent elimination of toxins (1 M), it is drunk in case of cold (3H, 2 M), cough (2 M), asthma (1 M), urinary inflammation (2H), and to improve blood circulation (1 M). The same infusion is diuretic (2H), relaxing (4H) and reduces migraine (4H) and headache (1H). Flower infusion in **compress** is placed on wounds and burns (1H, 2 M) (it needs to be kept for at least 20 min (1 M)), and on eyes in case of stye (1H, 1 M). Flower infusion together with mint, violet and linden, treats cold (1H). **Flower decoction** is used against earache (2H) and hemorrhoids (1H). **Flowers boiled in milk** prevent gout (2H). **Flower syrup** is useful in case of cold (1H). **Flowers** are **macerated** in “grappa” to obtain an alcoholate used in rubs and massages in case of rheumatism (1H). **Flower jam** is useful in case of colds, flu and cough (2H), **Flowers and leaves** are crushed and mixed with honey to obtain a cream, which improves blood circulation of legs (2H). **Oleolite** of **branches** together with olive leaves is used against burns (17H). An **ointment** made with elder and elm bark together with olive oil is used in case of burns (2H). **Bark infusion** cures flu (2H), migraine (1 M), and it is diuretic (1 M). **Bark** soaked for 30 days in a liter of **white wine**, is drunk diluted in water, for joint pains (1 M), hands rheumatism (1 M), and cystitis (1 M). **Exudate from the bark** is collected to be applied on a contused body part (1 M) *SMR:* **Leaves** are rubbed on warts and then closed in a pot, which has to be buried, and, as soon as the leaves rot in the pot, the warts are healed (2H). An ancient legend said that whoever found an elder tree in the shadow of a willow was very lucky and had to cut some wooden circles, which became powerful talismans to be carried during dangerous journeys (1 M). According to an old peasant saying to burn elder wood was a bad omen (1 M), and its ashes invite the devil to enter the house (1 M). In making flutes, stems have to be carved at night before the cockcrow, otherwise the instrument sounds hoarse (1H) CRAFT: **Elder stems** are used to make flutes (1H, 1 M). The hoe handle is made from **elder branches** (1P). Flexible **branches** were used to replace the missing branches of the Christmas tree (1 M) *DOM:* **Fresh fruits** are used to make ink (1 M, 6H). **Clusters of white fruits** were placed in layers alternated with seasonal fruit to promote its preservation (1 M) *OUI:* **Branches** were smoked instead of cigarettes (1 M). **Black fruits** are used to fish freshwater fishes (2H)
*Sanguinaria canadensis* L. -	Sanguinaria	Cultivated	*SMR:* **The flower** is given to the loved one to make them fall in love (3P)
*Sanguisorba minor* Scop.BOLO0053687	Meloncello Pimpinella	Wild-native	*FOOD:* **Leaves** are used in cookery to prepare several dishes (2H), they are eaten in salad (1P), and promote digestion (1P, 1H)
*Santolina chamaecyparissus* L. BOLO0025375	Santolina Santuneina	Cultivated	*MED:* **Leaf infusion** promotes digestion (3H), and when applied on the skin it relieves insect bite itching (3H)
*Saponaria officinalis* L. BOLO0052459	Saponaria	Wild-native	*DOM:* Rubbed **leaves** produce a foam and for this reason, they were used to wash clothes (1 M) *COSM*: **Leaves** are used to wash hair (1 M)
*Satureja* spp. -	Santoreggia	Wild-native	*FOOD:* **Aerial parts** are used to flavor several dishes (12H) and to make a liquor (1H) *MED:* **Flower and leaf infusion** promotes intestinal gas elimination (3H), and disinfects the oral cavity (1H). **Whole plant infusion** is an expectorant (1H)
*Saxifraga* spp. -	Saxifraga	Wild-native	*MED:* Dried **flower infusion** cures cold (2 M) and cough (1 M) and in gargles cures throat inflammations (1 M), it is very effective in case of muscle cramps *SMR*: Since the plant grows on rocks it was believed that **root decoction** was able to cure kidney stones (1 M)
*Sedum acre* L. BOLO0035256	Risetto Borracina acre Saͤimpervaird Sänpervîv Êrba dal sajàtt	Wild-native	*MED* **Fresh leaves** are rubbed on the skin to relieve the itching of insect stings (7H). **Flower infusion** is a diuretic and lowers the blood pressure (3H). **Leaf juice** is used on burns to help cicatrizing, and on calluses and warts to remove them (1H)
*Sempervivum montanum* L. BOLO0050692	Semprevivo montano	Wild-native	*FOOD:* It is used in cookery added to fresh salads (1 M) *MED:* The **infusion of leaves** is used as eye drops in case of ophthalmic inflammations (1 M). **Leaf juice** is used directly on calluses (1 M) and to heal cuts (1 M) *COSM:* **Leaf juice** is emollient (1 M), and it is sprinkled on the skin to refresh it (1 M) *AGROPA:* If cows ate this plant they would go into heat (1 M) *SMR:* **Leaf juice** is given to newborns to drink as a "potion" to prolong their life (1 M) and as protection against convulsions (1 M)
*Sempervivum tectorum* L.BOLO0035192	Erba dal saètt Urciæla	Wild-native	*MED:* **Leaf juice** is rubbed on insect bites or on burns to soothe itching (1H) *SMR:* It was grown on the roof to keep lightning away (1H)
*Silene vulgaris* (Moench) Garcke BOLO0053709	Silene Strigoli Sciopetin Vverzoli Stridul Ciuchæt Ciuchaͤtt Ciuchét Striduli	Wild-native	*FOOD:* **Leaves** are used in cookery to prepare several dishes and to fill “tortelloni” (13P, 3 M). Leaves harvested before flowering (3 M) are eaten in salad (7H), because they are purifying (4H), and beneficial for the stomach (3 M) *GAME:* Kids play with the **flowers** enjoying popping them (1P, 1 M, 6H)
*Silybum marianum* (L.) Gaertn. BOLO0055362	Cardo mariano Carciofen seivadg	Wild-native	*FOOD:* **Leaves** eaten in salad are beneficial for liver (5H, 1 M) and appetite stimulant (1 M). **Peeled stems** are cooked with cheese and eggs and are considered able to purify the liver (1 M). **Raw flowers** are edible (2 M) *MED:* **Leaf decoction in compress** (1 M) is used for hemorrhoids (2H), it purifies the liver (1H), and heals canker sores (1 M). **Seed infusion** (1P) or **decoction** (1P) is purifying *OUI:* According to folk saying the whole plant is useful against *Amanita phalloide* poisoning (2H)
*Sinapis alba* L. BOLO0049472	Senape bianca Sȧṅva	Cultivated	*FOOD:* **Blooms** are eaten in salad (1P). **Young leaves** are boiled and eaten in soups, giving them a bitter taste (1 M)
*Sinapis arvensis* L. BOLO0055368	Cime di rapa selvatica Senape selvatica	Wild-native	*FOOD:* The plant is eaten in soups (1 M)
*Sisymbrium officinale* (L.) Scop. BOLO0052363	Erisimo Navåṅ salvâdg	Wild-native	*MED:* **Flower and leaf infusion** is useful in case of hoarseness, cough (2H, 1 M), and diarrhea (1 M)
*Solanum dulcamara* L. BOLO0049337	Dulcamara Erba di sérp	Wild-native	*MED*: **Branch decoction** is laxative (1H) *COSM:* The **juice** of red and ripe **berries** is a skin-whitening agent, useful to reduce age-related skin spots and freckles (1 M)
*Solanum lycopersicum* L. BOLO0052805	Pomodoro Pandòr Pûndor	Cultivated	*MED*: An **ointment** made with tomatoes and pig fat is used to cure hemorrhoids (2H). A **slice of tomato** is put on the burns to reduce pain (6H) *REP:* **Tomato plant** keeps insects and flies away (2H)
*Solanum melongena* L. -	Melanzana Melanzèna	Cultivated	*FOOD:* **Fruits** are used in cookery (8H) *MED*: **Fruits** are cut into slices which are covered with salt and used in a wrap to cure burns and skin inflammations (5H)
*Solanum tuberosum* L. BOLO0015488	Patata Patæda Patæga Pated Paten Patèta	Cultivated	*FOOD:* Potatoes are eaten because they are restorative (11H) and have diuretic properties (1 M). Potatoes are eaten in salads (3 M) *MED*: A **slice of potato** deflates the face (1P) and eyes (8H), heals toothache (1 M), and is useful in case of burns (17H, 7 M) and to relieve skin inflammation (1H, 1 M). **Grated tuber** is applied on swollen eyes (5P, 1 M), it is clenched between the teeth in case of oral inflammation (3P), and it is applied on the skin to relieve burns (3H). Potatoes together with onion reduce abscess swelling (1H) *AGROPA:* Pieces of several plants were inserted into potatoes in order to preserve them before grafting (1 M). To rub half potato on cut branches to protect them from bacterial or fungi infections (2 M). Unmarketable potatoes are given to pigs (1P)
*Soldanella alpina* L. BOLO0003036	Soldanella	Wild-native	*MED*: **Roots infusions** have laxative properties (1 M)
*Sonchus* spp. -	Radicchio di campo Crispigni Stricapògn Strécpogn Cudres Zrèvda Brég ed gal	Wild-native	*FOOD:* **Leaves and roots** are eaten in salads (17 M) or boiled for their digestive properties (16 M). **Leaves** are eaten in salad (6P, 11H, 7 M) or cooked with bacon (5H), to promote intestinal transit (6P, 7H), clean the liver (1H, 5 M) (especially basal tender leaves (3 M)), they are purifying (8H, 10 M), digestive (6 M), and they are good for the heart (2P). During the war, the **roots** were roasted and used as a coffee substitute (1 M) *MED*: **Leaf decoction** is used to disinfect the airways (2H) and in case of liver conditions (1H)
*Sorbus aucuparia* L. BOLO0054055	Sorbo degli uccellatori	Wild-native	*MED*: **Fruits** are laxative (2H) and promote digestion (2H) *SMR:* It was believed that eating 7 fruits all together at 7 p.m. would have generated an instant laxative effect (1H)
*Sorbus domestica* L. BOLO0055357	Sorbo Sòrbel	Cultivated	*FOOD:* Although sour, the **fruits** are eaten (1H), because they are rich in vitamins (4 M). Fruits, ripen under the chaff and are used to prepare a jam (1H) *MED*: Fresh **fruits** are eaten to stop diarrhea (1H), dried **fruit infusion** boiled in water is useful in case of diarrhea (1H, 2 M). **Fruit decoction** is used against phlegm (1P) and as digestive (1P) *COSM:* **Dried fruits** are **boiled**, and the water, once cooled, is used to make **packs** on the face to reduce redness (1 M), and used as a detergent to prevent wrinkles and early aging of the skin (1 M) *CRAFT:* The part of the **trunk** near the root was used to make hammers because the wood is very hard and resistant (1 M)
*Sorbus torminalis* (L.) Crantz BOLO0046857	Ciavardello	Cultivated	*FOOD:* **Fresh fruits** are eaten as they are rich in vitamins (1 M) and they are prepared in jam (2 M) and liquor (1 M) *MED*: **Bark infusion** is anti-diarrheal (2 M) and useful in cases of colic (2 M) *CRAFT:* The **wood** was used to make musical instruments (1 M)
*Sorghum bicolor* (L.) Moench BOLO0015581	Saggina Melga	Cultivated	*CRAFT:* A broom is made with the sorghum **inflorescences** (1P)
*Spartium junceum* L. BOLO0002909	Ginestra odorosa Ʒnœstra	Wild-native	*MED*: **Flower infusion** is diuretic (1H). **Flower jam** cures sore throat (1H) *COSM:* **Flower jam** is useful to lighten the hair (1H) *CRAFT:* **Whole plant** is used to craft ropes (1H)
*Spinacia oleracea* L. BOLO0015356	Spinacio	Cultivated	*FOOD:* **Leaves** are eaten in salad (4P) or are used to make fresh pasta (1P). To eat the leaves gives strength to muscles and bones (4P)
*Stachys officinalis* (L.) Trevis BOLO0052478	Betonica comune Erba bertonga	Wild-native	*MED:* **Fresh leaves** are used as wound healers (1H)
*Stachys recta* L. BOLO0049498	Stachys Strigonella Erba bona Erba d’la pòra Êrba d'la pôra Êrba da la pôra	Wild-native	*MED*: **Decoction in compress** relieves eye pain (1 M). **Whole plant infusion** is used externally because it has relaxing properties (2H) *SMR:* **Fresh plant juice** is used in a ritual, called “segnatura”, performed to exorcize the fear due to trauma (2 M). The plant is harvested on the Night of S. Johan (23th of June), bunches are dried, and extracted in water as a **decoction** used in a ritual to wash away the fear, by praying or casting a spell. If the liquid is getting turbid it means that the ritual needs to be repeated the next day until the liquid will stay limpid (1 M). **Whole plant** (6H) **decoction** (15H) is used to exorcize fear
*Stevia rebaudiana* Bertoni -	Stevia	Purchased product	*FOOD:* **Dried leaves** are used as a sweetening agent (2H)
*Sulla coronaria* (L.) BOLO0003071	Sulla	Wild-native	*FOOD:* **Leaves** are used in salad or to prepare several dishes (1H). The “sulla honey” is laxative (1H), tonic (1H), and throat emollient (1H) *MED*: **Whole plant infusion** prevents intestinal infections (1H), it is used against stomachache (1H) and diarrhea (1H) *AGROPA:* **Areal parts** are used as feed for animals (3H)
*Symphytum officinale* L. BOLO0049110	Consolida maggiore Êrba d San Luränz	Wild-native	*FOOD:* **Leaves** are used to prepare a delicious herbal tea (1H), **roots** as a sweetener (1H), and **shoots** are used in cookery (1H). **Root** soup is remineralizing (1H) *MED*: **Fresh leaf** (1H) **or root decoction** (1H) is used in **cataplasm** to heal wounds and burns. **The pulverized root** is useful against sore throat and as an expectorant (1H). **Leaves and flower infusion** calms cough (1H). **Dried plant** is crushed and used to relieve pain due to wounds (1H) *AGROPA:* **Leaf macerate** is used as soil fertilizer (3H)
*Syringa vulgaris* L. BOLO0007236	Serenella	Wild-alien	*MED*: **Aerial part infusion** reduces gastrointestinal inflammation (3H) *DOM:* **Flowers** are ornamental (1H)
*Syzygium aromaticum* (L.) Merr. & L.M.Perry BOLO0010580	Chiodi di garofano Ciûd ed garòfen Ciôd ed garôfen	Purchased product	*FOOD:* **Cloves** are used in cookery (11H), and to prepare mulled wine (2H) *MED*: **Cloves** help digestion (1H). Clove applied on the tooth calms toothache (9H, 2 M). **Clove infusion** was used to gargle in case of sore throat (2H, 2 M). **Cloves decoction** with honey and milk is drunk in case of cough (1 M) and cold (1 M). **Flower infusion** is used to wash the mouth in case of toothache (4H) *DOM:* **Flowers perfume** the indoor (10H) *Repellent & Insecticide:* **Flowers perfume** is anti-moths (12H)
*Tanacetum balsamita* L. BOLO0013288	Balsamita Balsamite odorosa Menta romana Èrba d'la Madona Èrba ed Santa Maria Santa maria Erba dla Madòna Êrba dla Madòna	Wild-alien	*FOOD:* **Leaves** are edible, they have a bitter taste (2H) *MED*: **Seeds** are eaten to prevent intestinal parasites (3H). The plant is crushed and used in **wraps** to strengthen the stomach (1H). **Leaf boiling water** is used for emollient baths in case of reddish (1 M) *COSM:* **Leaves** are placed by the women near the chest, as a perfume (1H)
*Tanacetum corymbosum* (L.) Sch.Bip. BOLO0025388	Tanacetum	Wild-native	*MED:* **Leaves** have a balsamic action (2H) *DOM:* **Leaves** are used to perfume clothes in the closet (2H)
*Tanacetum parthenium* (L.) Sch.Bip. BOLO0052352	Artemisia Partenio	Wild-native	*MED*: **Flower infusion** relieves headache (2H). **Aerial part infusion** reduces cephalalgia (1H) and menstrual symptoms (1H)
*Taraxacum* spp.	Tarassaco Soffione Dente di Leone Pessalet Pesàlet Pessalèt Pessàlæt Streccapóggn Pessalèt Supiån Lattuga di cane Corona di monaco	Wild-alien	*FOOD:* **Leaves** (harvested before flowering (1 M)) are eaten raw in salad (9P, 2H, 15 M) or boiled (1H), since they promote digestion (6H), are diuretic (19H), refreshing (1H), depurative (2 M, 5H) for liver (9H, 3P), and lower blood pressure (16H). Leaves are eaten with bread and eggs since they are rich in minerals and have a depurative (2 M) effect, but are slightly laxative (2 M). Leaves are rich in iron (8H). **Young leaves** are the best for depurative salads (1 M, 7H), together with apples, cheese and walnut oil (1H). **Stems** are boiled and eaten in salad (23 M). **Aerial parts** have diuretic (3P) and hepatic protective proprieties (3P, 1 M). **Flowers** (**buds** 3 M) are used in cookery (5H), they are eaten in salads or in soups (3H); before blossoming are placed in vinegar to make preserves (1H). **Flower jam** is beneficial for the troth (1H). **Roots** are roasted and used to prepare a coffee substitute beverage (1P, 1H) *MED*: **Leaf decoction** is thirst-quenching, it is useful against urinary tract inflammations (2H), and is a diuretic (19 M). **Leaf boiling water** is drunk to purify the liver (12 M). **Leaf infusion** is bladder refreshing (1H), purifies the liver (1H, 1 M), it has refreshing properties (1H), and lowers blood pressure (1H). **Flowers** macerated in sugar and boiled give a product similar to honey to be used as **syrup** to heal cough or sore throat (5 M). **Flower decoction** is a diuretic (1 M). **Aerial parts decoction** is diuretic, and useful for kidney conditions (2P, 1H) and to purify the liver (2P). **Root infusion** is diuretic and digestive (1 M), and is beneficial for the kidney (1H). **Root decoction** is used as tonic in case of muscle weakness (1H), it is depurative for the liver (2H, 2 M) and for the organism (1H, 1 M), it helps digestion (1 M), stimulates kidney function (1 M), it treats liver conditions if it is taken once a day (1H), it helps to lose weight (1H) it is diuretic and draining (6H, 3 M), and it is recommended to drink this decoction during seasonal changes (1H). **Whole plant** is used in a bath for varicose veins (3 M). **Whole plant infusion** purifies the liver (3H), is a diuretic (2H), and is used against pimples (2H) *COSM:* **Roots** are **boiled** for half an hour in water and left in infusion for two hours obtaining water used for rejuvenating face skin (1 M). **Fresh plant juice** is rubbed on the face to lighten freckles (1 M, 1H). **Root decoction** reduces cellulitis (1H) *SMR:* **The pappus** is blown to bring good luck (16H). The number of times a girl had to blow to disperse all the elements of the pappus corresponds to the number of years she had to wait before marriage (1H). It is possible to predict the weather by the pappus, if there are flying fruits on a non-windy day, it means it will rain (1H) *OUI:* Saying that somebody “eats the tarassaco from the root” was a metaphoric expression used to indicate that this person was dead (1H)
*Taxus baccata* L. BOLO0054005	Tasso	Wild-native	*CRAFT:* The **wood** is used to make pipes (1H)
*Teucrium chamaedrys* L. BOLO0049499	Camedrio Querzò Êrba quarzôla Erba querciola Erba quarzola	Wild-native	*FOOD:* **Leaves** are used to prepare a digestive (1 M) liquor (1H) *MED*: **Flowering top infusion** is laxative (1H)
*Thymus vulgaris* subsp. *vulgaris* BOLO0046719	Timo Temm	Wild-native	*FOOD:* **Leaves** (1P, 7H, 4 M) or **flowers** (1H) flavor several dishes. Leaves or whole plant are used in cookery and to flavor liquors (6H) *MED*: **Sprigs** are rubbed to disinfect hands (1 M). **Leaf infusion** promotes digestion (2P), has expectorant proprieties (7H), calms cough (2H) and is used against halitosis (1H), flatulence, and swelling (1 M). Leaves infusion together with sage, linden, and vervain is anti-headache (6H). Leaves infusion with mint, linden, yarrow, and honey is used as pimple treatment (7H). **Leaves decocted** together with “crescione” (*Nasturtium officinale*) strengthen the vocal cords (1 M) and it is vermifuge (1 M). **Aerial parts infusion** is used to wash and disinfect the mouth (1 M), and in case of bronchitis as expectorant (2 M). Aerial parts (including top flowering) **decoction**, drunk several times per day, heals whooping cough and sinusitis (1 M). **Flower infusion** is used in case of stomachache (2H), it calms cough (4H), reduces inflammations of the first respiratory tract (3H), and is useful for gastrointestinal inflammations (3H). **Flower decoction** disinfects the inflamed oral cavity (6H). **Boiled fruits** are used in **wrap** on the thorax in case of fever (2H) or bronchopneumonia (3H) *COSM:* **Leaves macerated** together with lavender, sage, and juniper are used in facial masks to treat fat skin (2H). **Whole plant decoction** is used to wash hair to strengthen and soften it (1 M)
*Tilia cordata* Mill. BOLO0001177	Tiglio	Wild-native	*MED*: **Flower buds** and the small leaf near the flower, collected around June-July, are dried in the shade to prepare a **decoction** useful to cure cough and sore throats (1 M). **Flower decoction**, together with mint leaves, is a remedy for cold and sore throat (1 M). **Flower infusion** is drunk every morning as a sedative (3 M), it has beneficial properties for the heart (1 M) and for blood circulation (1 M). Flower infusion with laurel berries is anti-headache (1 M)
*Tilia platyphyllos* Scop.BOLO0052804	Tiglio Télli Téli Teli	Wild-native	*MED*: **Flower infusion** is useful in case of skin rash (1H), cold (1P, 1 M), cough (1P, 1H), and it is expectorant (1H). It calms belly and intestinal pain (5H), headaches (2 M), nervousness (1 M), tachycardia (1 M), and anxiety attacks (2 M), and it is relaxing (8P, 20H), in fact, it is added to water in order to make relaxing baths (1 M). It promotes sleep (7P, 2H, 2 M), it is useful to calm heartburn (1P), and it induces sweating (1H, 1 M). The water obtained from flower infusion is used in bandages applied on burns (1 M) and erythema (1 M). Flower infusion with mint, violent, and elder is used against colds (1H). **Leaf infusion** is used against sore throat (3H). Leaves infusion together with sage, thyme, and vervain is useful against headache (6H). Leaves infusion together with mint, thyme, yarrow, and honey is used as pimples treatment (7H) *COSM:* **Flower infusion** is used to make eye packs able to remove dark circles (1 M), and it is useful for refreshing rinses (1 M), for purifying and rejuvenating the facial skin (1H), and attenuating facial redness (1 M). **Dried leaves** of linden and sage are rubbed on tooths as a whitening agent (2H) *CRAFT:* **Wood** was used to produce furniture (1 M) *AGROPA:* **Flowers** attract bees which make honey (8H, 1 M)
*Tilia* spp.	Tiglio	Wild-native	*MED*: **Flower infusion** reduces anxiety and insomnia because it is sedative (5H), treats tachycardia (4H) and stomachache by helping digestion (2H), it is antitussive (9H) and it is used against sore throat, and cold (9H) *COSM:* **Flowers** in hot water are used for scented foot baths (1H)
*Tragopogon pratensis* L.BOLO0048815	Barba di becco Barba d’frè	Wild-native	*FOOD:* **Leaves** are eaten in salad (2P) since they supply minerals (1P) and purify the blood (2P)
*Trifolium pratense* L. BOLO0002900	Trifoglio di campo Trifòi Tarfòi Trafojj	Wild-native	*MED*: **Whole plant infusion** is useful against menopause disorders (5H). **Aerial parts** are **macerated in vinegar** to cure ingrown nails (2H) *SMR:* A **clover** with four leaves brings good luck (8H) *AGROPA:* **Seeds** are used to inhibit the spread of weeds in the field (3H)
*Trifolium repens* L. BOLO0048773	Quadrifoglio	Wild-native	*SMR:* A **four-leaf clover** brings good luck to those who harvest it or to those who get it as a present (1H)
*Trigonella foenum-graecum* L. BOLO0014474	Fieno greco	Cultivated	*MED*: **Fresh leaves** treat pimples (2H). **Seed infusion** calms cough (2H)
*Triticum aestivum* L. BOLO0018446	Grano Gran	Cultivated	*MED*: Two spoons of **flour** are added to a cup of water and drunk when the flour sediments on the cup bottom; this water has detoxifying, depurative, and anti-inflammatory properties for the intestine (1 M) and urinary tract (1 M) *AGROPA:* A cow bed is made with straw (1P), and cows are fed with the straw since it has a digestive action (1P) *DOM:* Paper is made with the stems (1P) *CRAFT:* Bags are made with the stems (1P)
*Triticum* spp.	Frumento Furmæint Furmeint Furmänt Grano Grên		*FOOD:* Widely cultivated and used in cookery as a famine food (1 M), it is used to make flour for bread (1 M), and to prepare “frittelle” (called “mangnaza”) dipped in wine, which gives energy and are beneficial for stomach and intestine (1 M) *MED*: The bran **wraps** are put on the chest and used to cure bronchitis (2P, 4H). This plant is used to make **fumigation** in case of cold (1 M). Warmed bran is applied on the chest in case of bronchitis (2H). Bran mix is laxative if it is taken with water (9H), and cures intestinal conditions (1H). A **warm bath** with flour removes cradle cap (2H) *VET:* Bran, called “rémmel”, obtained from wheat grains and mixed with water is laxative for cattle (1 M) *DOM:* **Dried stems** are burned to cook bread (1H). Eggs and aged cheeses are kept between the kernels stored in chests (1 M)
*Tropaeolum majus* L. -	Nasturzio	Cultivated	*FOOD:* **Blooms** are used as caper substitute (1P)
*Tussilago farfara* L. BOLO0052350	Tossilaggine Farfalle Farfaræia Farfaræla Farfaréla Piedunaza Farfarœla	Wild-native	*FOOD:* **Leaves** are eaten raw or cooked in salad (1 M) *MED*: **Flower infusion** relieves cough (8P, 5H, 2 M), asthma (1 M), and migraine (1 M), it cures bronchitis (1H), fluidizes catarrh (2H), and cures a common cold (1P, 1 M). **Leaves** are applied on *Herpes zoster* sores (1 M). **Leaf decoction** is an expectorant (1 M). **Leaf pulp in cataplasm** cures abscesses (1H). **Fumes of leaves** (1H) **and roots** are beneficial for asthmatics (2H). **Flowers** were rolled and smoked as a cold remedy (1 M) *COSM:* Externally, **flower infusion** is used to soften skin (1 M) *AGROPA*: **Leaves** are used to feed pigs (1 M), and cattle since they have a fortifying effect (5H) *OUI:* Leaves were used like glasses to drink water in the mountains (1 M). **Flowers** were added to tobacco in pipe (2 M)
*Ulmus minor* Mill.BOLO0052995	Olmo Oilm Aulum	Wild-native	*MED*: **Bark powder** is useful to treat skin diseases (1H). An **ointment** made with the **bark** and olive oil is used on burns (2H), to treat skin diseases (1H), and to promote wound healing (1H). **Bark decoction** has healing properties (1H), and it is used to purify skin and reduce acne and eczema (1H) *CRAFT:* **Wood** is used to make the yoke for the cattle (1H) *OUI:* Folks used to set up a court of justice and hang under an elm tree (1H)
*Ulmus* spp. -	Olmo	Wild-native	*MED*: **Bark** together with pig fat is heated in a bain-marie to obtain an **ointment** with wound healing activity (1H) *AGROPA:* **Leaves** are used to feed animals (1P), and to support the vine (1P) *CRAFT:* **Wood** is used in carpentry (1P) *DOM:* **Wood** is used as firewood (1P)
*Urtica dioica* subsp. *dioica* BOLO0055354	Ortica Urtîga Urtiga	Wild-native	*FOOD:* **Boiled leaves** are eaten for their purifying properties (1 M), because they are rich in minerals (4H), and it is recommended to eat them during breastfeeding (1 M). Leaves are used to prepare several dishes and to make fresh pasta (29P, 80H, 64 M). Leaf infusion is drunk since it is a nutrient and thirst-quencher (4H, 1 M) *MED*: **A nettle bunch** beaten on a leg is helpful to activate blood circulation (4P, 4H), or to relieve pain (3P). **Fresh plant** (4 M) (**or fresh plant juice** (1 M)) is rubbed on the painful body parts to reduce rheumatism, or for muscular pain (3H), this treatment has to be prolonged for one week (5H). **Leaf juice** with honey is used to cure hemorrhoids (2H), while mixed with olive oil is used as a lotion able to activate circulation and is useful against chilblains (1 M). Leaf juice is used in the case of nosebleeds (1H), while together with olive oil and salt it is used in the case of chilblains (1H). **Fresh leaves** are rubbed on wrists to cure bone pain (1H), and on sore body parts in case of back pain and arthritis (2 M). Leaves are used to treat ingrown toenails (1 M). **Powder leaves** are sniffed in case of nosebleeds (2H). **Leaf decoction** is used in gargling against throat inflammation (1H), in **warps** it stops hemorrhages (2H) and relieves joint pain (1H), it has diuretic action (1P, 12 M), induces menstruation (1H), cures intestinal inflammations and diarrhea (2 M), and it is liver depurative (4 M). The same decoction is used against impotence (1H). **Boiled leaves in wrap** are useful against pimples (1H). Boiled leaves are used for massages to treat rheumatism (3 M). **Leaves boiled in wine** are diuretic (1 M). **Leaves infusion** purifies the blood (2H) and the body (1H, 1 M), it is slightly laxative (6H), and it is used as a tonic (1H). **Whole plant infusion** is useful against diarrhea (5H). **Aerial part infusion** is a diuretic (1H) and is used in case of stomachache (1H), diarrhea, and enteritis (1H). **Aerial part decoction** yields a purple liquid, that stops hemorrhages (1 M), and mixed with honey and used to calm hemorrhoid pain (1 M), and calms urticaria rush (1 M). **Leaf macerated in alcohol** is used in case of diarrhea (2H). **Roots** powder is boiled with sugar to make a **syrup** useful in case of cough (1H) *COSM:* **Fresh leaves** are rubbed on the scalp to reduce dandruff (1 M, 2H). **Boiled leaves** are chopped and used to obtain an anti-dandruff **ointment** (2H). **Leaf decoction** is indicated for oily hair (1 M, 3H), it is used to wash the hair (5 M, 8H), is used to counteract hair loss (7 M, 1H) and dandruff (5H, 1P, 8 M), to strengthen it (1P, 3H) and to make it shiner (2H, 2 M). Leaf decoction together with **vinegar** strengthens hair (1H) and reduces dandruff (1H). **Leaf infusion** reduces hair loss, hair strengthens (9H), and makes it shinier (3H). **Leaves** are **macerated** in denatured alcohol for 40 days and used on the scalp as a remedy for hair loss (1 M). Leaves macerated in wine are used in wraps on the scalp and, with water, to wash hair (1 M). Leaves macerated together with rosemary and aquavit reduce hair loss (1H). **Roots** are **macerated in alcohol** to prepare a lotion against hair loss (1H) *SMR:* According to a folk saying, nettle leaves do not sting if collected while holding your breath (1H). Legends say that rolling on a field of nettle increases sexual energy (1 M), occasional pricking prolonged life (1 M), and throwing nettle in the fire drove away thunderbolts (1 M, 1H) *AGROPA:* Nettle is used to feed chickens (2H), in particular, **fresh leaves** are mixed with corn flour to prepare hence feed, which increases egg production (1P), and the eggs of the hens fed with nettle are more nutrient and reddish (1H, 2 M). Chickens are fed with fresh leaves and corn flour during weaning (4H). **Leaf infusion** is given to livestock since it is a nutrient and thirst-quencher (1 M). **Leaf water macerate** (with garlic leaves (1H)) for 24 h is used after one week as fertilizer (3H, 2 M) *REP:* **Leaf macerate** in water for 24 h (with garlic leaves (2H)) is an insecticide (3H) and anti-aphids (1H, 2 M). Leaf macerate in water for 30 days is used as insecticide in particular for tomato plants (5H). **Plant macerate in water** is used to water the vegetable garden to keep parasites away (1H). **Flowers** are **extracted in water** for few days and sprayed on ornamental plants as anti-aphids (1 M). **Plants** are put next to windows in order to keep away small insects and mosquitoes (2H) *DOM:* **Leaves** are used, along with water and sand, as a detergent to clean bottles (1 M) *OUI:* **Stems** were used to make a very resistant fabric during the First World War (1H)
*Vaccinium myrtillus* L. BOLO0054059	Mirtillo Baggiôli Martél Mirtéll	Wild-native	*FOOD:* **Fruits** are harvested and eaten fresh for their taste and vitamin content (6 M), and because they are beneficial for the sight (7 M). Fruits are used to make jam (7H), sweets (4H), and liquor (1H), in particular, ripened cones are macerated in “grappa” for 20 days to obtain a “grappa” with blueberry flavor (4 M), which is digestive (2 M) *MED*: **Whole plant infusion** improves blood circulation (5H), and eyesight (5H), and it disinfects the urinary tract (1H). **Fresh fruits** improve intestinal functions (4 M) and relieve liver conditions (2 M). Fruits have beneficial properties on sight (9H, 2 M), skin (1 M), circulation (1 M), hemorrhoids and blood microcirculation (3H), and urinary infections (1 M). **Fruit juice** improves eyesight (1H), it protects blood vessels (1 M) and it is useful in case of diarrhea (1 M). **Fruit alcohol macerate** is used in case of mouth inflammation (2H) or to fight enteritis (1H). **Fruit decoction** is useful against colitis (3H), it is astringent (1H), and useful against diarrhea (1H). It is also used to wash the face in case of skin disease (1H). Fruit decoction together with oak bark and mallow leaves is used in **wrap** in case of hemorrhoids (2H) *DOM:* **Fruit juice** is used as ink (1H)
*Vaccinium uliginosum* L. BOLO0046883	Mirtillo chiaro	Wild-native	*TOXIC:* It differs from *V. myrtillus* for the leaves and the fruits, which have a lighter color. It is important to be able to distinguish between these two plants because *V. uliginosum* is toxic and it is a powerful laxative (1 M)
*Vaccinium vitis-idaea* L. BOLO0005034	Mirtillo rosso	Wild-native	*FOOD:* **Fruits** are used to make sweets (6H) *MED*: **Fruits** cure urinary infections (3H), and improve blood circulation and hemorrhoids (3H)
*Valeriana officinalis* L. BOLO0049319	Valeriana Valeriena Valerièna Grassa gallina Valeriæna	Wild-native	*FOOD:* **Leaves** (especially young leaves (1 M)) are eaten in salad (5H), they are purifying (2P), and beneficial for the heart (3P) *MED*: **Aerial part infusion** has a sedative effect (1P). **Aerial part decoction** is febrifuge (1 M). **Flowers extracted in water** for one night calm anxiety (1 M). **Root decoction** is a sedative, anti-stress, and promotes sleep (15H, 3 M), it is relaxing (1 M), and it helps to focus and strengthen memory (1H). **Root macerate** is drunk in case of asthma (2H). **Leaf and root infusion** is sedative (3H) *COSM:* **Flowers extracted in water** for one night, **in compress** is anti-cellulitis (1 M) *OUI:* For the peculiar smell of this plant, cats roll around it (1 M). **Roots** when harvested smell like cat pee (1H)
*Valerianella locusta* L. BOLO0053699	Velerianella Formentino Grassa galèna Galèna grasa Grâsagalenna Grâsagaleṅna	Wild-native	*FOOD:* **Leaves** are eaten in salad (3P, 3H) since they purify the liver (1P, 5H), are rich in minerals (5H), promote fat-burning processes (1P), and raise body temperature (3H)
*Verbascum thapsus* L. BOLO0049225	Verbasco Tàs bardâs Tâs bârdas Tâs Bardâs	Wild-native	*FOOD:* **Leaves** are used to preserve the dry figs from rotting (1 M) *MED*: **Flower infusion** is used in case of cough and catarrh (1H), and fever (2H). **Leaf and flower decoction** is emollient, protective, and anti-inflammatory in the case of hemorrhoids (2 M). **Flower** mixed with eggs, breadcrumbs, and boiled leek leaves is used to soothe inflamed hemorrhoids (1 M) *DOM:* **Stems** are used to power the wood oven to cook the bread (1 M). This plant is also called “candelabro” (candlestick) since dry and bent leaves are used as wicks to oil lamps (1 M)
*Verbena officinalis* L. BOLO0052765	Verbena Verbêna	Wild-native	*FOOD:* **Upper aerial parts** are eaten (raw or boiled) in the salad because they are purifying (1H), and stimulate appetite (1H). **The whole plant** is used to prepare a liquor (2H) *MED*: **Flowering top infusion** is digestive (4H). **Flower, leaf, or whole plant decoction** relieves bone pain (1H), is useful against headache (1H), and lowers the temperature in case of fever (1H). **Flower and leaves decoction** is febrifuge and it has to be drunk 1–2 times per day (2 M). **Leaf wrap** absorbs bruises and hematomas (2H). **Leaf infusion** is useful against rheumatic pain (1H). Leaf infusion together with sage, thyme, and linden is useful to relieve headache (6H) *DOM:* **Flowers** are ornamental (3H)
*Veronica officinalis* L. BOLO0049439	Veronica Occhi della madonna Verônica	Wild-native	*MED*: **Flowering aerial part infusion** is refreshing (1H), digestive (1H), and useful against cough (1H). **Leaf pulp** is applied on pimples (1H)
*Vicia faba* L. -	Fava Fèv	Cultivated	*FOOD:* The **fruits** were a substitute of meat during the war (1P) *MED*: The **broad bean pulp** in vinegar cures burns and bruises (1P) *AGROPA:* **Broad bean pod** is used to feed animals (1P). **The plant** is cultivated since it enriches the soil for the subsequent crops (2H)
*Vinca minor* L. BOLO0003318	Pervinca Viôla mâta	Wild-native	*MED*: **Leaf decoction** is useful against sore throat (2H). **Leaf infusion** is a galactagogue (4H), stops hemorrhages (1H), heals wounds (1H), and stops vaginal discharge (1H). **Root decoction** is diuretic (2H)
*Viola odorata* L. BOLO0003310	Viola Viôla Viôla zôpa Viôla zôpa	Wild-native	*FOOD:* **Flowers** are used to prepare several dishes (4P) *MED*: **Flowers**, slithered on the eyelids, help to keep the sight healthy (1P, 1H, 2 M) and prevent blindness (2H). **Flower infusion** is used in case of cold (2H). Flower infusion together with mint, linden, and elder is used against cold (1H). Flower infusion together with lavender, sage, and chamomilla is used in case of arthritis (1H). **Root and flower infusion** is useful against cough (2H) and cold (4H). **Leaf infusion and decoction** are laxative (4H). **Aerial part decoction** is effective in treating the cracking of hands (1 M)
*Viola tricolor* L.BOLO0047101	Violette	Wild-native	*MED*: **Leaves and flowers**, harvested in spring, are used to prepare purifying **herbal teas** (1 M). **Flowering aerial parts** are used to prepare an **ointment** for skin affections such as acne, eczema, and hives (3H) *DOM:* **Flowers** are harvested and used as scenting agents (1 M)
*Viscum album* L. BOLO0047421	Vischio Vessti	Wild-native	*MED*: **Leaf infusion** lowers blood pressure (2 M), it is effective against abdominal pain (1 M), and it is diuretic (1 M). Leaves infusion together with sugar cures kids’ seizures (2H). **Cleaned branch infusion** is diuretic (1 M) *DOM:* In folk traditions, it was used as an ornament for the house. It is possible to find *Viscum album* on chestnut tree, since is its parasite (3 M)
*Vitis labrusca* L. BOLO0001714	Uva Fragola	Cultivated	*FOOD:* Eating **fresh fruits** helps digestion (1 M), and metabolism (1 M), they are rich in vitamins (3 M) and mineral salts (2 M) *DOM:* Growing on arbors it is useful to make shade in the garden (1 M)
*Vitis vinifera* L BOLO0052747	Vite rossa Vîd	Cultivated	*FOOD:* **Grape must** together with flour is used to prepare traditional desserts (called “sughi” which are similar to pudding) (1P, 15H, 2 M). Grape must together with apples and pears is used to make a traditional jam called “*savåur*” (4H). From the **wine**, the vinegar is useful to season food (2H, 1 M) *MED*: **Wine** is recommended for the anemic and cardiopathic (1H, 1 M). Drinking a glass of wine is healthy for the stomach and intestine (1 M) and it is a tonic for the elderly and infirm (1 M). **Vinegar** is useful to disinfect small wounds (8H, 1 M). A teaspoon of vinegar relieves inflamed throat and melts catarrh (8H). Vinegar is used to wash the oral cavity in case of sore throat (6H, 1 M). **Mulled wine** is useful against colds, seasonal ills (1H) and coughs (3H). Vinegar is smelled to recover after fainting (4H). Branches are cut, and the released water is used as eye drops to wash and refresh eyes (1H). Drops of water, fallen after the pruning, are useful against juvenile pimples (1H). **Leaf infusion** improves blood microcirculation of the hand, feet, and legs (1H). **Leaves** are used to make an **ointment** useful in case of hemorrhoids (1H) *COSM:* **Vinegar** is used to wash the hair, making it more beautiful (1H) *SMR:* Three branches with leaves are used to rub painful body parts three times, hence after this procedure the branches have to be thrown first right, then left, and back Only in this way, the pain will stop in a short time (1H) *DOM:* **Grape pomaces** are used as fuel (1P)
*Zea mays* L. BOLO0015578	Mais Furmintan Furmintån	Cultivated	*FOOD:* It was widely cultivated in times of poverty and used to make “polenta” (3 M) *MED*: **Stigma infusion** stimulates diuresis (3H) and purifies the liver (1H). A spoon of **maize** in water was used to make a diuretic **decoction** (1 M), to cure kidney stones (2 M) and cystitis (2 M) *CRAFT:* **Leaves** are used to make bags and stuffing mattresses (2P) *DOM:* The **cob** was used as fuel for the stove (1P). **Leaves** are used to keep cool the bottle of wine (1P) *AGROPA:* **Fruits** are used to feed hens (1P) and farm animals (2 M)
*Zingiber officinale* Roscoe BOLO0008694	Zenzero	Cultivated	*FOOD:* **Rhizome** is used in cookery to flavor several dishes such as soups (6H) *MED*: **Rhizome infusion** is used to regularize body temperature in case of fever (1H)
*Ziziphus jujuba* Mill. BOLO0055385	Giuggiolo	Cultivated	*MED*: Fruit decoction is useful against cough (1P) and sore throat (1P)

For each taxa are reported: scientific name and Voucher specimens, common name(s) (in the dialect of Bologna which can have minoritarian inflection variations), status, and traditional uses, specifying which plant part is used and the preparation done (highlighted in bold), while in brackets, it is reported the number of citations for each use, together with the area where the interview was carried out (H = hill, M = mountain, P = plain). The traditional uses have been divided in the twelve macro-categories (UC): medicinal (MED), food (FOOD), cosmetic (COSM), domestic (DOM), superstitious-magical-religious (SMR), agropastoral (AGROPA), craft (CRAFT), repellent-insecticide (REP), veterinary (VET), toxic (TOXIC), games (GAME), other uses & information (OUI)

Once finished all the interviews, the information was merged as reported in Table 2, and the data were schematized in an Excel matrix, reporting in rows the taxa and in the columns specific use categories and related subcategories, citation number, plant preparation, and used organ. This data matrix was then organized in pivot tables to easily access the information and to obtain all the graphs. The work done in Table 2 strived to preserve the original detailed information with few adjustments. The number of citations refers to the number of times a given organ of a plant (eventually subjected to a specific preparation) has been mentioned for the same use. As a result, the same informant may have determined multiple citations for the same plant.

Two additional tables were created to facilitate the extraction of information from Table 2, namely Additional file 1: Tables S2 and S3, which list the taxa cited in each use category and relative subcategories.

### Bibliographic survey

In order to compare the results of this study to the general ethnobotanical knowledge in Italy, it was consulted, in first place, the book by Guarrera [12], which reviews the ethnobotanical uses of the plants in Italy and includes all the use categories here considered. Twenty-two plants resulted not listed by Guarrera, and the focus was restricted on the 13 wild native (considered most relevant for ethnobotany). Hence, a bibliographic survey was carried out on these 13 plants to investigate whether the traditional uses here found were reported also in previous ethnobotanical studies. This survey was performed by Scopus and PubMed research services using as key words: the plant species (either with the actual scientific name or any name used before) AND “ethnobotany,” or “traditional uses,” or to restrict the search, the specific use(s) found in our work.

## Results and discussion

### General picture and comparison with literature

Three hundred and seventy-four taxa (belonging to 92 families, and 276 genera) (Additional file 1: Table S1, Table 2) emerged from the survey, leading to the acquisition and systematization of the ethnobotanical knowledge associated with them. Out of these 374 taxa, 251 are plants wild native, 40 wild alien, 74 cultivated and 6 are natural products purchased by people from the market.

The study, involving a total of 1172 informants, was conducted in 22 municipalities in the district of Bologna (Fig. 1), which were grouped into three areas: hill, mountain, and plain.

The vegetation in Bologna district is influenced by altitude and longitude gradients. The altitude gradient encompasses a sub-Mediterranean zone, a middle European zone, a sub-Atlantic zone, and an Oroboreal zone. The longitudinal gradient is determined by the distance from the Adriatic Sea and becomes more evident from the hill to mountain areas. Regarding the plain area, this longitudinal gradient is difficult to detect, due to the general high level of urbanization in this region, which hinders the presence of continuous natural environments.

In the lowland, the vegetation is characterized by ruderal, disturbance-tolerant, and vegetal species in the cultivated areas; exotic species are numerous and abundant, especially along transport infrastructures, rivers, and drainage canals. Mixed *Quercus* forests (*Q. pubescens* Willd., *Q. petraea* (Matt.) Liebl., *Q. cerris* L.) are the natural vegetation in the hilly and sub-montane areas (up to 800–900 m a.s.l.), whereas the montane belt (1000–1600 m a.s.l.) is nearly entirely dominated by *Fagus sylvatica* L. forests. In the subalpine belt, the plant landscape is characterized by wide *Vaccinium myrtillus* L. and *V. gaultherioides* Bigelow heathlands, here and there mixed with *Juniperus communis* L. [13, 14].

**Fig. 2:**
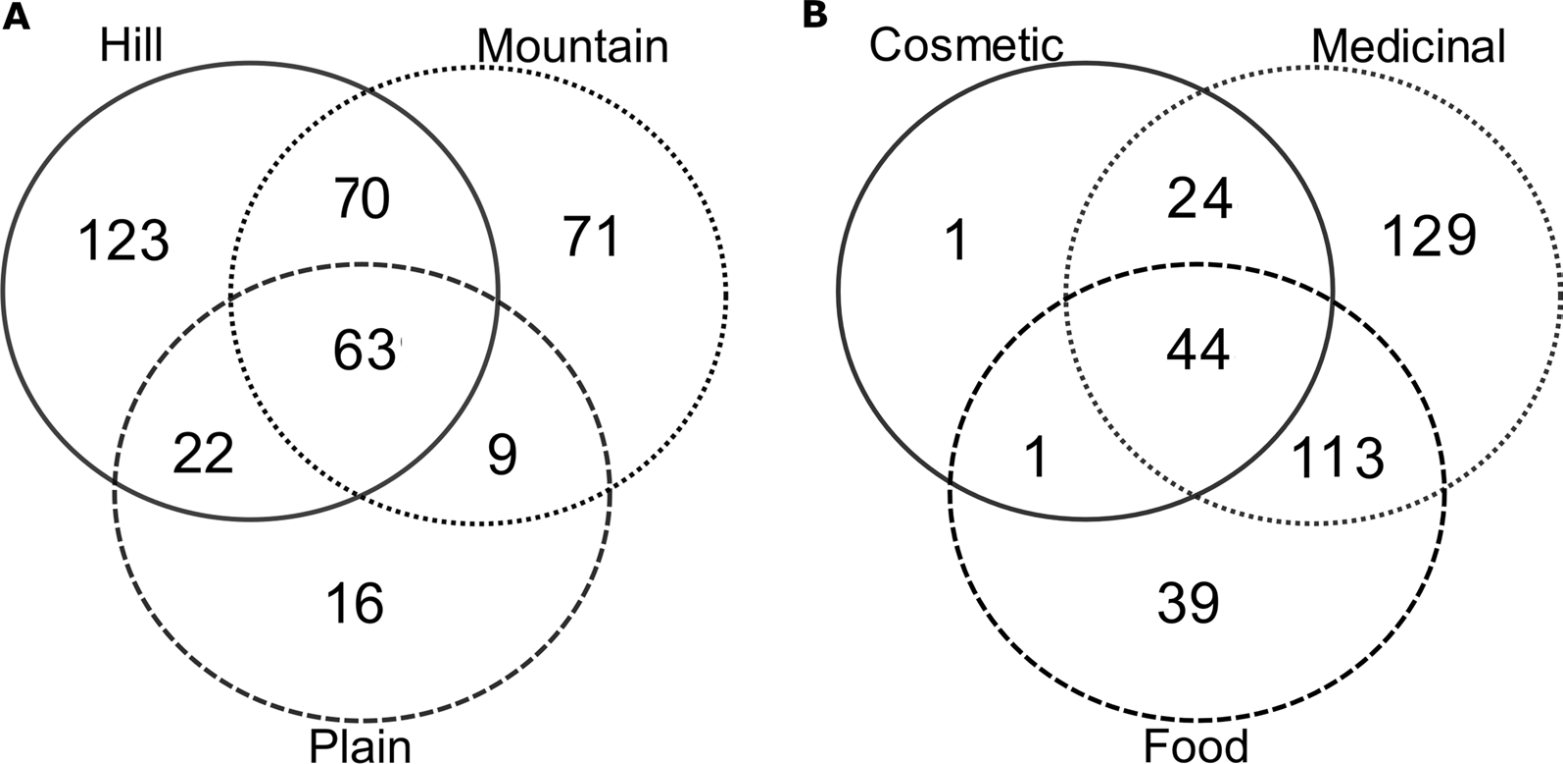
Venn diagrams reporting: **A** the number of taxa emerging from the survey in the different areas; **B** the number of taxa with medical, food, and cosmetic use. The diagrams were obtained using the web tool: http://bioinformatics.psb.ugent.be/webtools/Venn/ [15]

The three areas: hill, mountain, and plain, gave information on 278, 213, and 110 taxa, respectively. Noteworthy, only 63 of them were in common between all three areas (Fig. 2A). It does not surprise that in the plain, which is highly urbanized and with reduced local flora, were identified only 16 plants cited exclusively in this area (Fig. 2A).

All the detailed information on the cited uses of the taxa is summarized in Table 2, striving to offer a complete picture of the traditional knowledge held by the local people of the investigated area.

The information was systematized into 12 use categories (UC): medicinal (MED), food (FOOD), cosmetic (COSM), domestic (DOM), superstitious–magical–religious (SMR), agropastoral (AGROPA), craft (CRAFT), repellent-insecticide (REP), veterinary (VET), toxic (TOXIC), games (GAME), other uses and information (OUI).

**Fig. 3:**
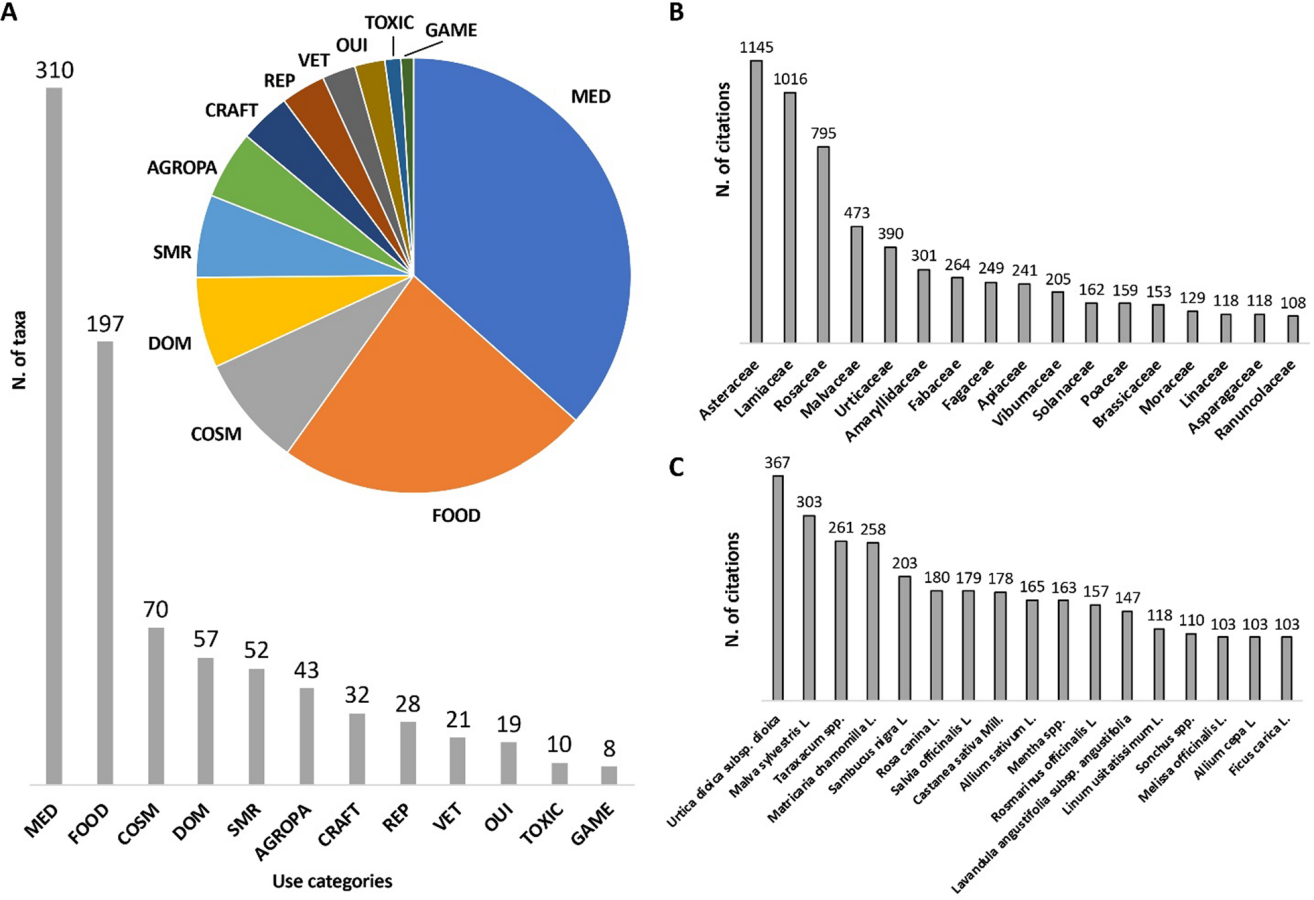
**A** Histogram: number of taxa cited for a specific use category (UC). Pie chart: Percentage of UC, calculated on the basis of the total number of taxa in each UC. **B** Most cited (more than 100 total citations) families **C** Most cited taxa, considering all the UC

The most relevant use of the plants was in traditional medicine (Fig. 3A). In fact, MED was the most important UC both in terms of number of taxa (310) and number of citations, accounting for 4446 citations in total. In terms of importance, MED was immediately followed by FOOD. A picture of the number of taxa in relation of the three most relevant UC (MED, FOOD, and COSM) is given in the Venn diagram of Fig. 2B.

Seventeen families (Fig. 3B) and 17 taxa (Fig. 3C) received more than 100 citations (considering all UC). Asteraceae, Lamiaceae, and Rosaceae were the three most cited families, and *Urtica dioica* (367 cit.), *Malva sylvestris* (303 cit.) and *Taraxacum* spp. (261 cit.) were the three most cited taxa. In terms of exploitation in the UC, the most versatile plants were: *Lavandula angustifolia* (cited in 9 UC), *Urtica dioica* (8 UC), *Juniperus communis* (7 UC), *Rosa canina* (7 UC), *Castanea sativa* (7 UC), *Juglans regia* (7 UC).

The results of this study point out that the emerging taxa were very versatile not only in terms of UC but also for the high diversification in the preparation and organs used for each taxon. In fact, considering all the UC, the majority of the taxa (270; 72% of the total taxa) had more than one preparation, and, similarly, 230 taxa (61.3% of the total) had more than one organ of interest.

**Fig. 4:**
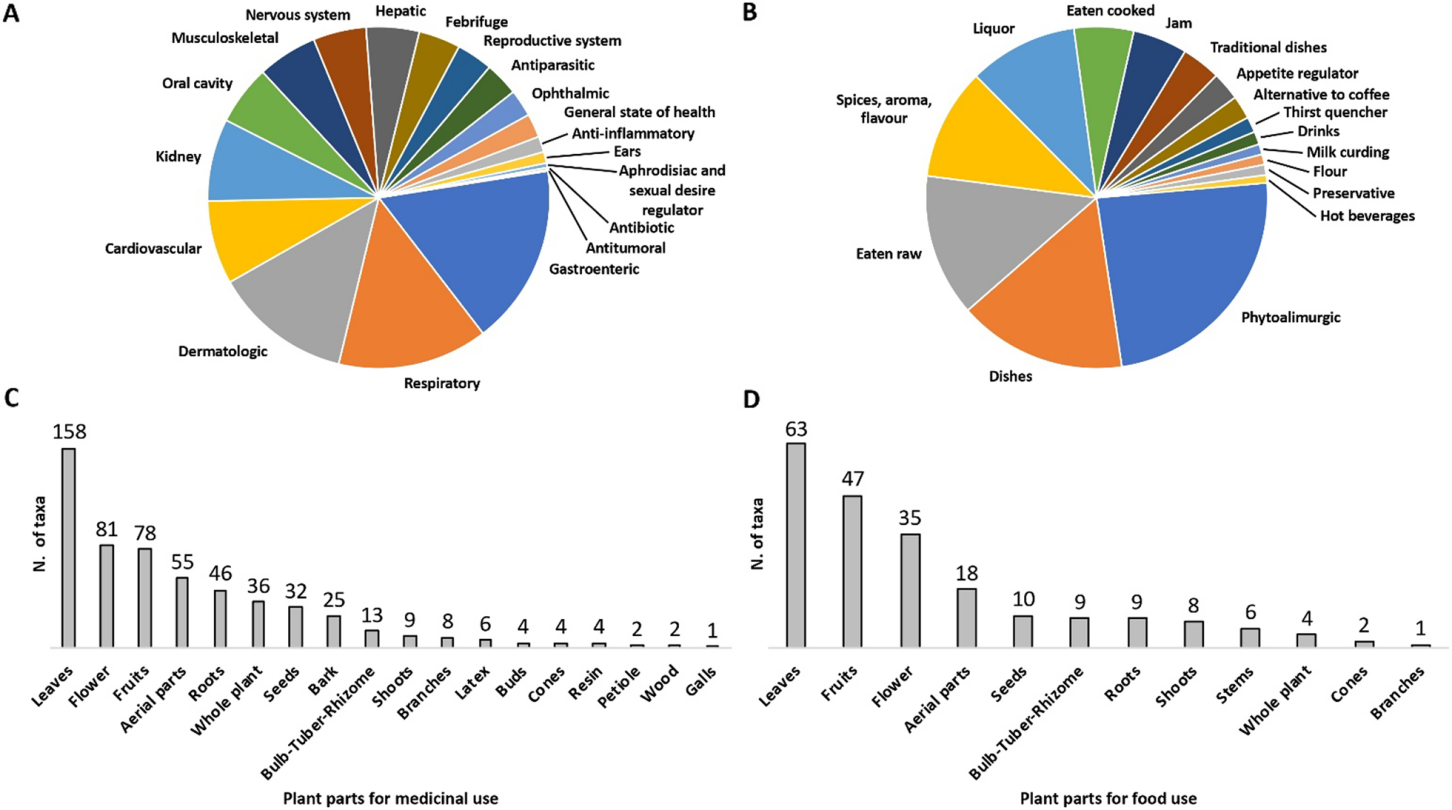
**A** MED subcategories for number of cited taxa, **B** FOOD subcategories for number of cited taxa, **C** plant parts used in MED, **D** plant parts used in FOOD

The most frequent preparations were infusion and decoction administrated *per os* or for external use. Focusing only on MED and FOOD, the most used organ was the leaf (leaf of 158 taxa were in MED and 63 in FOOD), followed by the flower (81 taxa MED and 35 FOOD), and the fruit (78 taxa MED and 47 FOOD) (Fig. 4C, D).

To obtain a first comparison of the obtained results with the general ethnobotanical knowledge of Italy, the book by Guarrera [12] was consulted. As a result, 22 plants cited in this survey were not listed there. Hence, out of these 22 plants, the focus was restricted to the 13 wild native (considered most relevant for ethnobotany), and a bibliographic survey was carried out in order to investigate whether the traditional uses of these plants here found were also reported in other ethnobotanical studies.

According to this search, no previous ethnobotanical studies reported in scientific literature were giving information on the use of two plants, namely: *Globularia bisnagarica* (UC in our study = MED) and *Soldanella alpina* (UC = MED), both plants were cited laxative, and *G. bisnagarica* also as diuretic. The other 11 wild native plants emerging from our study were found also in previous ethnobotanical works developed in countries other than Italy, with several similarity in the uses. These 11 plants were: *Alkekengi officinarum* (UC in our study = MED, FOOD, TOXIC) [16–19], *Dactylis glomerata* (UC = FOOD*,* AGROPA) [20, 21], *Elymus repens* (UC = FOOD*,* MED, AGROPA) [18, 22–24], *Erigeron canadensis* (UC = FOOD) [25], *Galium sylvaticum* (UC = MED, FOOD) [26], *Genista tinctoria* (UC = CRAFT) [18, 27], *Meum athamanticum* (UC = FOOD) [28–30], *Persicaria hydropiper* (UC = FOOD)[31], *Picea abies* (UC = MED) [32, 33], *Ranunculus ficaria* (UC = MED) [18, 34], and *Tanacetum corymbosum* (UC = MED, DOM) [18, 35]. However, revising all the literature found, it resulted that specific traditional uses of 5 of these plants were reported for the first time in our work. In particular, it was never reported before the uses of *Erigeron canadensis* as rennet, of *Ranunculus ficaria* leaves as a remedy for corns, of *Galium sylvaticum* for milk curding and as anti-inflammatory, the use to craft ropes of *Genista tinctoria*, and the nutraceutical use of *Meum athamanticum* leaves and stems, eaten for their deflating and digestive properties.

### Medicinal use (MED)

Three hundred and ten taxa were cited in MED (82.9% of total taxa), representing the 37% of taxa per UC (Fig. 3A). The three most cited species for MED were: *Malva sylvestris* (280 cit.), *Matricaria chamomilla* (223 cit.), and *Linum usitatissimum* (114 cit.).

As shown in Fig. 4A, when considering the taxa cited in the MED subcategories, the highest number (160 taxa; 17% of the total) was found for the subcategory Gastroenteric (including treatment of generic gastrointestinal issues, digestive problems, laxative, astringent, abdominal pain, colitis, ulcer, stomachache, nausea, vomit, aerophagy), followed by Respiratory (cold, cough, catarrh, bronchitis, flu, expectorant, throat issues, asthma) (133 taxa; 14% of the total), and Dermatologic (burns, wounds, acne, blisters, eczema, psoriasis, pimples, insect bites, redness, herpes, warts, anti-sweat) (122 taxa; 13% of the total). Similarly, based on the number of citations, the most important MED subcategories were again Gastroenteric (863 citations, 19% of the total citations in MED), followed by Respiratory (786 cit., 18% of total MED), and Dermatologic (716 cit., 16% of total MED). The remaining 16 subcategories, gathering less than 10% of total taxa and citations, were (in alphabetic order): Antibiotic, Antiparasitic, Antitumoral, Aphrodisiac and Sexual Desire Regulator, Cardiovascular (varicose veins, hemorrhoids, blood pressure, legs edema, nosebleed, chilblains, headache), Ears (otalgia), Febrifuge, General State of Health (preventive, invigorating), Hepatic (purifying, depurative) Anti-inflammatory, Kidney (diuretic, cystitis, kidney stones), Musculoskeletal (arthritis, rheumatics, arthrosis, bone pain, wryneck), Nervous System (depression, insomnia, sedative, memory loss, focus, dizziness, migraine) Ophthalmic (stye, eye redness, and swollen), Oral Cavity (canker sores, halitosis, gingivitis, gums, toothache, abscesses), Reproductive System (menopause, menstrual pains, menorrhagia).

The majority of the taxa (218 taxa, 70.3% of the total MED) had more than one MED subcategory, and the top 7 species with more than one MED subcategory (≥ 10 subcategories) were: *Salvia officinalis* (12 MED subcategories), *Malva sylvestris* (12 MED subcategories), *Rosmarinus officinalis* (11 MED subcategories), *Matricaria chamomilla* (11 MED subcategories), *Sambucus nigra* (10 MED subcategories), *Petroselinum crispum* (10 MED subcategories), *Allium cepa* (10 MED subcategories). The most cited taxa for each MED subcategory are reported in Additional file 1: Table S4.

### Food use (FOOD)

One hundred and ninety-seven taxa (52.7% of the total taxa, representing the 23% of the taxa per UC (Fig. 3A)) were cited for FOOD, and out of these, 28 taxa had only food use. The most cited taxa for this UC were *Urtica dioica* (185 cit.), *Taraxacum* spp. (153 cit.), *Sonchus* spp. (107 cit.).

FOOD was further divided in 16 subcategories (Fig. 4B). Five of these subcategories included plants used in general cookery: (1) Eaten row or in salad, (2) Eaten cooked (generally in soup, boiled or fried), (3) Dishes (used to prepare general dishes sweets and desserts), 4) Traditional dishes, (5) Spices, aroma, and flavor. Seven other FOOD subcategories were related to specific food preparations, namely plants used for: (6) Milk Curding, 7) Flour, (8) Jam, (9) Liqueur (and other alcoholic beverages), (10) Drinks, (11) Hot Beverages, (12) Coffee-substituting Beverages. Another important FOOD subcategory included plants eaten for their (13) Nutraceutical properties. Finally, the uses as (14) Food Preservatives, (15) Thirst Quenchers, and (16) Appetite Regulators were also reported. To facilitate access to the general FOOD information, Additional file 1: Table S2 reports all the taxa cited for each FOOD subcategory.

Ninety-eight taxa (the highest number of taxa cited for a FOOD subcategory) were listed in the Nutraceutical subcategory. In fact, according to the informants they had diverse and numerous beneficial properties associated with their use as food. This result underlines the importance that the Mediterranean tradition has always given to what nowadays has developed into the nutraceutical approach. In general, the survey revealed that several plants are used in cookery for their detoxifying, purifying, digestive, deflating, astringent, laxative, energizing, remineralizing, and diuretic properties. Moreover, some plants were eaten for more specific beneficial effects. For instance, chili, pomegranate, or onion were recommended to improve blood circulation, strawberry to lower blood pressure, *Sonchus* spp. leaves for their beneficial effect on the heart, and sea barely to prevent heart conditions. Biscuits done with carob leaves are eaten to relieve stomach acidity, lettuce soup for stomachaches, wall barely (*Hordeum murinum*) for gastritis, and fennel to reduce vomiting associated with pregnancy. *Celtis australis* fruits were claimed to reduce stress and depression, walnuts to decrease stress and relieve migraines, wall barely to aid in focusing, and onions to induce sleep. Cooked rosehip shoots and dogwood fruits are eaten to relieve sore throats, strawberries to treat flu, wall barely to prevent lung conditions, and onions are eaten in salads as they have disinfecting properties for the throat and oral cavity. Several fruits, such as strawberry, medlar, cherry, and rosehip, are eaten for their anti-inflammatory properties, and *Hippophae rhamnoides* fruits are consumed to strengthen the immune system. Leek soup is used to cure arthritis and gout. Spinach leaves are believed to provide strength to muscles and bones. *Cynara cardunculus* (both flower and leaves) is considered a food with protective and curative properties on the liver. Fresh raspberries eaten in large quantities are believed to help fetal development, nettle is recommended during breastfeeding, and walnuts are claimed to help in staying young.

Forty-three species were used as spices, aroma, and flavor, including also plants chewed for their pleasant taste or to refresh the mouth, such as mint, *Lamium amplexicaule* (for its mint-like taste), *Rumex acetosa* (for the sour taste), *Polypodium vulgare* (for the licorice-like taste), and flowers sucked for their sweet taste, such as *Lonicera periclymenum*, *Anacamptis morio*, and *Primula vulgaris.*

Interestingly, 9 taxa were used to prepare hot beverages as a substitution for coffee during times of war and famine. A specific organ of these plants was roasted and drunk for the coffee-like color and bitter taste of its decoction, and they are: root of *Cichorium intybus*, fruits of *Fagus sylvatica*, fruits *Hordeum vulgare*, fruits of *Hordeum murinum*, acorns of *Quercus pubescens,* acorns of *Quercus robur*, seeds of *Ruscus aculeatus*, roots of *Sonchus* spp., and roots of *Taraxacum* spp.

Half of the taxa had more than one FOOD subcategory (102 taxa, 52% of FOOD taxa), and 37 taxa (19% of FOOD taxa) had more than one organ of interest, showcasing the extensive traditional knowledge about plants in cookery in the province of Bologna. The top five plants with more than one FOOD subcategories (≥ of 6 subcategories) are: *Sambucus nigra* (7 subcategories), *Mentha* spp. (6 subcategories), *Achillea millefolium* (6 subcategories), *Rosa canina* (6 subcategories), *Foeniculum vulgare* (6 subcategories).

### Cosmetic use (COSM)

Seventy taxa were listed in the UC Cosmetic (COSM) (18.7% of total taxa, representing the 8% of the taxa per UC in Fig. 3A). The three most frequently cited species in COSM were *Urtica dioica* (48 cit.), *Salvia officinalis* (33 cit.), and *Matricaria chamomilla* (26 cit.). Detailed information about the preparation and organs used can be found in Table 2.

**Fig. 5:**
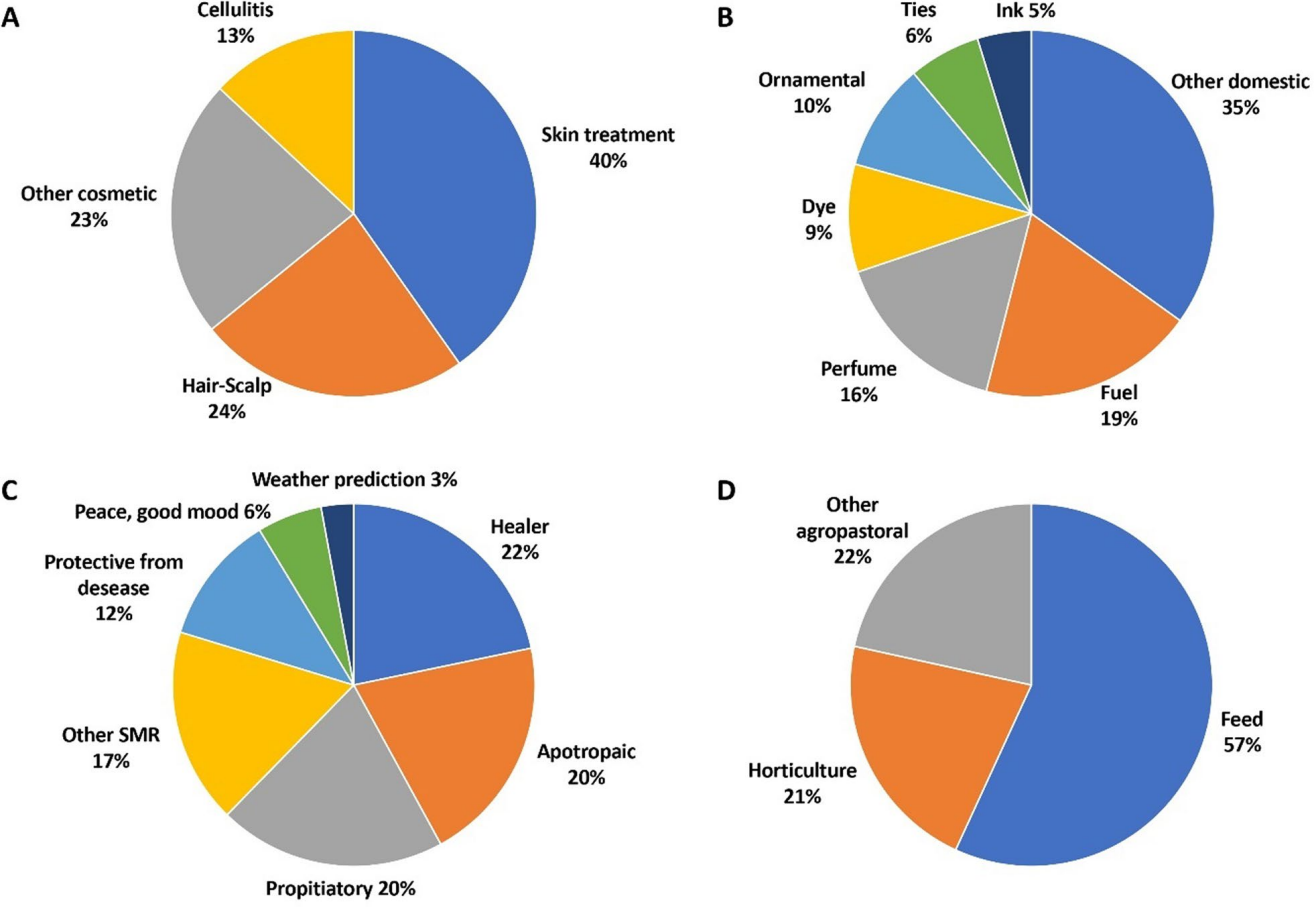
Number of taxa cited for each specific subcategory of: **A** cosmetic (COSM); **B** domestic (DOM); **C** superstitious–magical–religious (SMR); and **D** agropastoral (AGROPA)

The COSM subcategories (Fig. 5A) are as follows: (1) Hair-Scalp, which includes treatments for hair loss, hair and scalp strengthening, shine and hair dyeing, as well as remedies for oily or dry hair and dandruff; (2) Skin treatment, covering cosmetic applications such as emollient, redness reduction, skin refreshment, cleansing, rejuvenating, anti-wrinkles treatments, whitening and suntan agents; (3) Cellulitis treatment, (4) Other cosmetics, which encompasses various cosmetic applications not includible in the previous subcategories, such as counteracting eye bags and swelling, reducing dark circles under the eyes, footbaths for refreshing and preventing excessive sweating, perfumes, teeth-whitening agents, and to obtain a red lipstick. To facilitate access to this information, Additional file 1: Table S2 reports the taxa cited in each COSM subcategory.

*Urtica dioica* had the highest number of citations in COSM (48 cit), while *Juglans regia*, *Lavandula angustifolia*, *Matricaria chamomilla*, *Salvia officinalis*, and *Rosmarinus officinalis* had the highest number of COSM subcategories; they were, in fact, cited in 3 diverse COSM subcategories.

The primary use among the COSM subcategories was Skin treatment, which included 37 taxa, accounting for 40% of the total listed in COSM.

### Domestic (DOM) and Craft (CRAFT) uses

Numerous and diverse domestic uses (DOM) of plants emerged from this work. Fifty-seven taxa were cited for this UC (15.2% of total taxa representing the 7% of the total taxa per UC in Fig. 3A). The three most cited plants were *Lavandula angustifolia* (50 cit.), *Syzygium aromaticum* (10 cit.), *Sambucus nigra* (8 cit.). The subcategories of DOM were as follows (Fig. 5B): (1) Fuel (included plants used to light wood ovens, fireplaces and stoves, and plants used to obtain oil for lamps), (2) Ornamental, (3) Dye for fabric, (4) Perfume (for wardrobe, linen closets, and clothes, as well as for general perfumes to freshen the house and overcome bad smells), (5) Ties, (6) Ink, (7) Other domestic. The latter included abrasive plants used to clean glass, bottles, and flasks, plants used as laundry detergent, to wash wool, as stain-remover for clothes, to shine wood furniture, to cool wine bottles, for wool carding. Curiously, this DOM subcategory included the use of *Arctium minus* as “toilet paper,” *of Triticum aestivum* to make paper, of *Verbascum thapsus* to obtain wicks for oil lamps, and *Hordeum vulgar*e that was treated to obtain a yellow paper, used for wrapping food. All the plants cited in the DOM subcategories are reported in Additional file 1: Table S2.

Thirty-two taxa (8.5% of total taxa representing the 4% of the pie chart shown in Fig. 3A) were reported for their craft uses (CRAFT). The three most cited taxa in this UC were *Quercus* spp. (9 cit.), *Salix alba* (8 cit.), and *Castanea sativa* (7 cit.). CRAFT included five trees cited as their wood was used to make musical instruments, plants used to make pipes, and for crafting various tools, to make furniture, scaffoldings, and various constructions and to obtain fiber for fabrics. All the plants cited in CRAFT are reported in Additional file 1: Table S3.

### Superstitious–magical–religious use (SMR)

Striving to preserve the traditional knowledge in its entirety, the study also inquired and reported that plant uses linked to superstitions, magic, and religions (SMR). This UC included 52 plants (13.9% of total taxa representing the 6% of the total taxa per UC in Fig. 3A), and all the information acquired for the plants is detailed in Table 2. The most cited species were *Aesculus hippocastanum* (26 cit.), *Stachys recta* (24 cit.), and *Allium sativum* (23 cit.). Except for *Acer campestre*, *Sanguinaria canadensis*, and *Trifolium repens*, all the other taxa listed in SMR were cited also in other UC.

The SMR subcategories (Fig. 5C) were: (1) Apotropaic (able to drive away “evil eye” and evil entities or influences), (2) Propitiatory (able to bring good luck and fortune in life, journeys, agriculture, gambling. and economy), (3) Weather prediction (through divination), (4) Bringing peace and good mood (including also the ability to “purify” places), (5) Healer (able to heal a disease in a superstitious or ritualistic way), (6) Protective (from a specific disease), (7) Other SMR. The latter subcategory included plants used to predict a future marriage, find aquifers, to celebrate pagan marriage, to ward off hail, lightning and thunderbolts, to make desires come true, to make someone fall in love, to make a person more handsome, to prolong life, and to promote premonitory dreams. In this latter subcategory were also inserted plants cited for some dreadful uses, namely *Sambucus nigra* and *Linaria vulgaris*. Interestingly, five plants were harvested during the night before the 24th of June (night of Saint John) to be effective: *Hypericum perforatum*, *Juglans regia*, *Lavandula angustifolia*, *Pteridium aquilinum*, and *Stachys recta*. Additional file 1: Table S2 reports all the plants used for each SMR subcategory.

### Agropastoral (AGROPA) and veterinary (VET) uses

In the Agropastoral category (AGROPA) 43 taxa were cited (5% of total taxa representing the 5% of total taxa per UC in Fig. 3A), with the most cited ones being *Quercus* spp. (20 cit.), *Urtica dioica* (17 cit.), *Robinia pseudoacacia* (12 cit.), and *Medicago sativa* (12 cit.). AGROPA included three subcategories (Fig. 5D): (1) Feed, (2) Horticulture (including anti-weed, soil fertilizer and enricher, anti-bacterial and anti-fungal, and weeds), and (3) Other Agropastoral, including plants used to make animal bedding and to attract bees. (Additional file 1: Table S2 lists the plants used in the AGROPA subcategories.)

Some plants of the subcategory Feed were considered not only nutritive but also able to improve the quality of products derived from the animals.

In the veterinary category (VET), 21 taxa were included (5.6% of total taxa representing the 3% of the pie chart shown in Fig. 3A). The most cited taxa were *Helleborus viridis* (37 cit.), *Petroselinum crispum* (4 cit.), and *Fraxinus* spp. (4 cit.).

### Repellent-insecticide use (REP), toxic, games, and other uses and information (OUI)

In the repellent-insecticide category (REP), twenty-eight taxa were listed (7.5% of total taxa representing the 3% of the pie chart shown in Fig. 3A), and the most cited species were *Lavandula angustifolia* (33 cit.), *Urtica dioica* (17 cit.), and *Syzygium aromaticum* (12 cit.).

Some plants were considered repellent for insects or parasites in general, some were specified to have repellent action against mosquitos, aphids, moths, lice, flies, gadflies, and fleas. Some other plants were considered repellent for animals such as scorpions, moles, spiders, vipers and mice or insecticidal, and pesticidal. Ten toxic plants were listed in TOXIC and (for easy access, see Additional file 1: Table S3). Eight taxa were cited by the interviews for their uses in games that were generally played by kids (see *Arctium lappa*, *Primula* spp., *Primula veris,* and *Crategus monogyna*). Nineteen taxa were listed in “Other Uses and Information” (OUI). This UC included uses related to times of shortage and war, for instance, plants used as tobacco-substitutes and other uncommon uses (see Table 2 and the list in Additional file 1: Table S3).

### Phytonyms

The plant names (phytonyms) were given by the interviewees in Italian or, more often, in the dialect of Bologna (Table 2). Some of these dialectal phytonyms were of particular interest and could be divided into six groups: phytonyms derived from plant (1) medicinal use, (2) food use, (3) connection to rituals, myths, or saints, (4) practical uses (5) growth environment or morphological traits, and (6) smell. Examples of the plant of the first group are *Aethusa cynapium* L. named “Erba dla vòs” (herb of the voice) referring to the popular use of the dried leaves in infusion to treat hoarseness, *Angelica sylvestris* L. named “Erba di cavei” (herb of hair), deriving from the tradition of using the boiled flowers to treat baldness, *Centaurium erythraea* Rafn. named “Êrba da la fîvra” (fever herb) and used to lower fever, *Chelidonium majus* L. named “Êrba di pôr” (leek herb) since the caustic juice was applied on warts and leeks, *Delphinium staphisagria* L. named “Êrba pr i bdûc'” (lice herb) widely used as a popular anti-lice, *Echium vulgare* L. named “Erba viperina” (viper grass) since the decoction of the roots was believed to be an antidote against snake venom, *Euphrasia officinalis* L. named “Èrba pr'i och” (herb for the eyes) from its use to treat eye conditions, *Hepatica nobilis* Schreb named “Erba di Bogn” (pimple herb) from the use of the leaf juice to treat pimples, *Hylotelephium maximum* L. Holub named “Erba della Madonna” (Holy Virgin's herb) referring to the excellent healing properties of its leaves which act like “a miracle of the Holy Virgin” for the wounds, *Polygala vulgaris* L. named “Erba da la tass” (cough herb) from the popular use of its decoction against coughs and bronchitis, *Taraxacum* spp. named “Pessalet” (bedwetter) referring to the strong diuretic properties attributed to this plant.

In the second group are found plants such as *Aesculus hippocastanum* L. named “Castagna mata” (mad chestnut), since the seeds look like chestnuts but are not good for eating, *Anacamptis morio* named “Fior ch' as surcen” (flowers that are sucked) from the popular tradition of sucking the flowers for their sweet flavor, *Armoracia rusticana* G. Gaertn., B.Mey. and Scherb named “Cren” (camouflage), for the acrid and spicy root popularly used for its acidic flavor capable of covering the unpleasant tastes that the meat takes on due to poor preservation, *Clematis vitalba* L. called “Asparago dei poveri” (poor man's asparagus) because its young shoots can be consumed like asparagus, hence they were harvested in spring by poor people. *Crepis sancta* L. Babc. named “Ciocapiat” literally hitter-of-pots, a dialect name used to indicate sellers of dishes who boast of their robustness by banging them against each other; this term was also used to indicate a charlatan and here comes the association with the *Crepis sancta* that has a taste similar to chicory but it is a wild plant and a less valuable food. *Galium sylvaticum* L. named “Caglio di bosco” (forest rennet) for it was used by shepherds for milk curdling, *Lathyrus oleraceus* Lam., named “Mangiatutto” (eat-all) so called because every part of these plants is eaten, even the pod, *Polypodium vulgare* L. named “Faelza dulza” (sweet fern) or “Falsa liquirizia” (fake licorice) from the sweetish flavor of the rhizome, similar to that of licorice.

Examples of the plants of the third group were *Linaria vulgaris* subsp. *vulgaris* named “Êrba däl stréjj” (witches' herb), since in popular tradition the plant was used by wizards and witches performing evil spells, *Stachys recta* L. named “Êrba d'la pôra” (herb of fear), since it was used in several rituals to heal traumas and fear, and it was popularly believed that children's fears could be washed away by adding its decoction to bath water. *Barbarea vulgaris* named “Barbarea,” “Barbarella,” or “Erba di Santa Barbara” (St. Barbara herb), so called because the leaves were eaten on December 4, the day on which Santa Barbara is celebrated, or *Hypericum perforatum* L. called “Erba ‘d San Zvàn” (St. Joan Herb) since it is traditionally harvested the night of St. Joan. *Lilium candidum* L. named “Giglio di Sant’Antonio” (lily of St. Anthony), since the pure white lily represents the penitents following St. Anthony in the path toward God through the renunciation of material pleasures to exalt the spiritual ones. *Veronica officinalis* L. called “Occhi della Madonna” (Holy Virgin’s eyes) from the particular light blue color of the little flowers associated with the eyes of the Holy Virgin.

The fourth group is related to phytonyms referring to practical uses, and includes *Cytisus scoparius* subsp. *scoparius* called “Ginestra dei Carbonai” (broom of the charcoal burners) from the popular tradition according to which the charcoal burners used the branches of this plant to build the roofs of the huts where they worked in the summer, *Dipsacus fullonum* L. named “Cardo dei lanaioli” (wool workers' thistle) since the thorny infructescences of the plant were used by weavers to card woolen fabrics, *Helleborus foetidus* L. named “Cavadenti” (teeth-remover) since the rhizome, positioned between the tooth and the gum, was used for the extraction of the teeth. *Ilex aquifolium* L. and *Ruscus aculeatus* L. in addition to other different local names were also both called “Ponztop” (literally biting-mouse) referring to the fact that for their sharp leaves, they were placed around the supplies to keep mice away. *Sorbus aucuparia* L. was called “Sorbo degli uccellatori” (rowan’s fowler) for the birds that were nesting on this plant to feed on its red berries and so they were captured. *Parietaria officinalis* L. called “Erba vetriola” (sandpaper-herb) for the frequent use of its leaves to clean glass, demijohns and bottles, thanks to the fuzz that covers the entire plant which makes it almost similar to sandpaper.

The phytonyms of the fifth group were related to specific features of the plant, such as *Cornus sanguinea* L. called “Sanguinella” (bloody) from the red color of the bark of the winter branches. *Dactylis glomerata* L. “Erba mazzolina” (Bouquet grass) for the flowers are gathered in dense and flat spikelet, forming “bunches” separated from each other. *Delphinium consolida* subsp. *Consolida* called “Speronella” (spur-like) due to the spur shape of its light blue flower, *Equisetum arvense* L. called “Erba cavallina” (horse grass) or “Coda cavallina” (horsetail) from the shape of the adult plant which resembles a horse's tail. *Euonymus europaeus* L. named “Berretta del prete” (priest's hat) from the shape of the fruits which recall the segmented cap with central pompom once used by Catholic priests. *Euphorbia cyparissias* L. named “Erba Latarola” (latex-producing herb) from the acrid and poisonous whitish latex produced by the plant, *Helichrysum italicum* (Roth) G. Don named “Perpetuino” (perpetual) referring to the inflorescences which continue to maintain their appearance and color even when withered. *Laburnum anagyroidis* Medik. named “Maggiociondolo” (May-pendant) alluding to the flowers in pendant clusters that bloom in May. *Lonicera caprifolium* L. named “Ligabòsc” (literally who-ties-the woods) since it is a climbing plant, while *Glechoma hederacea* is named “Laͤddra terræstra” (ground ivy) for its climbing habit on the ground. *Ranunculus arvensis* L. named “Piè gallo” (rooster's foot) for its leaves, which resemble the feet and combs of a rooster. *Silene vulgaris* (Moench) Garcke, named “Sciopetin” or “Ciuchaͤtt” (crackling) for its flower that “pops” if held between the fingers. *Tragopogon pratensis* L. named “Barba di becco” (goat’s beard) that seems to come from the Lombardic language “bikk” meaning goat, or called “Barba d’frè” (monk’s beard) since the infructescence (made of achenes with pappus) resembles a bearded face. Other phytonyms in this group are related to plant growth conditions or environment, such as *Asplenium ceterach* L. named “Erba rugine” (rust grass) for the reddish spores arranged on the underside of the leaf, or also called “Spaccapietre” (stonebreaker) because it grows tenaciously on rocks and walls, slowly penetrating the stones, similar name for the same reasons was given also to *Celtis australis* L. named “Spaccasassi” (stonebreaker). *Globularia bisnagarica* L. called “Morina” (young widow) because, between March and May, it blooms in dry meadows and pastures alone, surrounded by no other flowers. *Lamium amplexicaule* L. named “Erba ruota” (wheel grass) because the plant is easily found near paths and roads and therefore in contact with the wheels of cars. *Centaurea cyanus* L. named “Garufanin blô de grén” (blue carnation of the wheat) for it grows in the crop fields, sprinkling them with blue spots. *Medicago sativa* L. is called “Erba Spagna” (Spanish grass) because during the Middle Ages its cultivation in Europe was almost abandoned, so much so that in Italy it remained unknown until 1500, when it was reintroduced with seeds imported from Spain where it had been spread again by the Arabs. The last group of phytonyms come from the plant smell and include plants like *Alliaria petiolata*, named “Aj herb” (garlic herb) from its persistent garlic smell, *Aloysia citradora* called “Erba zidreina” (citrine grass) from the citrus smell given by the leaves when rubbed, *Galium odoratum* Scop. Called “Stellina odorosa” (fragrant little star) from the shape of the flowers resembling a star and the scent they produce when dried, *Helleborus foetidus* L. named “Erba zitona” (gypsy grass) with a derogatory connotation, comparing the bad smell of the plant to that of the gypsies, and its toxicity to the fact that one should be careful with this plant as when meeting a gypsy.

## Conclusions

This study demonstrates the extensive knowledge and use of plants in Bologna district, with some differences in the taxa of interest depending on the geographic area where the interviews were conducted (mountain, hill, and plain). The conspicuous number of informants interviewed has led to a wide spectrum of information about a high number of taxa. The majority of the taxa had multiple use categories, multiple organs of interest, and different methods of preparation and administration.

As expected, medicinal use was the most frequently cited category, followed by food uses. However, this study also revealed numerous other fascinating uses of plants, including rituals and superstitious beliefs.

The comprehensive insights gathered through this research are important for the appreciation and preservation of the knowledge and cultural heritage of the local communities. Additionally, this study has the potential to inspire further research in various domains of plant science, uncovering alternative possibilities for the sustainable utilization of plant resources.

Overall, this work not only contributes to the understanding of traditional plant knowledge but also highlights the significance of conserving and transmitting these age-old practices and beliefs for future generations.

### Supplementary Information

**Additional file 1**. **Table S1**: List of the 374 taxa emerging from the study with the family and the area where the taxa were cited (H= hill, M= mountain, P= plain). **Table S2**: List of taxa for the categories Medicinal, Food, Superstitious–Magical–Religious (SMR), Cosmetic (COSM), Agropastoral (AGROPA), Domestic (DOM) divided into their subcategories. **Table S3**: List of taxa in the categories Craft, Toxic, Repellent and Insecticide (REP), Veterinary (VET), Games, Other uses and Information (OUI). **Table S4**: Most cited taxa for MED subcategories. For each subcategory, the total citations number in the overall MED category is reported in brackets and the three most cited taxa are listed giving the number of citations for the specific MED subcategory.

## Data Availability

Data collected in excel will be made available on request.

## Abbreviations

AGROPA: Agropastoral use
Cit: Citation number
COSM: Cosmetic use
CRAFT: Craft use
DOM: Domestic use
FOOD: Food use
GAME: Games
H: Hill area
M: Mountain area
MED: Medicinal use
OUI: Other uses and information
P: Plain area
REP: Repellent-insecticide use
SMR: Superstitious–magical–religious use
TOXIC: Toxic use
UC: Use category
VET: Veterinary use
