# Supplementary material for: Saving the local tradition: ethnobotanical survey on the use of plants in Bologna district (Italy)
Source: J Ethnobiol Ethnomed. 2024 Mar 12;20:33. doi: 10.1186/s13002-024-00664-1 (PMC10936038; doi:10.1186/s13002-024-00664-1)
Supplement: Supplementary file 1 — Additional file 1. Table S1: List of the 374 taxa emerging from the study with the family and the area where the taxa were cited (H= hill, M= mountain, P= plain). Table S2: List of taxa for the categories Medicinal, Food, Superstitious–Magical–Religious (SMR), Cosmetic (COSM), Agropastoral (AGROPA), Domestic (DOM) divided into their subcategories. Table S3: List of taxa in the categories Craft, Toxic, Repellent and Insecticide (REP), Veterinary (VET), Games, Other uses and Information (OUI). Table S4: Most cited taxa for MED subcategories. For each subcategory, the total citations number in the overall MED category is reported in brackets and the three most cited taxa are listed giving the number of citations for the specific MED subcategory. [file 13002_2024_664_MOESM1_ESM.docx]

# **Saving the local tradition: ethnobotanical survey on the use of plants in Bologna district (Italy)**

**Ilaria Chiocchio^a§^, Lorenzo Marincich^b§^, Manuela Mandrone****^a*^, Simona Trincia^a^, Clarissa Tarozzi ^a^, Ferruccio Poli^a^.**

^a^Department of Pharmacy and Biotechnology, Alma Mater Studiorum, University of Bologna, Via Irnerio 42, 40126 Bologna, Italy

^b^Department for Life Quality Studies, Alma Mater Studiorum, Rimini Campus, University of Bologna, Corso d’Augusto 237, 47921 Rimini, Italy

^§^ Equal first authors

^*^ **Corresponding author:** Manuela Mandrone PhD,

Department of Pharmacy and Biotechnology (FaBit), Via Irnerio 42, 40126 Bologna (Italy)

University of Bologna

+390512091294 (office)

email: manuela.mandrone2@unibo.it

**Table S1.** List of the 374 taxa emerging from the study with the family and the area where the taxa were cited (H= hill, M= mountain, P= plain).

| **Taxa** | **Family** | **Area(s)** |
| --- | --- | --- |
| *Abies alba* Mill. | Pinaceae | HM |
| *Acer campestre* L. | Sapindaceae | P |
| *Achillea millefolium* L. | Asteraceae | HMP |
| *Acorus calamus* L. | Acoraceae | H |
| *Adiantum capillus-veneris* L. | Pteridaceae | M |
| *Aesculus hippocastanum* L. | Sapindaceae | HMP |
| *Aethusa cynapium* L. | Apiaceae | M |
| *Agrimonia eupatoria* L. | Rosaceae | HM |
| *Ailanthus altissima* (Mill.) Swingle | Simaroubaceae | H |
| *Ajuga chamaepitys* (L.) Schreb. | Lamiaceae | H |
| *Ajuga reptans* L. | Lamiaceae | H |
| *Alcea rosea* L. | Malvaceae | H |
| *Alkekengi officinarum* Moench | Solanaceae | HM |
| *Alliaria petiolata (*M. Bieb.) Cavara & Grande | Brassicaceae | M |
| *Allium ampeloprasum* L. | Amaryllidaceae | HP |
| *Allium cepa* L. | Amaryllidaceae | HMP |
| *Allium sativum* L. | Amaryllidaceae | HMP |
| *Allium schoenoprasum* L. | Amaryllidaceae | HMP |
| *Alnus glutinosa* (L.) Gaertn. | Betulaceae | P |
| *Aloe vera* (L.) Burm.f. | Asphodelaceae | H |
| *Aloysia citrodora* Paláu | Verbenaceae | HP |
| *Althaea officinalis* L. | Malvaceae | HP |
| *Anacamptis morio* (L.) R.M.Bateman, Pridgeon & M.W.Chase | Orchidaceae | M |
| *Anethum graveolens* L. | Apiaceae | H |
| *Angelica archangelica* L. | Apiaceae | H |
| *Angelica sylvestris* L. | Apiaceae | HM |
| *Anthyllis vulneraria* L. | Fabaceae | M |
| *Antirrhinum majus* L. | Plantaginaceae | M |
| *Apium graveolens* L. | Apiaceae | H |
| *Aquilegia* spp. | Ranunculaceae | M |
| *Arbutus unedo* L. | Ericaceae | MP |
| *Arctium lappa* L. | Asteraceae | HMP |
| *Arctium minus* (Hill.) Bernh. | Asteraceae | H |
| *Arctostaphylos uva-ursi* (L.) Spreng. | Ericaceae | H |
| *Armoracia rusticana* G. Gaertn., B.Mey. & Scherb. | Brassicaceae | P |
| *Artemisia absinthium* L. | Asteraceae | P |
| *Artemisia dracunculus* L. | Asteraceae | H |
| *Artemisia vulgaris* L. | Asteraceae | M |
| *Arundo donax* L. | Poaceae | P |
| *Asparagus acutifolius* L. | Asparagaceae | HP |
| *Asplenium ceterach* L. | Aspleniaceae | HM |
| *Asplenium viride* Huds. | Aspleniaceae | M |
| *Atropa belladonna* L. | Solanaceae | HM |
| *Avena sativa* L. | Poaceae | HM |
| *Ballota nigra* L. | Lamiaceae | M |
| *Barbarea vulgaris* W.T.Aiton | Brassicaceae | M |
| *Bellis perennis* L. | Asteraceae | MP |
| *Beta vulgaris* L. | Amaranthaceae | HP |
| *Betula pendula* Roth | Betulaceae | HM |
| *Betula pubescens* Ehrh. | Betulaceae | H |
| *Borago officinalis* L. | Boraginaceae | HMP |
| *Brassica napus* L. | Brassicaceae | H |
| *Brassica nigra* W.D.J.Koch | Brassicaceae | HM |
| *Brassica oleracea* L. | Brassicaceae | HMP |
| *Buxus sempervirens* L. | Buxaceae | HM |
| *Calendula officinalis* L. | Asteraceae | HM |
| *Calluna vulgaris* (L.) Hill | Ericaceae | M |
| *Campanula rapunculus* L. | Campanulaceae | HP |
| *Cannabis sativa* L. | Cannabaceae | P |
| *Capsella bursa-pastoris* Medik. | Brassicaceae | HMP |
| *Capsicum annuum* L. | Solanaceae | H |
| *Carlina acaulis* L. | Asteraceae | M |
| *Carum carvi* L. | Apiaceae | H |
| *Castanea sativa* Mill. | Fagaceae | HMP |
| *Celtis australis* L. | Cannabaceae | HM |
| *Centaurea calcitrapa* L. | Asteraceae | M |
| *Centaurea cyanus* L. | Asteraceae | M |
| *Centaurea* spp. | Asteraceae | M |
| *Centaurium erythraea* Rafn | Gentianaceae | H |
| *Ceratonia siliqua* L. | Fabaceae | H |
| *Chamaemelum nobile* L. | Asteraceae | M |
| *Chelidonium majus* L. | Papaveraceae | HMP |
| *Chenopodium bonus-henricus* L. | Amaranthaceae | H |
| *Cichorium endivia* L. | Asteraceae | H |
| *Cichorium intybus* L. | Asteraceae | HMP |
| *Cirsium vulgare* (Savi) Ten. | Asteraceae | H |
| *Cistus salviifolius* L. | Cistaceae | H |
| *Citrus* *aurantium* L. | Rutaceae | H |
| *Citrus limon* (L.) Osbeck | Rutaceae | H |
| *Clematis vitalba* L. | Ranuncolaceae | HMP |
| *Clinopodium nepeta* (L.) Kuntze | Lamiaceae | HM |
| *Coffea arabica* L. | Rubiaceae | H |
| *Colutea arborescens* L. | Fabaceae | M |
| *Convallaria majalis* L. | Asparagaceae | H |
| *Convolvulus arvensis* L. | Convolvulaceae | P |
| *Coriandrum sativum* L. | Apiaceae | H |
| *Cornus mas* L. | Cornaceae | HM |
| *Cornus sanguinea* L. | Cornaceae | H |
| *Corylus avellana* L. | Betulaceae | HM |
| *Crataegus laevigata* (Poir.) DC. | Rosaceae | M |
| *Crataegus monogyna* Jacq. | Rosaceae | H |
| *Crataegus* spp. | Rosaceae | HM |
| *Crepis sancta* (L.) Babc. | Asteraceae | P |
| *Crepis vesicaria* L. | Asteraceae | MP |
| *Crocus sativus* L. | Iridaceae | H |
| *Cucumis melo* L. | Cucurbitaceae | H |
| *Cucumis sativus* L. | Cucurbitaceae | HM |
| *Cucurbita maxima* Duchesne | Cucurbitaceae | H |
| *Cucurbita pepo* L. | Cucurbitaceae | H |
| *Cupressus sempervirens* L. | Cupressaceae | H |
| *Cyclamen hederifolium* Aiton | Primulaceae | H |
| *Cydonia oblonga* Mill. | Rosaceae | H |
| *Cynara cardunculus* L. | Asteraceae | HMP |
| *Cynodon dactylon* (L.) Pers. | Poaceae | HP |
| *Cytisus scoparius* subsp. *scoparius* | Fabaceae | HM |
| *Cytisus* spp. | Fabaceae | M |
| *Dactylis glomerata* L. | Poaceae | H |
| *Daucus carota* L. | Apiaceae | HM |
| *Delphinium consolida* (L.) | Ranuncolaceae | M |
| *Delphinium staphisagria* L. | Ranuncolaceae | H |
| *Diospyros kaki* L.f. | Ebenaceae | H |
| *Diplotaxis tenuifolia* (L.) DC. | Brassicaceae | H |
| *Dipsacus fullonum* L. | Caprifoliaceae | M |
| *Dipsacus laciniatus* L. | Caprifoliaceae | H |
| *Dryopteris filix-mas* (L.) Schott | Dryopteridaceae | M |
| *Echinacea angustifolia* DC. | Asteraceae | H |
| *Echium vulgare* L. | Boraginaceae | M |
| *Elymus repens* subsp. *repens* | Poaceae | HMP |
| *Equisetum arvense* L. | Equisetaceae | HMP |
| *Equisetum telmateia* Ehrh. | Equisetaceae | HM |
| *Erigeron canadensis* L. | Asteraceae | M |
| *Eruca vesicaria* (L.) Cav. | Brassicaceae | HMP |
| *Euonymus europaeus* L. | Celastraceae | H |
| *Eupatorium cannabinum* L. | Asteraceae | M |
| *Euphorbia cyparissias* L. | Euphorbiaceae | HM |
| *Euphorbia helioscopia* subsp. *helioscopia* | Euphorbiaceae | HM |
| *Euphorbia lathyris* L. | Euphorbiaceae | H |
| *Euphorbia* spp. | Euphorbiaceae | H |
| *Euphrasia officinalis* L. | Orobanchaceae | H |
| *Fagus sylvatica* L. | Fagaceae | M |
| *Ficus carica* L. | Moraceae | HMP |
| *Filipendula ulmaria* (L.) Maxim. | Rosaceae | H |
| *Foeniculum vulgare* Mill. | Apiaceae | HMP |
| *Fragaria vesca* L. | Rosaceae | HM |
| *Fragaria viridis* Weston | Rosaceae | HM |
| *Frangula alnus* Mill. | Rhamnaceae | H |
| *Fraxinus excelsior*  subsp. *excelsior* | Oleaceae | HM |
| *Fraxinus ornus* subsp. *ornus* | Oleaceae | H |
| *Fraxinus* spp. | Oleaceae | H |
| *Fumaria officinalis* L. | Papaveraceae | H |
| *Galanthus nivalis* L. | Amaryllidaceae | H |
| *Galega officinalis* L. | Fabaceae | HM |
| *Galium odoratum* Scop. | Rubiaceae | H |
| *Galium sylvaticum* L. | Rubiaceae | M |
| *Galium verum* L. | Rubiaceae | HM |
| *Genista tinctoria* L. | Fabaceae | M |
| *Gentiana lutea* L. | Gentianaceae | M |
| *Gentiana* spp. | Gentianaceae | M |
| *Geranium robertianum* L. | Geraniaceae | M |
| *Ginkgo biloba* L. | Ginkgoaceae | H |
| *Glechoma hederacea* L. | Lamiaceae | HM |
| *Globularia bisnagarica* L. | Plantaginaceae | H |
| *Glycyrrhiza glabra* L. | Fabaceae | H |
| *Hedera helix* L. | Araliaceae | HM |
| *Helianthus tuberosus* L. | Asteraceae | HP |
| *Helichrysum italicum* (Roth) G.Don | Asteraceae | HP |
| *Helichrysum stoechas* (L.) Moench | Asteraceae | M |
| *Helleborus foetidus* L. | Ranuncolaceae | M |
| *Helleborus* spp. | Ranuncolaceae | H |
| *Helleborus viridis* L. | Ranuncolaceae | HM |
| *Hepatica nobilis* Schreb. | Ranuncolaceae | M |
| *Hibiscus* spp. | Malvaceae | H |
| *Hippophae rhamnoides L.* | Elaeagnaceae | M |
| *Hordeum murinum* L. | Poaceae | HM |
| *Hordeum vulgare* L. | Poaceae | HMP |
| *Humulus lupulus* L. | Cannabaceae | HMP |
| *Hydrangea macrophylla* (Thunb.) Ser. | Hydrangeaceae | M |
| *Hylotelephium maximum* (L.) Holub | Crassulaceae | HM |
| *Hyoscyamus niger* L. | Solanaceae | H |
| *Hypericum perforatum* L. | Hypericaceae | HMP |
| *Hyssopus officinalis* L. | Lamiaceae | HM |
| *Ilex aquifolium* L. | Aquifoliaceae | HM |
| *Iris* spp. | Iridaceae | H |
| *Juglans regia* L. | Juglandaceae | HMP |
| *Juniperus communis* L. | Cupressaceae | HMP |
| *Laburnum anagyroidis* Medik | Fabaceae | M |
| *Lactuca sativa* L. | Asteraceae | HP |
| *Lamium amplexicaule* L. | Lamiaceae | M |
| *Larix decidua* (L.) Mill. | Pinaceae | M |
| *Lathyrus oleraceus* Lam. | Fabaceae | H |
| *Laurus nobilis* L. | Lauraceae | HMP |
| *Lavandula angustifolia* subsp. *angustifolia* | Lamiaceae | HMP |
| *Lavandula* spp. | Lamiaceae | MP |
| *Leopoldia comosa* (L.) Parl. | Asparagaceae | HM |
| *Leucanthemum vulgare* subsp. *vulgare* | Asteraceae | P |
| *Levisticum officinale* W.D.J. Koch | Apiaceae | H |
| *Lilium candidum* L. | Liliaceae | H |
| *Linaria vulgaris* subsp. *vulgaris* | Scrophulariaceae | H |
| *Linum usitatissimum* L. | Linaceae | HMP |
| *Lonicera caprifolium* L. | Caprifoliaceae | H |
| *Lonicera periclymenum* L. | Caprifoliaceae | M |
| *Lotus corniculatus* L. | Fabaceae | H |
| *Lupinus albus* subsp. *albus* | Fabaceae | H |
| *Malus domestica* (Suckow) Borkh. | Rosaceae | HP |
| *Malva sylvestris* L. | Malvaceae | HMP |
| *Marrubium vulgare* L. | Lamiaceae | HM |
| *Matricaria chamomilla* L. | Asteraceae | HMP |
| *Medicago sativa* L. | Fabaceae | HP |
| *Melilotus officinalis* Pall. | Fabaceae | M |
| *Melissa officinalis* L. | Lamiaceae | HMP |
| *Mentha* spp. | Lamiaceae | HMP |
| *Mercurialis annua* L. | Euphorbiaceae | H |
| *Mespilus germanica* L. | Rosaceae | HM |
| *Meum athamanticum* Jacq. | Apiaceae | M |
| *Morus alba* L. | Moraceae | HP |
| *Morus nigra* L. | Moraceae | H |
| *Morus* spp. | Moraceae | HM |
| *Musa paradisiaca* L. | Musaceae | H |
| *Myosotis arvensis* Hill | Boraginaceae | M |
| *Narcissus jonquilla* L. | Amaryllidaceae | M |
| *Nasturtium officinale* R.Br. | Brassicaceae | HM |
| *Nerium oleander* L. | Apocynaceae | HP |
| *Nicotiana tabacum* L. | Solanaceae | H |
| *Ocimum basilicum* L. | Lamiaceae | HM |
| *Oenothera biennis* L. | Onagraceae | H |
| *Olea europaea* L. | Oleaceae | HP |
| *Onobrychis viciifolia* Scop. | Fabaceae | H |
| *Ononis spinosa* L. | Fabaceae | M |
| *Origanum majorana* L. | Lamiaceae | HM |
| *Origanum vulgare* L. | Lamiaceae | HM |
| *Oryza sativa* L. | Poaceae | H |
| *Osmunda regalis* L. | Osmundaceae | H |
| *Ostrya carpinifolia* Scop. | Betulaceae | M |
| *Oxalis acetosella* L. | Oxalidaceae | HM |
| *Paliurus spina-christi* Mill. | Rhamnaceae | H |
| *Papaver rhoeas* L. | Papaveraceae | HMP |
| *Parietaria officinalis* L. | Urticaceae | HMP |
| *Passiflora caerulea* L. | Passifloraceae | H |
| *Pelargonium* spp. | Geraniaceae | HM |
| *Persicaria hydropiper* (L.) Delarbre | Polygonaceae | M |
| *Petasites hybridus* (L.) G.Gaertn., B.Mey. & Scherb. | Asteraceae | M |
| *Petrosedum rupestre* (L.) P.V.Heath | Crassulaceae | M |
| *Petroselinum crispum* (Mill.) Fuss | Apiaceae | HMP |
| *Phaseolus vulgaris* L. | Fabaceae | HM |
| *Phedimus spurius* (M.Bieb.) 't Hart | Crassulaceae | M |
| *Phytolacca americana* L. | Phytolaccaceae | P |
| *Picea abies* (L.) H.Karst. | Pinaceae | HM |
| *Pilosella officinarum* Vaill. | Asteraceae | H |
| *Pimpinella anisum* L. | Apiaceae | H |
| *Pinus cembra* L. | Pinaceae | H |
| *Pinus mugo* Turra | Pinaceae | HM |
| *Pinus pinea* L. | Pinaceae | H |
| *Pinus* spp. | Pinaceae | H |
| *Pinus sylvestris* L. | Pinaceae | HMP |
| *Plantago afra* L. | Plantaginaceae | H |
| *Plantago lanceolata* L. | Plantaginaceae | HMP |
| *Plantago major* L. | Plantaginaceae | H |
| *Plantago* spp. | Plantaginaceae | HM |
| *Polygala vulgaris* L. | Polygalaceae | M |
| *Polygonum aviculare* L. | Polygonaceae | HM |
| *Polypodium vulgare* L. | Polypodiaceae | HM |
| *Populus alba* L. | Salicaceae | H |
| *Populus nigra* L. | Salicaceae | HP |
| *Portulaca oleracea* L. | Portulacaceae | HP |
| *Primula ciliata* Moretti | Primulaceae | M |
| *Primula* spp. | Primulaceae | M |
| *Primula veris* L. | Primulaceae | H |
| *Primula vulgaris* Huds. | Primulaceae | MP |
| *Prunus armeniaca* L. | Rosaceae | H |
| *Prunus avium* L. | Rosaceae | HMP |
| *Prunus cerasifera* Ehrh. | Rosaceae | H |
| *Prunus cerasus* L. | Rosaceae | HM |
| *Prunus domestica* L. | Rosaceae | HM |
| *Prunus dulcis* D.A.Webb | Rosaceae | H |
| *Prunus laurocerasus* L. | Rosaceae | HM |
| *Prunus persica* (L.) Batsch | Rosaceae | HP |
| *Prunus spinosa* L. | Rosaceae | HM |
| *Pteridium aquilinum* (L.) Kuhn | Boraginaceae | HM |
| *Pulmonaria officinalis* L. | Boraginaceae | HMP |
| *Punica granatum* L. | Lythraceae | H |
| *Pyrus communis* L. | Rosaceae | HMP |
| *Quercus petraea* subsp. *petrea* | Fagaceae | H |
| *Quercus pubescens* subsp. *pubescens* | Fagaceae | M |
| *Quercus robur* L. | Fagaceae | MP |
| *Quercus* spp. | Fagaceae | HM |
| *Ranunculus arvensis* L. | Ranuncolaceae | M |
| *Ranunculus ficaria* L. | Ranuncolaceae | M |
| *Raphanus raphanistrum* subsp. *sativus* (L.) Domin | Brassicaceae | H |
| *Raphanus raphanistrum* L. | Polygonaceae | H |
| *Ribes nigrum* L. | Grossulariaceae | H |
| *Robinia pseudoacacia* L. | Fabaceae | HMP |
| *Rosa canina* L. | Rosaceae | HMP |
| *Rosa gallica* L. | Rosaceae | H |
| *Rosa* spp. | Rosaceae | HP |
| *Rosmarinus officinalis* L. | Lamiaceae | HMP |
| *Rubus idaeus* L. | Rosaceae | HM |
| *Rubus plicatus* Weihe & Nees | Rosaceae | M |
| *Rubus ulmifolius* Schott | Rosaceae | HMP |
| *Rumex acetosa* L. | Polygonaceae | HM |
| *Rumex alpinus* L. | Polygonaceae | M |
| *Rumex crispus* L. | Polygonaceae | MP |
| *Ruscus aculeatus* L. | Asparagaceae | HMP |
| *Ruta graveolens* L. | Rutaceae | HMP |
| *Salix alba* L. | Salicaceae | HMP |
| *Salix caprea* L. | Salicaceae | M |
| *Salix purpurea* L. | Salicaceae | M |
| *Salix* spp. | Salicaceae | H |
| *Salvia officinalis* L. | Lamiaceae | HMP |
| *Salvia pratensis* L. | Lamiaceae | H |
| *Salvia sclarea* L. | Lamiaceae | M |
| *Sambucus ebulus* L. | Viburnaceae | H |
| *Sambucus nigra* L. | Viburnaceae | HMP |
| *Sanguinaria canadensis* L. | Papaveraceae | P |
| *Sanguisorba minor* Scop. | Rosaceae | HP |
| *Santolina chamaecyparissus* L. | Asteraceae | H |
| *Saponaria officinalis* L. | Caryophyllaceae | M |
| *Satureja* spp. | Lamiaceae | H |
| *Saxifraga* spp. | Saxifragaceae | M |
| *Sedum acre* L. | Crassulaceae | H |
| *Sempervivum montanum* L. | Crassulaceae | M |
| *Sempervivum tectorum* L*.* | Crassulaceae | H |
| *Silene vulgaris* (Moench) Garcke | Caryophyllaceae | HMP |
| *Silybum marianum* (L.) Gaertn. | Asteraceae | HMP |
| *Sinapis alba* L. | Brassicaceae | MP |
| *Sinapis arvensis* L. | Brassicaceae | M |
| *Sisymbrium officinale* (L.) Scop. | Brassicaceae | HM |
| *Solanum dulcamara* L. | Solanaceae | HM |
| *Solanum lycopersicum* L. | Solanaceae | H |
| *Solanum melongena* L. | Solanaceae | H |
| *Solanum tuberosum* L. | Solanaceae | HMP |
| *Soldanella alpina* L. | Primulaceae | M |
| *Sonchus* spp. | Asteraceae | HMP |
| *Sorbus aucuparia* L. | Rosaceae | H |
| *Sorbus domestica* L. | Rosaceae | HMP |
| *Sorbus torminalis* (L.) Crantz | Rosaceae | M |
| *Sorghum bicolor* (L.) Moench | Poaceae | P |
| *Spartium junceum* L. | Fabaceae | H |
| *Spinacia oleracea* L. | Amaranthaceae | P |
| *Stachys officinalis* (L.) Trevis | Lamiaceae | H |
| *Stachys recta* L. | Lamiaceae | HM |
| *Stevia rebaudiana* Bertoni | Asteraceae | H |
| *Sulla coronaria* (L.) | Fabaceae | H |
| *Symphytum officinale* L. | Boraginaceae | H |
| *Syringa vulgaris* L. | Oleaceae | H |
| *Syzygium aromaticum* (L.) Merr. & L.M.Perry | Myrtaceae | HM |
| *Tanacetum balsamita* L. | Asteraceae | HM |
| *Tanacetum corymbosum* (L.) Sch.Bip. | Asteraceae | H |
| *Tanacetum parthenium* (L.) Sch.Bip. | Asteraceae | H |
| *Taraxacum* spp. | Asteraceae | HMP |
| *Taxus baccata* L. | Taxaceae | H |
| *Teucrium chamaedrys* L. | Lamiaceae | HM |
| *Thymus vulgaris* subsp. *vulgaris* | Lamiaceae | HMP |
| *Tilia cordata* Mill. | Malvaceae | M |
| *Tilia platyphyllos* Scop. | Malvaceae | HMP |
| *Tilia* spp. | Malvaceae | H |
| *Tragopogon pratensis* L. | Asteraceae | P |
| *Trifolium pratense* L. | Fabaceae | H |
| *Trifolium repens* L. | Fabaceae | H |
| *Trigonella foenum-graecum* L. | Fabaceae | H |
| *Triticum aestivum* L. | Poaceae | MP |
| *Triticum* spp. | Poaceae | HMP |
| *Tropaeolum majus* L. | Tropaeolaceae | P |
| *Tussilago farfara* L. | Asteraceae | HMP |
| *Ulmus minor* Mill. | Ulmaceae | H |
| *Ulmus* spp. | Ulmaceae | HP |
| *Urtica dioica* subsp. *dioica* | Urticaceae | HMP |
| *Vaccinium myrtillus* L. | Ericaceae | HM |
| *Vaccinium uliginosum* L. | Ericaceae | M |
| *Vaccinium vitis-idaea* L. | Ericaceae | H |
| *Valeriana officinalis* L. | Caprifoliaceae | HMP |
| *Valerianella locusta* L. | Caprifoliaceae | HP |
| *Verbascum thapsus* L. | Scrophulariaceae | HM |
| *Verbena officinalis* L. | Verbenaceae | HM |
| *Veronica officinalis* L. | Plantaginaceae | H |
| *Vicia faba* L. | Fabaceae | HP |
| *Vinca minor* L. | Apocynaceae | H |
| *Viola odorata* L. | Violaceae | HMP |
| *Viola tricolor* L. | Violaceae | HM |
| *Viscum album* L. | Santalaceae | HM |
| *Vitis labrusca* L. | Vitaceae | M |
| *Vitis vinifera* L. | Vitaceae | HMP |
| *Zea mays* L. | Poaceae | HMP |
| *Zingiber officinale* Roscoe | Zingiberaceae | H |
| *Ziziphus jujuba* Mill. | Rhamnaceae | P |

**Tab. S2**. List of taxa in the categories Craft, Toxic, Repellent and Insecticide (REP), Veterinary (VET), Games, Other uses and Information (OUI).

| **CRAFT** | **TOXIC** | **REP** | **VET** | **GAME** | **OUI** |
| --- | --- | --- | --- | --- | --- |
| Ulmus minor Mill. | Delphinium staphisagria L. | Lavandula angustifolia subsp. angustifolia | Petroselinum crispum (Mill.) Fuss | Silene vulgaris (Moench) Garcke | Polygonum aviculare L. |
| Celtis australis L. | Rosa canina L. | Equisetum arvense L. | Helleborus foetidus L. | Crataegus monogyna Jacq. | Atropa belladonna L. |
| Spartium junceum L. | Vaccinium uliginosum L. | Rosmarinus officinalis L. | Lavandula angustifolia subsp. angustifolia | Avena sativa L. | Artemisia dracunculus L. |
| Castanea sativa Mill. | Alkekengi officinarum Moench | Nerium oleander L. | Apium graveolens L. | Clematis vitalba L. | Castanea sativa Mill. |
| Quercus petraea subsp. petrea | Atropa belladonna L. | Allium schoenoprasum L. | Fraxinus spp. | Phaseolus vulgaris L. | Helleborus foetidus L. |
| Clematis vitalba L. | Convallaria majalis L. | Allium sativum L. | Calendula officinalis L. | Primula spp. | Clematis vitalba L. |
| Sorbus domestica L. | Euphorbia helioscopia subsp. helioscopia | Sambucus ebulus L. | Helleborus spp. | Arctium lappa L. | Ulmus minor Mill. |
| Cornus mas L. | Lonicera caprifolium L. | Artemisia vulgaris L. | Malva sylvestris L. | Primula veris L. | Mespilus germanica L. |
| Ulmus spp. | Aethusa cynapium L. | Melissa officinalis L. | Hordeum vulgare L. |  | Lavandula angustifolia subsp. angustifolia |
| Euonymus europaeus L. | Mespilus germanica L. | Cistus salviifolius L. | Triticum spp. |  | Phytolacca americana L. |
| Fraxinus excelsior subsp. excelsior |  | Syzygium aromaticum (L.) Merr. & L.M.Perry | Juglans regia L. |  | Hedera helix L. |
| Genista tinctoria L. |  | Euphorbia lathyris L. | Capsella bursa-pastoris Medik. |  | Sambucus nigra L. |
| Taxus baccata L. |  | Ruta graveolens L. | Daucus carota L. |  | Laburnum anagyroidis Medik |
| Hordeum vulgare L. |  | Fraxinus spp. | Fraxinus excelsior subsp. excelsior |  | Silybum marianum (L.) Gaertn. |
| Sambucus nigra L. |  | Solanum lycopersicum L. | Hedera helix L. |  | Pteridium aquilinum (L.) Kuhn |
| Juglans regia L. |  | Juniperus communis L. | Plantago lanceolata L. |  | Taraxacum spp. |
| Sorghum bicolor (L.) Moench |  | Antirrhinum majus L. | Helleborus viridis L. |  | Urtica dioica subsp. dioica |
| Juniperus communis L. |  | Laurus nobilis L. | Populus nigra L. |  | Tussilago farfara L. |
| Triticum aestivum L. |  | Fraxinus ornus subsp. ornus | Mentha spp. |  | Valeriana officinalis L. |
| Morus spp. |  | Lavandula spp. | Quercus spp. |  |  |
| Zea mays L. |  | Mentha spp. | Salix caprea L. |  |  |
| Pyrus communis L. |  | Nicotiana tabacum L. |  |  |  |
| Fagus sylvatica L. |  | Pteridium aquilinum (L.) Kuhn |  |  |  |
| Quercus spp. |  | Ocimum basilicum L. |  |  |  |
| Papaver rhoeas L. |  | Urtica dioica subsp. dioica |  |  |  |
| Robinia pseudoacacia L. |  | Pelargonium spp. |  |  |  |
| Sorbus torminalis (L.) Crantz |  | Aesculus hippocastanum L. |  |  |  |
| Rosa canina L. |  | Pimpinella anisum L. |  |  |  |
| Tilia platyphyllos Scop. |  |  |  |  |  |
| Ruscus aculeatus L. |  |  |  |  |  |
| Cannabis sativa L. |  |  |  |  |  |
| Salix alba L. |  |  |  |  |  |

**Tab S3.** List of taxa for the categories Medicinal, Food, Superstitious-Magical-Religious (SMR), Cosmetic (COSM), Agropastoral (AGROPA), Domestic (DOM) and their subcategories.

| **Category** | **Subcategory** | **List of taxa** |
| --- | --- | --- |
| **MED** | **Gastroenteric** | Achillea millefolium L. ;Aesculus hippocastanum L.;Ajuga chamaepitys (L.) Schreb.;Alkekengi officinarum Moench;Allium cepa L. ;Allium sativum L.;Aloë vera (L.) Burm.f.;Aloysia citrodora Paláu ;Althaea officinalis L.;Angelica archangelica L.;Angelica sylvestris L. ;Apium graveolens L.;Aquilegia spp. ;Arctium lappa L.;Arctostaphylos uva-ursi (L.) Spreng.;Artemisia absinthium L.;Asparagus acutifolius L.;Atropa belladonna L. ;Avena sativa L.;Beta vulgaris L.;Brassica oleracea L.;Buxus sempervirens L.;Calendula officinalis L.;Calluna vulgaris (L.) Hill.;Capsella bursa-pastoris Medik. ;Capsicum annuum L.;Carlina acaulis L. ;Carum carvi L. ;Castanea sativa Mill.;Celtis australis L.;Centaurea spp.;Ceratonia siliqua L.;Cichorium endivia L. ;Cichorium intybus L. ;Cistus salviifolius L.;Citrus limon (L.) Osbeck;Clinopodium nepeta (L.) Kuntze;Colutea arborescens L.;Cornus mas L.;Corylus avellana L.;Crataegus spp.;Cucumis melo L.;Cucurbita pepo L. ;Cynara cardunculus L. ;Cynodon dactylon (L.) Pers. ;Cytisus spp.;Daucus carota L. ;Diospyros kaki L.f. ;Elymus repens subsp. repens;Equisetum arvense L.;Equisetum telmateia Ehrh.;Eruca vesicaria (L.) Cav.;Eupatorium cannabinum L.;Ficus carica L.;Foeniculum vulgare Mill.;Fragaria vesca L. ;Fragaria viridis Weston;Frangula alnus Mill.;Fraxinus excelsior subsp. excelsior;Galanthus nivalis L.;Gentiana lutea L. ;Gentiana spp.;Globularia bisnagarica L.;Glycyrrhiza glabra L.;Helichrysum italicum (Roth) G. Don ;Hordeum murinum L.;Humulus lupulus L.;Hydrangea macrophylla (Thunb.) Ser.;Hypericum perforatum L. ;Hyssopus officinalis L. ;Ilex aquifolium L.;Juglans regia L.;Juniperus communis L.;Lathyrus oleraceus Lam.;Laurus nobilis L.;Lavandula spp.;Linum usitatissimum L.;Lonicera caprifolium L.;Lonicera periclymenum L.;Lupinus albus subsp. albus;Malus domestica (Suckow) Borkh.;Malva sylvestris L. ;Marrubium vulgare L. ;Matricaria chamomilla L.;Medicago sativa L.;Melissa officinalis L.;Mentha spp. ;Mercurialis annua L. ;Mespilus germanica L.;Meum athamanticum Jacq.;Morus spp.;Musa paradisiaca L. ;Narcissus jonquilla L.;Nasturtium officinale R.Br. ;Ocimum basilicum L.;Oenothera biennis L. ;Olea europaea L.;Origanum majorana L.;Origanum vulgare L. ;Oryza sativa L.;Parietaria officinalis L.;Petasites hybridus (L.) G.Gaertn., B.Mey. & Scherb.;Petroselinum crispum (Mill.) Fuss;Phaseolus vulgaris L. ;Pimpinella anisum L.;Pinus spp.;Plantago afra L. ;Plantago lanceolata L.;Plantago major L. ;Plantago spp. ;Polypodium vulgare L. ;Populus alba L.;Portulaca oleracea L. ;Primula ciliata Moretti;Prunus avium L. ;Prunus domestica L. ;Prunus dulcis D.A.Webb;Prunus persica (L.) Batsch ;Prunus spinosa L.;Punica granatum L.;Quercus spp.;Robinia pseudoacacia L.;Rosa canina L.;Rosmarinus officinalis L. ;Rubus idaeus L.;Rubus plicatus Weihe & Nees;Rubus ulmifolius Schott;Rumex acetosa L. ;Rumex alpinus L.;Ruscus aculeatus L.;Ruta graveolens L.;Salix alba L.;Salvia officinalis L.;Salvia sclarea L.;Santolina chamaecyparissus L.;Satureja spp.;Sisymbrium officinale (L.) Scop. ;Solanum dulcamara L.;Soldanella alpina L. ;Sorbus aucuparia L. ;Sorbus domestica L.;Sorbus torminalis (L.) Crantz;Sulla coronaria (L.) ;Syringa vulgaris L.;Syzygium aromaticum (L.) Merr. & L.M.Perry;Tanacetum balsamita L. ;Taraxacum spp.;Teucrium chamaedrys L. ;Thymus vulgaris subsp. vulgaris;Tilia platyphyllos Scop. ;Tilia spp.;Triticum aestivum L.;Triticum spp.;Urtica dioica subsp. dioica;Vaccinium myrtillus L.;Verbena officinalis L.;Veronica officinalis L. ;Viola odorata L.;Viscum album L. ;Vitis vinifera L. |
|  | **Dermatologic** | Abies alba Mill.;Achillea millefolium L. ;Ajuga chamaepitys (L.) Schreb.;Ajuga reptans L.;Alcea rosea L.;Alliaria petiolata (M. Bieb.) Cavara & Grande;Allium cepa L. ;Allium sativum L.;Allium schoenoprasum L.;Alnus glutinosa (L.) Gaertn.;Aloë vera (L.) Burm.f.;Anthyllis vulneraria L. ;Antirrhinum majus L. ;Arctium lappa L.;Barbarea vulgaris W.T.Aiton;Bellis perennis L.;Borago officinalis L.;Brassica oleracea L.;Calendula officinalis L.;Capsella bursa-pastoris Medik. ;Centaurea spp.;Chelidonium majus L.;Cichorium intybus L. ;Cirsium vulgare (Savi) Ten. ;Citrus limon (L.) Osbeck;Corylus avellana L.;Cucumis sativus L. ;Daucus carota L. ;Echinacea angustifolia DC.;Echium vulgare L. ;Elymus repens subsp. repens;Equisetum telmateia Ehrh.;Eupatorium cannabinum L.;Euphorbia cyparissias L. ;Euphorbia helioscopia subsp. helioscopia;Ficus carica L.;Fragaria vesca L. ;Fragaria viridis Weston;Galium sylvaticum L.;Ginkgo biloba L.;Glechoma hederacea L.;Hedera helix L. ;Hepatica nobilis Schreb.;Humulus lupulus L.;Hylotelephium maximum (L.) Holub;Hypericum perforatum L. ;Hyssopus officinalis L. ;Juglans regia L.;Juniperus communis L.;Lactuca sativa L.;Lavandula angustifolia subsp. angustifolia;Leopoldia comosa (L.) Parl. ;Linaria vulgaris subsp. vulgaris;Linum usitatissimum L.;Lonicera caprifolium L.;Lotus corniculatus L.;Lupinus albus subsp. albus;Malva sylvestris L. ;Matricaria chamomilla L.;Medicago sativa L.;Melissa officinalis L.;Mentha spp. ;Narcissus jonquilla L.;Nasturtium officinale R.Br. ;Olea europaea L.;Onobrychis viciifolia Scop. ;Origanum vulgare L. ;Osmunda regalis L. ;Parietaria officinalis L.;Passiflora caerulea L.;Pelargonium spp.;Petasites hybridus (L.) G.Gaertn., B.Mey. & Scherb.;Petrosedum rupestre (L.) P.V.Heath;Petroselinum crispum (Mill.) Fuss;Phedimus spurius (M.Bieb.) 't Hart ;Picea abies (L.) H.Karst.;Plantago lanceolata L.;Plantago major L. ;Plantago spp. ;Polygonum aviculare L. ;Portulaca oleracea L. ;Prunus dulcis D.A.Webb;Pteridium aquilinum (L.) Kuhn ;Ranunculus arvensis L. ;Ranunculus ficaria L.;Ribes nigrum L.;Rosa canina L.;Rosmarinus officinalis L. ;Rubus ulmifolius Schott;Rumex acetosa L. ;Rumex crispus L.;Salix spp.;Salvia officinalis L.;Sambucus nigra L.;Santolina chamaecyparissus L.;Sedum acre L.;Sempervivum montanum L. ;Sempervivum tectorum L.;Solanum lycopersicum L.;Solanum melongena L. ;Solanum tuberosum L. ;Stachys officinalis (L.) Trevis.;Symphytum officinale L. ;Tanacetum balsamita L. ;Taraxacum spp.;Thymus vulgaris subsp. vulgaris;Tilia platyphyllos Scop. ;Trifolium pratense L. ;Trigonella foenum-graecum L.;Triticum spp.;Tussilago farfara L.;Ulmus minor Mill. ;Ulmus spp.;Urtica dioica subsp. dioica;Vaccinium myrtillus L.;Veronica officinalis L. ;Vicia faba L.;Vinca minor L.;Viola odorata L.;Viola tricolor L. ;Vitis vinifera L. |
|  | **Cardiovascular** | Abies alba Mill.;Achillea millefolium L. ;Aesculus hippocastanum L.;Alkekengi officinarum Moench;Allium cepa L. ;Allium sativum L.;Allium schoenoprasum L.;Angelica archangelica L.;Apium graveolens L.;Betula pubescens Ehrh;Brassica oleracea L.;Capsella bursa-pastoris Medik. ;Capsicum annuum L.;Castanea sativa Mill.;Chelidonium majus L.;Citrus limon (L.) Osbeck;Corylus avellana L.;Crataegus laevigata (Poir.) DC.;Crataegus spp.;Crepis vesicaria L. ;Cupressus sempervirens L.;Cynara cardunculus L. ;Cytisus spp.;Elymus repens subsp. repens;Equisetum arvense L.;Equisetum telmateia Ehrh.;Filipendula ulmaria (L.) Maxim.;Fragaria viridis Weston;Galega officinalis L.;Ginkgo biloba L.;Glycyrrhiza glabra L.;Hedera helix L. ;Hypericum perforatum L. ;Juglans regia L.;Laurus nobilis L.;Linaria vulgaris subsp. vulgaris;Malva sylvestris L. ;Medicago sativa L.;Melissa officinalis L.;Mentha spp. ;Nerium oleander L. ;Parietaria officinalis L.;Pelargonium spp.;Petroselinum crispum (Mill.) Fuss;Pilosella officinarum Vaill.;Plantago major L. ;Primula veris L.;Prunus avium L. ;Prunus cerasus L. ;Quercus spp.;Rheum palmatum L.;Rosmarinus officinalis L. ;Rubus ulmifolius Schott;Rumex acetosa L. ;Rumex crispus L.;Ruscus aculeatus L.;Salvia officinalis L.;Sambucus nigra L.;Sedum acre L.;Silybum marianum (L.) Gaertn.;Solanum lycopersicum L.;Tanacetum parthenium (L.) Sch.Bip.;Taraxacum spp.;Thymus vulgaris subsp. vulgaris;Tilia cordata Mill.;Tilia platyphyllos Scop. ;Tilia spp.;Urtica dioica subsp. dioica;Vaccinium myrtillus L.;Vaccinium vitis-idaea L.;Verbascum thapsus L. ;Verbena officinalis L.;Viscum album L. ;Vitis vinifera L. |
|  | **Kidney** | Alkekengi officinarum Moench;Allium cepa L. ;Allium schoenoprasum L.;Arctium lappa L.;Arctostaphylos uva-ursi (L.) Spreng.;Arundo donax L.;Asparagus acutifolius L.;Asplenium ceterach L.;Barbarea vulgaris W.T.Aiton;Bellis perennis L.;Betula pendula Roth;Betula pubescens Ehrh;Borago officinalis L.;Brassica nigra W.D.J.Koch ;Capsella bursa-pastoris Medik. ;Carum carvi L. ;Centaurea calcitrapa L.;Clematis vitalba L.;Crataegus spp.;Cucurbita pepo L. ;Cynara cardunculus L. ;Cynodon dactylon (L.) Pers. ;Cytisus spp.;Elymus repens subsp. repens;Equisetum arvense L.;Equisetum telmateia Ehrh.;Fragaria viridis Weston;Fraxinus excelsior subsp. excelsior;Galium odoratum Scop.;Galium sylvaticum L.;Globularia bisnagarica L.;Glycyrrhiza glabra L.;Hibiscus spp.;Hordeum vulgare L.;Hydrangea macrophylla (Thunb.) Ser.;Ilex aquifolium L.;Juglans regia L.;Juniperus communis L.;Laurus nobilis L.;Lavandula angustifolia subsp. angustifolia;Linum usitatissimum L.;Lonicera periclymenum L.;Malva sylvestris L. ;Matricaria chamomilla L.;Mentha spp. ;Nasturtium officinale R.Br. ;Ocimum basilicum L.;Ostrya carpinifolia Scop.;Oxalis acetosella L.;Paliurus spina-christi Mill. ;Parietaria officinalis L.;Petroselinum crispum (Mill.) Fuss;Plantago lanceolata L.;Primula ciliata Moretti;Prunus avium L. ;Prunus cerasus L. ;Punica granatum L.;Pyrus communis L.;Raphanus raphanistrum subsp. sativus (L.) Domin;Rosmarinus officinalis L. ;Rubus idaeus L.;Ruscus aculeatus L.;Salvia officinalis L.;Sambucus nigra L.;Spartium junceum L.;Taraxacum spp.;Triticum aestivum L.;Urtica dioica subsp. dioica;Vaccinium myrtillus L.;Vaccinium vitis-idaea L.;Vinca minor L.;Viscum album L. ;Zea mays L. |
|  | **Oral cavity** | Alcea rosea L.;Allium cepa L. ;Allium sativum L.;Aloysia citrodora Paláu ;Althaea officinalis L.;Arctium lappa L.;Avena sativa L.;Capsicum annuum L.;Castanea sativa Mill.;Citrus aurantium L.;Coffea arabica L.;Coriandrum sativum L. ;Cornus sanguinea L. ;Cytisus scoparius subsp. scoparius;Daucus carota L. ;Equisetum arvense L.;Foeniculum vulgare Mill.;Hedera helix L. ;Hordeum murinum L.;Hyoscyamus niger L. ;Lactuca sativa L.;Laurus nobilis L.;Lonicera caprifolium L.;Malva sylvestris L. ;Matricaria chamomilla L.;Melissa officinalis L.;Mentha spp. ;Mespilus germanica L.;Ocimum basilicum L.;Ononis spinosa L.;Parietaria officinalis L.;Pelargonium spp.;Petroselinum crispum (Mill.) Fuss;Plantago lanceolata L.;Punica granatum L.;Quercus spp.;Robinia pseudoacacia L.;Rosa canina L.;Rosmarinus officinalis L. ;Rubus idaeus L.;Rubus ulmifolius Schott;Rumex acetosa L. ;Salvia officinalis L.;Salvia pratensis L.;Sambucus nigra L.;Satureja spp.;Silybum marianum (L.) Gaertn.;Solanum tuberosum L. ;Syzygium aromaticum (L.) Merr. & L.M.Perry;Thymus vulgaris subsp. vulgaris;Urtica dioica subsp. dioica;Vaccinium myrtillus L.;Vitis vinifera L. |
|  | **Musculoskeletal** | Acorus calamus L.;Ajuga reptans L.;Allium ampeloprasum L.;Allium cepa L. ;Allium sativum L.;Aloë vera (L.) Burm.f.;Asparagus acutifolius L.;Bellis perennis L.;Betula pendula Roth;Brassica oleracea L.;Capsicum annuum L.;Cichorium intybus L. ;Citrus limon (L.) Osbeck;Clematis vitalba L.;Cupressus sempervirens L.;Cynodon dactylon (L.) Pers. ;Cytisus scoparius subsp. scoparius;Equisetum arvense L.;Equisetum telmateia Ehrh.;Hedera helix L. ;Helichrysum stoechas (L.) Moench;Hyoscyamus niger L. ;Hypericum perforatum L. ;Ilex aquifolium L.;Iris spp.;Lavandula angustifolia subsp. angustifolia;Lilium candidum L. ;Linum usitatissimum L.;Malva sylvestris L. ;Matricaria chamomilla L.;Medicago sativa L.;Mentha spp. ;Musa paradisiaca L. ;Petasites hybridus (L.) G.Gaertn., B.Mey. & Scherb.;Phaseolus vulgaris L. ;Picea abies (L.) H.Karst.;Pinus sylvestris L.;Primula ciliata Moretti;Primula veris L.;Prunus avium L. ;Prunus dulcis D.A.Webb;Pteridium aquilinum (L.) Kuhn ;Ribes nigrum L.;Rosmarinus officinalis L. ;Ruscus aculeatus L.;Ruta graveolens L.;Salvia officinalis L.;Sambucus nigra L.;Saxifraga spp. ;Taraxacum spp.;Urtica dioica subsp. dioica;Verbena officinalis L.;Viola odorata L. |
|  | **Nervous system** | Allium sativum L.;Aloysia citrodora Paláu ;Aquilegia spp. ;Atropa belladonna L. ;Avena sativa L.;Chamaemelum nobile L.;Convallaria majalis L. ;Crataegus laevigata (Poir.) DC.;Crataegus spp.;Cytisus spp.;Eruca vesicaria (L.) Cav.;Galanthus nivalis L.;Ginkgo biloba L.;Hedera helix L. ;Hibiscus spp.;Humulus lupulus L.;Hydrangea macrophylla (Thunb.) Ser.;Hyoscyamus niger L. ;Hypericum perforatum L. ;Juglans regia L.;Laurus nobilis L.;Lavandula angustifolia subsp. angustifolia;Malva sylvestris L. ;Matricaria chamomilla L.;Melissa officinalis L.;Mentha spp. ;Myosotis arvensis Hill ;Narcissus jonquilla L.;Ocimum basilicum L.;Origanum majorana L.;Papaver rhoeas L. ;Passiflora caerulea L.;Pelargonium spp.;Primula ciliata Moretti;Prunus persica (L.) Batsch ;Rosmarinus officinalis L. ;Salix alba L.;Salvia officinalis L.;Sambucus nigra L.;Stachys recta L.;Tanacetum parthenium (L.) Sch.Bip.;Tilia cordata Mill.;Tilia platyphyllos Scop. ;Tilia spp.;Tussilago farfara L.;Valeriana officinalis L.;Viscum album L. |
|  | **Hepatic** | Agrimonia eupatoria L. ;Allium cepa L. ;Angelica sylvestris L. ;Arctium lappa L.;Asparagus acutifolius L.;Bellis perennis L.;Beta vulgaris L.;Betula pendula Roth;Borago officinalis L.;Brassica oleracea L.;Centaurea spp.;Cichorium intybus L. ;Corylus avellana L.;Cynara cardunculus L. ;Galium odoratum Scop.;Gentiana spp.;Helichrysum stoechas (L.) Moench;Hepatica nobilis Schreb.;Hibiscus spp.;Hydrangea macrophylla (Thunb.) Ser.;Juniperus communis L.;Levisticum officinale W.D.J. Koch;Linaria vulgaris subsp. vulgaris;Malva sylvestris L. ;Matricaria chamomilla L.;Medicago sativa L.;Melissa officinalis L.;Mentha spp. ;Onobrychis viciifolia Scop. ;Papaver rhoeas L. ;Petroselinum crispum (Mill.) Fuss;Plantago lanceolata L.;Plantago spp. ;Prunus avium L. ;Prunus domestica L. ;Rheum palmatum L.;Rosmarinus officinalis L. ;Rumex acetosa L. ;Rumex alpinus L.;Ruscus aculeatus L.;Silybum marianum (L.) Gaertn.;Sonchus spp.;Taraxacum spp.;Urtica dioica subsp. dioica;Vaccinium myrtillus L.;Viola tricolor L. ;Zea mays L. |
|  | **Febrifuge** | Allium cepa L. ;Aloysia citrodora Paláu ;Asplenium viride Huds.;Borago officinalis L.;Buxus sempervirens L.;Capsella bursa-pastoris Medik. ;Castanea sativa Mill.;Centaurea spp.;Centaurium erythraea Rafn;Cornus mas L.;Euphorbia helioscopia subsp. helioscopia;Fagus sylvatica L. ;Fraxinus excelsior subsp. excelsior;Gentiana lutea L. ;Ilex aquifolium L.;Lonicera caprifolium L.;Malus domestica (Suckow) Borkh.;Mespilus germanica L.;Parietaria officinalis L.;Populus nigra L. ;Prunus armeniaca L. ;Prunus avium L. ;Prunus dulcis D.A.Webb;Prunus spinosa L.;Pulmonaria officinalis L.;Punica granatum L.;Rumex alpinus L.;Ruscus aculeatus L.;Salix alba L.;Salvia officinalis L.;Sambucus nigra L.;Thymus vulgaris subsp. vulgaris;Tilia platyphyllos Scop. ;Valeriana officinalis L.;Verbascum thapsus L. ;Verbena officinalis L.;Zingiber officinale Roscoe |
|  | **Reproductive system** | Adiantum capillus-veneris L. ;Ajuga chamaepitys (L.) Schreb.;Calendula officinalis L.;Capsella bursa-pastoris Medik. ;Centaurea spp.;Chamaemelum nobile L.;Cynodon dactylon (L.) Pers. ;Equisetum arvense L.;Foeniculum vulgare Mill.;Galega officinalis L.;Glycyrrhiza glabra L.;Hedera helix L. ;Humulus lupulus L.;Hypericum perforatum L. ;Juglans regia L.;Lactuca sativa L.;Malva sylvestris L. ;Matricaria chamomilla L.;Medicago sativa L.;Origanum vulgare L. ;Osmunda regalis L. ;Parietaria officinalis L.;Petroselinum crispum (Mill.) Fuss;Portulaca oleracea L. ;Rosmarinus officinalis L. ;Rubus ulmifolius Schott;Salix alba L.;Salvia officinalis L.;Tanacetum parthenium (L.) Sch.Bip.;Trifolium pratense L. ;Urtica dioica subsp. dioica;Vinca minor L. |
|  | **Antiparasitic** | Ailanthus altissima (Mill.) Swingle ;Allium cepa L. ;Allium sativum L.;Allium schoenoprasum L.;Angelica sylvestris L. ;Clematis vitalba L.;Cucurbita maxima Duchesne ;Cucurbita pepo L. ;Delphinium consolida (L.);Delphinium staphisagria L. ;Dryopteris filix-mas (L.) Schott;Euonymus europaeus L. ;Euphorbia helioscopia subsp. helioscopia;Gentiana lutea L. ;Juglans regia L.;Lavandula angustifolia subsp. angustifolia;Lupinus albus subsp. albus;Olea europaea L.;Petroselinum crispum (Mill.) Fuss;Pimpinella anisum L.;Polypodium vulgare L. ;Prunus armeniaca L. ;Prunus domestica L. ;Prunus dulcis D.A.Webb;Prunus persica (L.) Batsch ;Punica granatum L.;Rosa canina L.;Ruta graveolens L.;Tanacetum balsamita L. ;Thymus vulgaris subsp. vulgaris |
|  | **Ophthalmic** | Alcea rosea L.;Calendula officinalis L.;Centaurea cyanus L.;Centaurea spp.;Daucus carota L. ;Euphrasia officinalis L.;Foeniculum vulgare Mill.;Geranium robertianum L.;Hordeum murinum L.;Matricaria chamomilla L.;Melilotus officinalis Pall.;Myosotis arvensis Hill ;Olea europaea L.;Petroselinum crispum (Mill.) Fuss;Plantago lanceolata L.;Rosa canina L.;Rosa gallica L. ;Sambucus nigra L.;Sempervivum montanum L. ;Solanum tuberosum L. ;Stachys recta L.;Vaccinium myrtillus L.;Viola odorata L.;Vitis vinifera L. |
|  | **General state of health** | Aloë vera (L.) Burm.f.;Avena sativa L.;Gentiana lutea L. ;Lavandula spp.;Linum usitatissimum L.;Malus domestica (Suckow) Borkh.;Malva sylvestris L. ;Medicago sativa L.;Musa paradisiaca L. ;Ocimum basilicum L.;Plantago lanceolata L.;Prunus domestica L. ;Rosa canina L.;Rosmarinus officinalis L. ;Rubus idaeus L.;Salvia sclarea L.;Taraxacum spp.;Tilia platyphyllos Scop. ;Valeriana officinalis L.;Veronica officinalis L. ;Vitis vinifera L. |
|  | **Anti-inflammatory** | Aquilegia spp. ;Betula pendula Roth;Calendula officinalis L.;Citrus limon (L.) Osbeck;Corylus avellana L.;Hordeum murinum L.;Linum usitatissimum L.;Plantago lanceolata L.;Primula ciliata Moretti;Quercus spp.;Ruscus aculeatus L.;Salix alba L.;Salix spp.;Salvia officinalis L. |
|  | **Ears** | Allium sativum L.;Ballota nigra L.;Crataegus spp.;Cyclamen hederifolium Aiton ;Malva sylvestris L. ;Matricaria chamomilla L.;Olea europaea L.;Prunus armeniaca L. ;Ruta graveolens L.;Sambucus nigra L. |
|  | **Afrodisiac and sexual desire regulator** | Melissa officinalis L.;Portulaca oleracea L. ;Salix alba L.;Salvia officinalis L.; |
|  | **Antibiotic** | Calendula officinalis L.;Origanum vulgare L. ; |
|  | **Antitumoral** | Aloë vera (L.) Burm.f. |
| **FOOD** | **Nutraceutical** | Achillea millefolium L. ;Ajuga chamaepitys (L.) Schreb.;Allium ampeloprasum L.;Allium cepa L. ;Aloysia citrodora Paláu ;Anethum graveolens L.;Apium graveolens L.;Arbutus unedo L.;Arctium lappa L.;Artemisia dracunculus L.;Barbarea vulgaris W.T.Aiton;Bellis perennis L.;Brassica napus L. ;Brassica oleracea L.;Capsella bursa-pastoris Medik. ;Capsicum annuum L.;Carlina acaulis L. ;Castanea sativa Mill.;Celtis australis L.;Ceratonia siliqua L.;Chenopodium bonus-henricus L. ;Cichorium endivia L. ;Cichorium intybus L. ;Citrus limon (L.) Osbeck;Clematis vitalba L.;Cornus mas L.;Corylus avellana L.;Crataegus monogyna Jacq.;Crataegus spp.;Crepis vesicaria L. ;Cucumis sativus L. ;Cynara cardunculus L. ;Dipsacus laciniatus L.;Elymus repens subsp. repens;Equisetum arvense L.;Equisetum telmateia Ehrh.;Ficus carica L.;Foeniculum vulgare Mill.;Fragaria vesca L. ;Fragaria viridis Weston;Helianthus tuberosus L.;Hippophae rhamnoides L.;Hordeum murinum L.;Hordeum vulgare L.;Humulus lupulus L.;Juglans regia L.;Lactuca sativa L.;Leopoldia comosa (L.) Parl. ;Malus domestica (Suckow) Borkh.;Malva sylvestris L. ;Matricaria chamomilla L.;Melissa officinalis L.;Mentha spp. ;Mespilus germanica L.;Nasturtium officinale R.Br. ;Olea europaea L.;Ostrya carpinifolia Scop.;Oxalis acetosella L.;Phaseolus vulgaris L. ;Pimpinella anisum L.;Pinus sylvestris L.;Polypodium vulgare L. ;Portulaca oleracea L. ;Prunus armeniaca L. ;Prunus avium L. ;Prunus cerasifera Ehrh. ;Prunus cerasus L. ;Prunus domestica L. ;Prunus persica (L.) Batsch ;Prunus spinosa L.;Punica granatum L.;Pyrus communis L.;Rosa canina L.;Rosmarinus officinalis L. ;Rubus idaeus L.;Rumex acetosa L. ;Ruscus aculeatus L.;Ruta graveolens L.;Salvia officinalis L.;Sambucus nigra L.;Sanguisorba minor Scop.;Silybum marianum (L.) Gaertn.;Solanum tuberosum L. ;Sonchus spp.;Sorbus domestica L.;Sorbus torminalis (L.) Crantz;Spinacia oleracea L.;Sulla coronaria (L.) ;Symphytum officinale L. ;Taraxacum spp.;Tragopogon pratensis L.;Triticum spp.;Urtica dioica subsp. dioica;Vaccinium myrtillus L.;Valeriana officinalis L.;Valerianella locusta L. ;Verbena officinalis L.;Vitis labrusca L. |
|  | **Dishes** | Alkekengi officinarum Moench;Allium ampeloprasum L.;Allium cepa L. ;Allium sativum L.;Apium graveolens L.;Arctium lappa L.;Armoracia rusticana G. Gaertn., B.Mey. & Scherb.;Borago officinalis L.;Calendula officinalis L.;Capsella bursa-pastoris Medik. ;Capsicum annuum L.;Carum carvi L. ;Ceratonia siliqua L.;Cichorium intybus L. ;Clematis vitalba L.;Clinopodium nepeta (L.) Kuntze;Convolvulus arvensis L.;Coriandrum sativum L. ;Corylus avellana L.;Crocus sativus L.;Cucurbita maxima Duchesne ;Cucurbita pepo L. ;Cupressus sempervirens L.;Cydonia oblonga Mill.;Cynara cardunculus L. ;Daucus carota L. ;Equisetum arvense L.;Foeniculum vulgare Mill.;Fragaria vesca L. ;Fragaria viridis Weston;Glycyrrhiza glabra L.;Helianthus tuberosus L.;Juglans regia L.;Juniperus communis L.;Leopoldia comosa (L.) Parl. ;Mespilus germanica L.;Origanum majorana L.;Papaver rhoeas L. ;Petroselinum crispum (Mill.) Fuss;Pinus pinea L. ;Plantago lanceolata L.;Primula vulgaris Huds.;Prunus avium L. ;Prunus dulcis D.A.Webb;Prunus persica (L.) Batsch ;Prunus spinosa L.;Punica granatum L.;Raphanus raphanistrum subsp. sativus (L.) Domin;Robinia pseudoacacia L.;Rosa canina L.;Rubus ulmifolius Schott;Ruscus aculeatus L.;Salvia officinalis L.;Sambucus nigra L.;Sanguisorba minor Scop.;Satureja spp.;Silene vulgaris (Moench) Garcke ;Solanum melongena L. ;Solanum tuberosum L. ;Symphytum officinale L. ;Syzygium aromaticum (L.) Merr. & L.M.Perry;Taraxacum spp.;Vaccinium myrtillus L.;Vaccinium vitis-idaea L.;Viola odorata L. |
|  | **Eaten raw** | Achillea millefolium L. ;Aloysia citrodora Paláu ;Angelica sylvestris L. ;Bellis perennis L.;Borago officinalis L.;Campanula rapunculus L.;Cichorium intybus L. ;Clematis vitalba L.;Cornus mas L.;Crataegus laevigata (Poir.) DC.;Crepis sancta L. Babc. ;Crepis vesicaria L. ;Cynara cardunculus L. ;Dactylis glomerata L.;Diplotaxis tenuifolia (L.) DC. ;Eruca vesicaria (L.) Cav.;Fagus sylvatica L. ;Ficus carica L.;Foeniculum vulgare Mill.;Glycyrrhiza glabra L.;Lactuca sativa L.;Leucanthemum vulgare subsp. vulgare;Linaria vulgaris subsp. vulgaris;Medicago sativa L.;Mespilus germanica L.;Morus nigra L.;Nasturtium officinale R.Br. ;Oxalis acetosella L.;Paliurus spina-christi Mill. ;Papaver rhoeas L. ;Portulaca oleracea L. ;Primula ciliata Moretti;Primula spp.;Primula veris L.;Prunus cerasifera Ehrh. ;Pyrus communis L.;Rosa canina L.;Rubus ulmifolius Schott;Rumex acetosa L. ;Sanguisorba minor Scop.;Sempervivum montanum L. ;Silene vulgaris (Moench) Garcke ;Silybum marianum (L.) Gaertn.;Sinapis alba L. ;Sonchus spp.;Sorbus domestica L.;Spinacia oleracea L.;Tanacetum balsamita L. ;Taraxacum spp.;Tragopogon pratensis L.;Tropaeolum majus L.;Tussilago farfara L.;Vaccinium myrtillus L.;Valeriana officinalis L.;Valerianella locusta L. |
|  | **Spices, aroma, flavour** | Allium cepa L. ;Allium sativum L.;Allium schoenoprasum L.;Aloysia citrodora Paláu ;Anacamptis morio (L.) R.M.Bateman, Pridgeon & M.W.Chase;Anethum graveolens L.;Artemisia dracunculus L.;Bellis perennis L.;Capsicum annuum L.;Citrus limon (L.) Osbeck;Clinopodium nepeta (L.) Kuntze;Cornus mas L.;Eruca vesicaria (L.) Cav.;Foeniculum vulgare Mill.;Juglans regia L.;Juniperus communis L.;Lamium amplexicaule L. ;Laurus nobilis L.;Lavandula angustifolia subsp. angustifolia;Lonicera periclymenum L.;Melissa officinalis L.;Mentha spp. ;Ocimum basilicum L.;Origanum majorana L.;Origanum vulgare L. ;Papaver rhoeas L. ;Persicaria hydropiper (L.) Delarbre;Petroselinum crispum (Mill.) Fuss;Polypodium vulgare L. ;Primula vulgaris Huds.;Punica granatum L.;Rosa gallica L. ;Rosmarinus officinalis L. ;Rumex acetosa L. ;Ruta graveolens L.;Salvia officinalis L.;Salvia sclarea L.;Sambucus nigra L.;Stevia rebaudiana Bertoni;Symphytum officinale L. ;Thymus vulgaris subsp. vulgaris;Vitis vinifera L. ;Zingiber officinale Roscoe |
|  | **Liquor** | Achillea millefolium L. ;Aloysia citrodora Paláu ;Angelica archangelica L.;Arctium lappa L.;Artemisia vulgaris L.;Avena sativa L.;Centaurium erythraea Rafn;Citrus limon (L.) Osbeck;Cynara cardunculus L. ;Foeniculum vulgare Mill.;Galium odoratum Scop.;Gentiana lutea L. ;Humulus lupulus L.;Juglans regia L.;Juniperus communis L.;Laurus nobilis L.;Malus domestica (Suckow) Borkh.;Malva sylvestris L. ;Matricaria chamomilla L.;Melissa officinalis L.;Mentha spp. ;Ocimum basilicum L.;Pinus mugo Turra;Prunus cerasus L. ;Prunus laurocerasus L.;Prunus persica (L.) Batsch ;Prunus spinosa L.;Pyrus communis L.;Robinia pseudoacacia L.;Rosa canina L.;Rosa gallica L. ;Rosa spp. ;Rubus ulmifolius Schott;Ruta graveolens L.;Salvia officinalis L.;Salvia pratensis L.;Sambucus nigra L.;Satureja spp.;Sorbus torminalis (L.) Crantz;Teucrium chamaedrys L. ;Vaccinium myrtillus L.;Verbena officinalis L. |
|  | **Eaten cooked** | Achillea millefolium L. ;Allium ampeloprasum L.;Allium schoenoprasum L.;Asparagus acutifolius L.;Avena sativa L.;Beta vulgaris L.;Castanea sativa Mill.;Cichorium intybus L. ;Cirsium vulgare (Savi) Ten. ;Clematis vitalba L.;Cornus mas L.;Equisetum arvense L.;Helianthus tuberosus L.;Humulus lupulus L.;Nasturtium officinale R.Br. ;Phaseolus vulgaris L. ;Pulmonaria officinalis L.;Robinia pseudoacacia L.;Sinapis alba L. ;Sinapis arvensis L.;Sonchus spp.;Taraxacum spp.;Vicia faba L. |
|  | **Jam** | Alkekengi officinarum Moench;Arbutus unedo L.;Cornus mas L.;Corylus avellana L.;Ficus carica L.;Fragaria vesca L. ;Mespilus germanica L.;Morus alba L.;Morus spp.;Prunus avium L. ;Prunus cerasifera Ehrh. ;Prunus cerasus L. ;Prunus domestica L. ;Prunus spinosa L.;Rosa canina L.;Rosa spp. ;Rubus ulmifolius Schott;Sambucus nigra L.;Sorbus domestica L.;Sorbus torminalis (L.) Crantz;Vaccinium myrtillus L. |
|  | **Traditional dishes** | Allium sativum L.;Borago officinalis L.;Brassica nigra W.D.J.Koch ;Castanea sativa Mill.;Ficus carica L.;Foeniculum vulgare Mill.;Primula spp.;Prunus persica (L.) Batsch ;Sambucus nigra L.;Silene vulgaris (Moench) Garcke ;Spinacia oleracea L.;Triticum spp.;Urtica dioica subsp. dioica;Vitis vinifera L. ;Zea mays L. |
|  | **Appetite regulator** | Aloysia citrodora Paláu ;Eruca vesicaria (L.) Cav.;Fagus sylvatica L. ;Humulus lupulus L.;Nasturtium officinale R.Br. ;Origanum majorana L.;Origanum vulgare L. ;Polypodium vulgare L. ;Rumex acetosa L. ;Silybum marianum (L.) Gaertn.;Verbena officinalis L.; |
|  | **Alternative to coffee** | Cichorium intybus L. ;Fagus sylvatica L. ;Hordeum murinum L.;Hordeum vulgare L.;Quercus pubescens subsp. pubescens;Quercus robur L. ;Ruscus aculeatus L.;Sonchus spp.;Taraxacum spp.; |
|  | **Thirst quencher** | Cucumis sativus L. ;Mentha spp. ;Polypodium vulgare L. ;Robinia pseudoacacia L.;Sambucus nigra L.;Urtica dioica subsp. dioica;; |
|  | **Drinks** | Citrus limon (L.) Osbeck;Cucumis sativus L. ;Mentha spp. ;Prunus avium L. ;Rosa canina L.; |
|  | **Milk curding** | Erigeron canadensis L.;Galium sylvaticum L.;Galium verum L. ;Laurus nobilis L.; |
|  | **Preservative** | Achillea millefolium L. ;Artemisia dracunculus L.;Mentha spp. ;Verbascum thapsus L. ;; |
|  | **Flour** | Castanea sativa Mill.;Cynodon dactylon (L.) Pers. ;Pteridium aquilinum (L.) Kuhn ;Triticum spp.; |
|  | **Hot beverages** | Achillea millefolium L. ;Fumaria officinalis L.;Symphytum officinale L. ;; |
| **SMR** | **Healer** | Dryopteris filix-mas (L.) Schott;Humulus lupulus L.;Hypericum perforatum L. ;Juglans regia L.;Lavandula spp.;Rosa canina L.;Rubus ulmifolius Schott;Ruta graveolens L.;Sambucus nigra L.;Saxifraga spp. ;Ajuga chamaepitys (L.) Schreb.;Raphanus raphanistrum subsp. sativus (L.) Domin;Sorbus aucuparia L. ;Stachys recta L.;Vitis vinifera L. |
|  | **Propitiatory** | Aesculus hippocastanum L.;Buxus sempervirens L.;Juglans regia L.;Olea europaea L.;Oxalis acetosella L.;Passiflora caerulea L.;Populus alba L.;Sambucus nigra L.;Taraxacum spp.;Trifolium repens L.;Pteridium aquilinum (L.) Kuhn ;Sempervivum tectorum L.;Trifolium pratense L. ;Urtica dioica subsp. dioica; |
|  | **Other SMR** | Acer campestre L.;Cupressus sempervirens L.;Salix spp.;Sambucus nigra L.;Sanguinaria canadensis L.;Taraxacum spp.;Lavandula angustifolia subsp. angustifolia;Antirrhinum majus L. ;Linaria vulgaris subsp. vulgaris;Mentha spp. ;Pteridium aquilinum (L.) Kuhn ;Sempervivum montanum L. ;Urtica dioica subsp. dioica;; |
|  | **Apotropaic** | Acer campestre L.;Agrimonia eupatoria L. ;Allium sativum L.;Hypericum perforatum L. ;Ilex aquifolium L.;Juglans regia L.;Juniperus communis L.;Robinia pseudoacacia L.;Ruscus aculeatus L.;Ruta graveolens L.;Salvia officinalis L.;Achillea millefolium L. ;Crataegus spp.; |
|  | **Protective from desease** | Aesculus hippocastanum L.;Allium sativum L.;Calendula officinalis L.;Clematis vitalba L.;Convallaria majalis L. ;Echium vulgare L. ;Hedera helix L. ;Sempervivum montanum L. ;; |
|  | **Peace, good mood** | Calendula officinalis L.;Olea europaea L.;Punica granatum L.;Lavandula angustifolia subsp. angustifolia;;; |
|  | **Weather prediction** | Allium cepa L. ;Taraxacum spp.; |
| **COSM** | **Skin treatment** | Aesculus hippocastanum L.;Arctium lappa L.;Borago officinalis L.;Castanea sativa Mill.;Chamaemelum nobile L.;Citrus limon (L.) Osbeck;Cornus mas L.;Cucumis sativus L. ;Cucurbita maxima Duchesne ;Daucus carota L. ;Diospyros kaki L.f. ;Fragaria vesca L. ;Fragaria viridis Weston;Juglans regia L.;Juniperus communis L.;Lavandula angustifolia subsp. angustifolia;Linum usitatissimum L.;Lonicera caprifolium L.;Malva sylvestris L. ;Matricaria chamomilla L.;Papaver rhoeas L. ;Plantago spp. ;Populus nigra L. ;Primula veris L.;Prunus dulcis D.A.Webb;Rosa canina L.;Rosa spp. ;Rosmarinus officinalis L. ;Rubus ulmifolius Schott;Salvia officinalis L.;Sempervivum montanum L. ;Solanum dulcamara L.;Sorbus domestica L.;Taraxacum spp.;Thymus vulgaris subsp. vulgaris;Tilia platyphyllos Scop. ;Tussilago farfara L. |
|  | **Hair-Scalp** | Adiantum capillus-veneris L. ;Allium sativum L.;Angelica sylvestris L. ;Arctium lappa L.;Castanea sativa Mill.;Centaurea spp.;Citrus limon (L.) Osbeck;Equisetum telmateia Ehrh.;Hedera helix L. ;Hibiscus spp.;Juglans regia L.;Lavandula angustifolia subsp. angustifolia;Linum usitatissimum L.;Matricaria chamomilla L.;Olea europaea L.;Rosmarinus officinalis L. ;Salvia officinalis L.;Saponaria officinalis L.;Spartium junceum L.;Thymus vulgaris subsp. vulgaris;Urtica dioica subsp. dioica;Vitis vinifera L. |
|  | **Other cosmetic** | Brassica oleracea L.;Coffea arabica L.;Convallaria majalis L. ;Cucumis sativus L. ;Juglans regia L.;Laurus nobilis L.;Lavandula angustifolia subsp. angustifolia;Lonicera caprifolium L.;Matricaria chamomilla L.;Melissa officinalis L.;Mentha spp. ;Prunus spinosa L.;Quercus spp.;Rosa canina L.;Rosa gallica L. ;Ruscus aculeatus L.;Salvia officinalis L.;Salvia pratensis L.;Tanacetum balsamita L. ;Tilia platyphyllos Scop. ;Tilia spp. |
|  | **Cellulitis** | Achillea millefolium L. ;Betula pendula Roth;Betula pubescens Ehrh;Filipendula ulmaria (L.) Maxim.;Hedera helix L. ;Helichrysum italicum (Roth) G. Don ;Pelargonium spp.;Pilosella officinarum Vaill.;Rosmarinus officinalis L. ;Ruscus aculeatus L.;Taraxacum spp.;Valeriana officinalis L. |
| **AGROPA** | **Feed** | Achillea millefolium L. ;Anthyllis vulneraria L. ;Beta vulgaris L.;Ceratonia siliqua L.;Cynodon dactylon (L.) Pers. ;Dactylis glomerata L.;Dipsacus fullonum L.;Elymus repens subsp. repens;Euphorbia spp.;Laburnum anagyroidis Medik;Lotus corniculatus L.;Medicago sativa L.;Morus alba L.;Morus spp.;Onobrychis viciifolia Scop. ;Papaver rhoeas L. ;Pimpinella anisum L.;Plantago spp. ;Quercus robur L. ;Quercus spp.;Salix caprea L.;Solanum tuberosum L. ;Sulla coronaria (L.) ;Triticum aestivum L.;Tussilago farfara L.;Ulmus spp.;Urtica dioica subsp. dioica;Vicia faba L.;Zea mays L. |
|  | **Horticulture** | Arctium lappa L.;Centaurea calcitrapa L.;Equisetum arvense L.;Papaver rhoeas L. ;Petasites hybridus (L.) G.Gaertn., B.Mey. & Scherb.;Solanum tuberosum L. ;Symphytum officinale L. ;Trifolium pratense L. ;Ulmus spp.;Urtica dioica subsp. dioica;Vicia faba L. |
|  | **Other agropastoral** | Castanea sativa Mill.;Hippophae rhamnoides L.;Hordeum vulgare L.;Ilex aquifolium L.;Lavandula angustifolia subsp. angustifolia;Medicago sativa L.;Robinia pseudoacacia L.;Sempervivum montanum L. ;Tilia platyphyllos Scop. ;Triticum aestivum L.;Urtica dioica subsp. dioica;; |
| **DOM** | **Other domestic** | Arctium minus (Hill) Bernh.;Calluna vulgaris (L.) Hill.;Castanea sativa Mill.;Chenopodium bonus-henricus L. ;Corylus avellana L.;Dipsacus laciniatus L.;Equisetum arvense L.;Euphorbia spp.;Ficus carica L.;Hordeum vulgare L.;Juniperus communis L.;Linum usitatissimum L.;Oxalis acetosella L.;Parietaria officinalis L.;Prunus cerasus L. ;Sambucus nigra L.;Saponaria officinalis L.;Triticum aestivum L.;Triticum spp.;Urtica dioica subsp. dioica;Vitis labrusca L.;Zea mays L. |
|  | **Fuel** | Cannabis sativa L.;Crataegus spp.;Fagus sylvatica L. ;Juglans regia L.;Medicago sativa L.;Ostrya carpinifolia Scop.;Robinia pseudoacacia L.;Triticum spp.;Ulmus spp.;Verbascum thapsus L. ;Vitis vinifera L. ;Zea mays L. |
|  | **Perfume** | Aloysia citrodora Paláu ;Juniperus communis L.;Laurus nobilis L.;Lavandula angustifolia subsp. angustifolia;Lavandula spp.;Melissa officinalis L.;Rosa canina L.;Syzygium aromaticum (L.) Merr. & L.M.Perry;Tanacetum corymbosum (L.) Sch.Bip.;Viola tricolor L. |
|  | **Dye** | Cornus mas L.;Cynara cardunculus L. ;Gentiana spp.;Prunus spinosa L.;Quercus pubescens subsp. pubescens;Rosa canina L. |
|  | **Ornamental** | Hydrangea macrophylla (Thunb.) Ser.;Lavandula angustifolia subsp. angustifolia;Punica granatum L.;Syringa vulgaris L.;Verbena officinalis L.;Viscum album L. ; |
|  | **Ties** | Colutea arborescens L.;Cytisus scoparius subsp. scoparius;Rubus ulmifolius Schott;Salix caprea L.; |
|  | **Ink** | Ribes nigrum L.;Sambucus nigra L.;Vaccinium myrtillus L.;; |

**Table S4.** Most cited taxa for MED subcategories. For each subcategory the total citations number in the overall MED category is reported in brackets and the three most cited taxa are listed giving the number of citations for the specific MED-subcategory.

| MED-sub and total citation in MED UC (in brackets) | Most cited taxa in the sub. | N. of citations |
| --- | --- | --- |
| Afrodisiac and sexual desire regulator (5) | *Portulaca oleracea* L. | 2 |
|  | *Melissa officinalis* L. | 1 |
|  | *Salix alba* L. | 1 |
|  | *Salvia officinalis* L. | 1 |
| Antibiotic (3) | *Origanum vulgare* L. | 2 |
|  | *Calendula officinalis* L. | 1 |
| Anti-inflammatory (18) | *Corylus avellana* L. | 3 |
|  | *Quercus* spp. | 2 |
|  | *Salix* spp. | 2 |
| Antiparasitic (82) | *Allium sativum* L. | 19 |
|  | *Punica granatum* L. | 10 |
|  | *Lavandula angustifolia* subsp. *angustifolia* | 5 |
|  | *Prunus armeniaca* L. | 5 |
| Antitumoral (1) | *Aloe vera* (L.) Burm.f. | 1 |
| Cardiovascular (342) | *Allium sativum* L. | 51 |
|  | *Rosmarinus officinalis* L. | 19 |
|  | *Urtica dioica* subsp. *dioica* | 14 |
| Dermatologic (716) | *Ficus carica* L. | 47 |
|  | *Hypericum perforatum* L. | 35 |
|  | *Chelidonium majus* L. | 34 |
| Ears (33) | *Olea europaea* L. | 18 |
|  | *Allium sativum* L. | 4 |
|  | *Cyclamen hederifolium* Aiton | 2 |
|  | *Ruta graveolens* L. | 2 |
|  | *Sambucus nigra* L. | 2 |
| Febrifuge (75) | *Centaurium erythraea* Rafn | 7 |
|  | *Pulmonaria officinalis* L. | 5 |
|  | *Borago officinalis* L. | 5 |
| Gastroenteric (863) | *Malva sylvestris* L. | 74 |
|  | *Matricaria chamomilla* L. | 37 |
|  | *Melissa officinalis* L. | 33 |
| General state of health (42) | *Rosa canina* L. | 10 |
|  | *Vitis vinifera* L. | 5 |
|  | *Medicago sativa* L. | 5 |
| Hepatic (138) | *Taraxacum* spp. | 25 |
|  | *Cichorium intybus* L. | 12 |
|  | *Urtica dioica* subsp. *dioica* | 7 |
|  | *Corylus avellana* L. | 7 |
| Kidney (319) | *Taraxacum* spp. | 42 |
|  | *Asparagus acutifolius* L. | 23 |
|  | *Urtica dioica* subsp. *dioica* | 20 |
| Musculoskeletal (183) | *Urtica dioica* subsp. *dioica* | 27 |
|  | *Brassica oleracea* L. | 18 |
|  | *Prunus avium* L. | 11 |
| Nervous system (367) | *Matricaria chamomilla* L. | 110 |
|  | *Tilia platyphyllos* Scop. | 43 |
|  | *Melissa officinalis* L. | 28 |
| Ophthalmic (107) | *Matricaria chamomilla* L. | 28 |
|  | *Vaccinium myrtillus* L. | 17 |
|  | *Solanum tuberosum* L. | 14 |
| Oral cavity (232) | *Malva sylvestris* L. | 70 |
|  | *Salvia officinalis* L. | 27 |
|  | *Syzygium aromaticum* (L.) Merr. & L. M. Perry | 15 |
| Reproductive system (134) | *Malva sylvestris* L. | 19 |
|  | *Petroselinum crispum* (Mill.) Fuss | 17 |
|  | *Matricaria chamomilla* L. | 12 |
| Respiratory (786) | *Linum usitatissimum* L. | 72 |
|  | *Castanea sativa* Mill. | 59 |
|  | *Malva sylvestris* L. | 54 |
